# MCT1-dependent mitochondrial lactate import sustains cardiac metabolism

**DOI:** 10.1038/s41586-026-11182-3

**Published:** 2026-10-28

**Authors:** Ahmad A. Cluntun, Joseph R. Visker, Jesse N. Velasco-Silva, James Carrington, Luis Cedeño-Rosario, Giulia C. Napoli, Ji Eon Kim, Thirupura S. Shankar, Marisa J. Brothwell, Rana Hamouche, Álvaro J. Narbona-Pérez, Jing Ling, Ashish G. Toshniwal, Hayden K. Low, Corey N. Cunningham, David R. Eberhardt, Jing Li, Claire E. Stanley, Queren A. Alcantara, Tarun Yadav, Ryan L. Maves, Wojciech I. Swiatek, Nicholas A. Offei, Bhavya Blaze, Sumangala P. Shetty, Dipayan Chaudhuri, Tingting Hong, Jeffery D. Rothstein, Deborah M. Muoio, Joao A. Paulo, Steven P. Gygi, Eleni Tseliou, Sutip Navankasattusas, James E. Cox, Katsuhiko Funai, Stavros G. Drakos, Jared Rutter, Gregory S. Ducker

**Affiliations:** 1 Department of Biochemistry, University of Utah, Salt Lake City, UT USA; 2 Nora Eccles Harrison Cardiovascular Research and Training Institute, University of Utah, Salt Lake City, UT USA; 3 Department of Integrative Bioscience and Biotechnology, Sejong University, Seoul, Republic of Korea; 4 Department of Nutrition and Integrative Physiology, University of Utah, Salt Lake City, UT USA; 5 Department of Biochemistry and Molecular Biology, Rutgers University, Piscataway, NJ USA; 6 Division of Cardiovascular Medicine, Department of Internal Medicine, School of Medicine, University of Utah, Salt Lake City, UT USA; 7 Department of Biomedical Engineering, University of Utah, Salt Lake City, UT USA; 8 Department of Pharmacology and Toxicology, College of Pharmacy, University of Utah, Salt Lake City, UT USA; 9 Department of Neurology, School of Medicine, The Johns Hopkins University, Baltimore, MD USA; 10 Departments of Medicine and Pharmacology and Cancer Biology, and Duke Molecular Physiology Institute, Duke University, Durham, NC USA; 11 Department of Cell Biology, Harvard Medical School, Boston, MA USA; 12 UTAH (Utah Transplant Affiliated Hospitals) Cardiac Transplant Program, University of Utah Healthcare and School of Medicine, Intermountain Medical Center, Salt Lake Veterans Affairs Health Care System, Salt Lake City, UT USA; 13 Metabolomics, Proteomics and Mass Spectrometry Core Facility, University of Utah, Salt Lake City, UT USA; 14 Center for Metabolic Health, University of Utah, Salt Lake City, UT USA; 15 Howard Hughes Medical Institute, University of Utah School of Medicine, Salt Lake City, UT USA; 16 Present Address: Department of Biochemistry and Molecular Biology, Rutgers University, Piscataway, NJ USA; 17 Present Address: Department of Cardiothoracic Surgery, and Section of Molecular Medicine, Department of Internal Medicine, Wake Forest University Health Science Center, Winston-Salem, NC USA

**Keywords:** Metabolomics, Heart failure, Homeostasis

## Abstract

The heart possesses a dynamic capacity to oxidize a variety of fuels, including glucose and lactate, to meet its energetic demands under diverse dietary, exertion and injury stressors^1^. Oxidation of carbohydrate substrates is thought to converge on the cytosolic generation of pyruvate, which is transported by the mitochondrial pyruvate carrier (MPC) for entry into the tricarboxylic acid cycle^2^. Here we find that glucose-derived carbon is oxidized in mouse myocardium even when the MPC is genetically ablated. Using genetic and pharmacological tools, we demonstrate that lactate monocarboxylate transporter 1 (MCT1, encoded by *Slc16a1*) is required for the oxidation of glucose carbon by MPC-deficient myocardium. We further observe that MCT1, traditionally localized to the plasma membrane, also resides in the inner mitochondrial membrane of mouse and human cardiomyocytes. Together with matrix-localized LDHB, MCT1 enables mitochondrial lactate conversion to pyruvate and NADH, directly fueling the electron transport chain. Loss of MCT1 impairs lactate oxidation, disrupts metabolic flexibility and accelerates the development of heart failure with increased mortality after hyperactivation of the neurohormonal axis or ischemia–reperfusion injury. Thus, the existence of a parallel lactate-based pathway for carbohydrate oxidation can sustain cardiac energetics and maintain myocardial structure and function under diverse stress conditions.

## Abstract (web summary)

In mouse myocardium, glucose oxidation persists in the absence of the mitochondrial pyruvate carrier, with mitochondrial MCT1 importing lactate into mitochondria and LDHB oxidizing it to pyruvate and NADH, sustaining cardiac energetics and preventing stress-induced heart failure.

## Main

The oxidation of carbon fuels within the mitochondria is responsible for more than 95% of human ATP production^1,3^. Dietary carbohydrates supply energy beginning with glycolysis, producing pyruvate that is either reduced to lactate in the cytosol by lactate dehydrogenase (LDH), which also regenerates NAD^+^, or is imported into the mitochondria for oxidation by the tricarboxylic acid (TCA) cycle^4^. Circulating lactate turnover exceeds that of glucose in mammalian physiology^5,6^, establishing lactate production as the dominant mode of cytosolic NAD^+^ regeneration. Lactate has the same oxidation state as glucose, is a major circulating oxidative fuel source in both fasted and fed mammalian physiology, and is consumed primarily by the heart, skeletal muscle and kidney^7–9^.

**Fig. 1:**
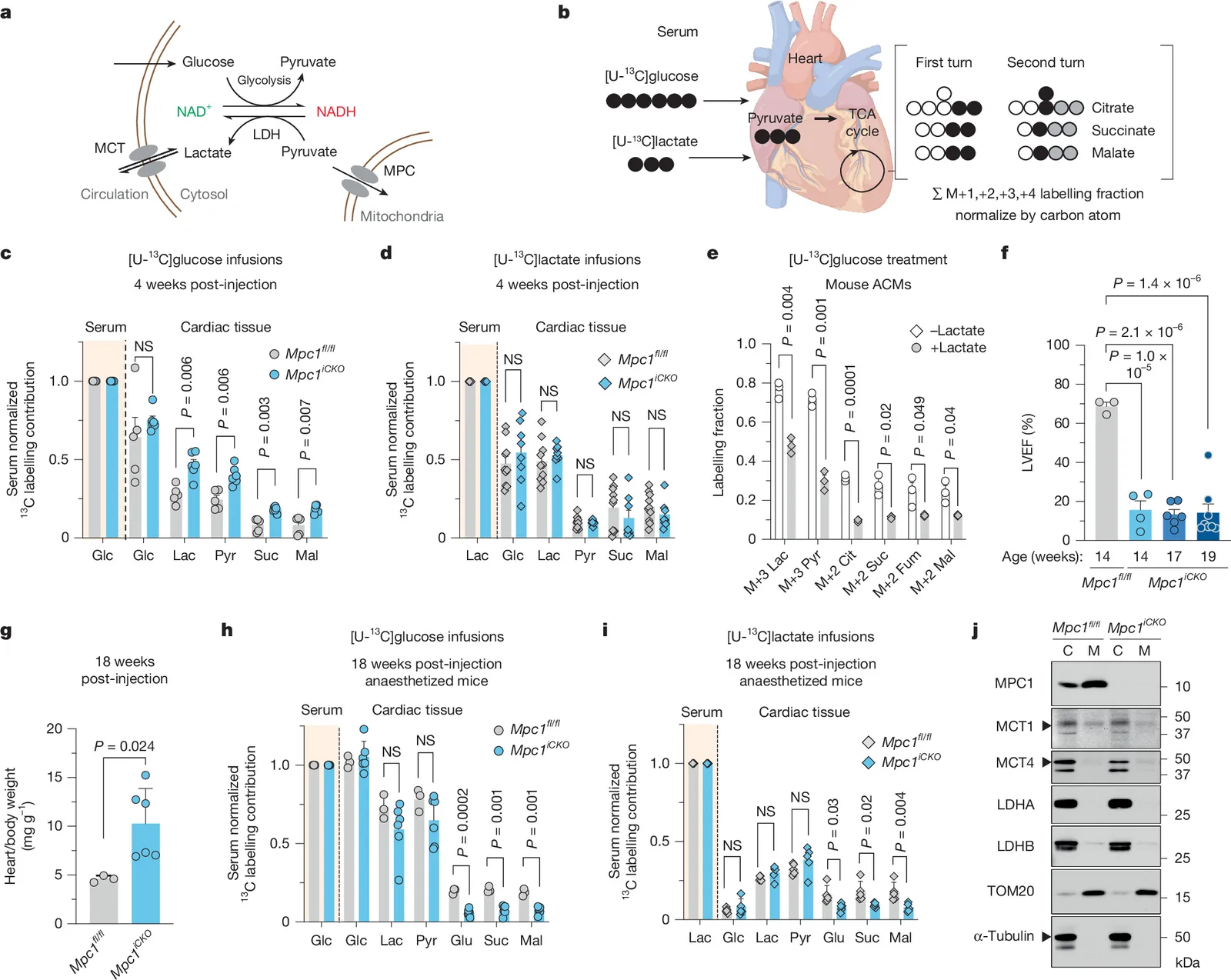
Glucose carbon can enter the TCA cycle independent of the MPC. **a**, Schematic representation of the competition for cytosolic NAD^+^ between glycolysis and lactate oxidation, illustrating that redox-balanced metabolism produces lactate. MCT, lactate monocarboxylate transporter. **b**, Simplified schematic of carbon labelling of heart TCA cycle metabolites resulting from infusion of [U-^13^C]glucose or [U-^13^C]lactate. If the second turn (light grey) incorporates an unlabelled acetyl-CoA, M+1 labelling becomes dominant as is observed in vivo. Created in BioRender; Visker, J. https://BioRender.com/kl2xa6y (2026). **c**, Myocardial ^13^C labelling of lactate, pyruvate and TCA cycle metabolites following [U-^13^C]glucose infusion in awake 12-week-old 4 wpi *Mpc1*^*iCKO*^ (*n* = 10) and *Mpc1*^*fl/fl*^ (*n* = 8) mice. Glc, glucose; Lac, lactate; Mal, malate; Pyr, pyruvate; Suc, succinate. **d**, Myocardial ^13^C metabolite labelling following [U-^13^C]lactate infusion in awake 4 wpi *Mpc1*^*iCKO*^ (*n* = 10) and *Mpc1*^*fl/fl*^ (*n* = 8) mice. **e**, ^13^C labelling of metabolites from primary cultured ACMs incubated with [U-^13^C]glucose with or without 1.6 mM lactate in DMEM, *n* = 3 wells. **f**, LVEF at 14 wpi with tamoxifen of *Mpc1*^*fl/fl*^ (*n* = 3), and 14 wpi (*n* = 4), 17 wpi (*n* = 6) and 19 wpi (*n* = 8) *Mpc1*^*iCKO*^ mice. **g**, Heart weight normalized to body weight from *Mpc1*^*fl/fl*^ (*n* = 3) and *Mpc1*^*iCKO*^ mice at 18–19 wpi (*n* = 6). **h**, Myocardial ^13^C metabolite labelling following [U-^13^C]glucose infusion in 18 wpi *Mpc1*^*iCKO*^ (*n* = 6) and *Mpc1*^*fl/fl*^ (*n* = 3) mice with heart failure. **i**, Myocardial ^13^C metabolite labelling following [U-^13^C]lactate infusion in anaesthetized 18 wpi *Mpc1*^*iCKO*^ (*n* = 5) and *Mpc1*^*fl/fl*^ (*n* = 5) mice. **j**, Immunoblots of the indicated proteins from cytosolic (C) and mitochondrial (M) lysates from *Mpc1*^*iCKO*^ and *Mpc1*^*fl/fl*^ hearts at 18 wpi. Data are mean ± s.e.m. P values from multiple comparisons-corrected (Holm–Sídák method) unpaired two-tailed *t*-tests (**c**–**e**,**g**–**i**) or one-way ANOVA with Dunnett’s multiple comparison test (**f**). NS, not significant (*P* ≥ 0.05). Source Data

Conventional models posit that lactate oxidation starts with the generation of cytosolic pyruvate by LDH, which is subsequently imported into the mitochondria by the obligate heterodimeric mitochondrial pyruvate carrier (MPC, encoded by *Mpc1* and *Mpc2*)^2,10^. This arrangement leads to competition between LDH and glycolytic enzymes for NAD^+^, restricting flux through these pathways when metabolic demand is high (Fig. 1a). However, the heart can oxidize both lactate and glucose simultaneously in response to energetic demand^11–13^, suggesting that there might be distinct routes for pyruvate and lactate oxidation^14,15^.

Here we reconcile the conflict between lactate, glucose and pyruvate oxidation by demonstrating that MCT1, which is canonically on the plasma membrane, is also present on the inner mitochondrial membrane (IMM) of cardiomyocytes. MCT1 is necessary for the uptake and oxidation of lactate, providing metabolic redundancy that sustains cardiac energetics. Genetic ablation of MCT1 abolishes mitochondrial lactate oxidation, disrupts adaptation to neurohormonal, hemodynamic or ischemic stress and accelerates the development of heart failure with enhanced mortality.

## Glucose oxidation independent of the MPC

**Extended Data Fig. 1:**
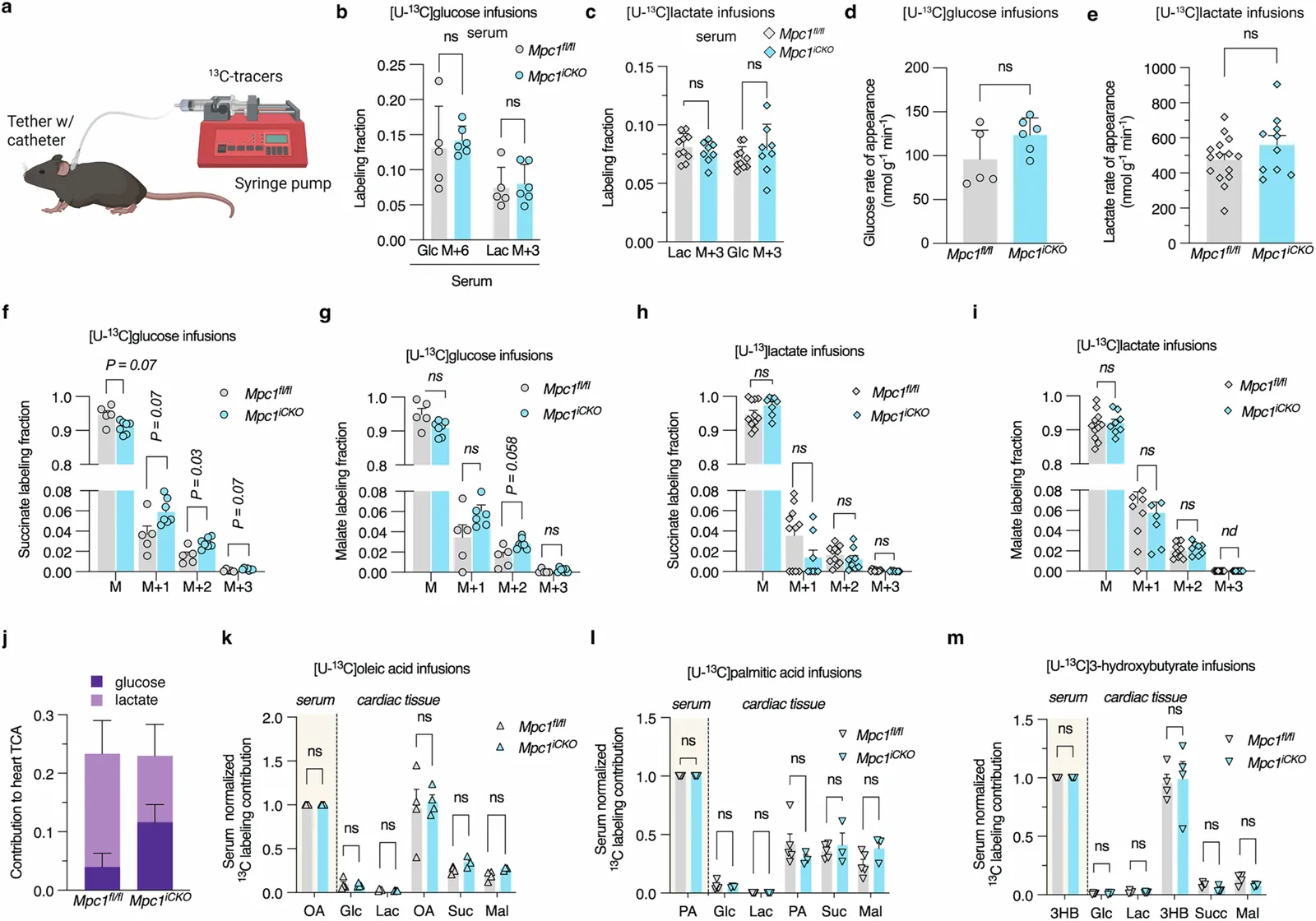
Cardiac fuel usage in *Mpc1*^*iCKO*^ mice. **a**, Schematic showing the awake infusion experiment setup for mouse. Created in BioRender; Visker, J. https://BioRender.com/xdr316w (2026). **b**, Serum glucose and lactate labelling fractions after 3 hrs [U-^13^C]glucose infusion in fasted mice (*Mpc1*^*fl/fl*^ *n* = *5*, *Mpc1*^*iCKO*^ *n* = *6*). **c**, Serum glucose and lactate labelling fraction after 2 h [U-^13^C]lactate infusion in fasted mice (*Mpc1*^*fl/fl*^ *n* = *10*, *Mpc1*^*iCKO*^ *n* = *8*). **d**, Glucose and **e**, lactate rate of appearance calculated from steady-state serum labelling fractions. **f**, Myocardial succinate and **g**, malate ^13^C labelling fractions from [U-^13^C]glucose infusions (*Mpc1*^*fl/fl*^ *n* = *5*, *Mpc1*^*iCKO*^ *n* = *6*) **h**, Myocardial succinate and **i**, malate heart ^13^C labelling fractions from [U-^13^C]lactate infusions (*Mpc1*^*fl/fl*^ *n* = *11*, *Mpc1*^*iCKO*^ *n* = *8*). **j**, Calculated direct nutrient contribution from circulating glucose and lactate to cardiac TCA cycle metabolism (based on succinate and malate labelling data) in *Mpc1*^*fl/fl*^ and *Mpc1*^*iCKO*^ animals (error bars represent standard error). **k**, Serum normalized ^13^C labeling of myocardial lactate, pyruvate and TCA cycle metabolites from [U-^13^C]oleic acid (OA) infusions in *Mpc1*^*iCKO*^ (*n* = *4*) and *Mpc1*^*fl/fl*^ (*n* = *4*) mice. **l**, Serum normalized ^13^C labeling of myocardial lactate, pyruvate and TCA cycle metabolites from [U-^13^C]palmitic acid (PA) infusions in *Mpc1*^*iCKO*^ (*n* = *3*) and *Mpc1*^*fl/fl*^ (*n* = *5*) mice. **m**, Serum normalized ^13^C labeling of myocardial lactate, pyruvate and TCA cycle metabolites from [U-^13^C]3-hydroxybutyrate infusions in *Mpc1*^*iCKO*^ (*n* = *4*) and *Mpc1*^*fl/fl*^ (*n* = *4*) mice. Glc: glucose; Lac: lactate; Suc: succinate; Pyr: pyruvate; Mal: Malate. All data represent mean ± SEM. Significance determined by unpaired two-tailed t tests (d,e), multiple comparisons corrected (Holm-Sídák method) unpaired two-tailed t tests (a,b, f-I, k-m). ns, not significant (p > 0.05). Unmarked comparisons are ns. Source Data

To test the prevailing model of lactate oxidation in heart, we utilized our previously described tamoxifen-inducible cardiac-specific *Mpc1*-knockout mice (*Mpc1*^*fl/fl*^*; αMHC*^*MerCreMer+/−*^, hereafter *Mpc1*^*iCKO*^) to quantify the extent to which the oxidation of glucose and lactate in the myocardium requires the MPC^16^. We implanted jugular vein catheters into 4 week post-tamoxifen injection (wpi) *Mpc1*^*iCKO*^ mice and *Mpc1*^*fl/fl*^ littermate controls with normal cardiac function and subsequently infused awake mice with [U-^13^C]glucose or [U-^13^C]lactate to reach stable serum enrichment (Extended Data Fig. 1a). Hearts and serum were then collected for isotopic labelling analysis of metabolites by liquid chromatography–mass spectrometry (LC–MS) (Fig. 1b). Similar to prior reports, we observed a rapid appearance of [1,2,3-^13^C]lactate in serum upon [U-^13^C]glucose infusion and labelled glucose with [U-^13^C]lactate infusion, reflecting high rates of interconversion between these metabolites^6^ (Extended Data Fig. 1b,c). There was no difference in total serum isotopic enrichment and endogenous rates of glucose and lactate appearance between *Mpc1*^*fl/fl*^ and *Mpc1*^*iCKO*^ mice (Extended Data Fig. 1b–e). We anticipated that the contribution of glucose to TCA cycle metabolites and thus cardiac ATP production would decrease in *Mpc1*^*iCKO*^ mice. Unexpectedly, we observed that the glucose carbon contribution to lactate, pyruvate and TCA cycle metabolites increased in *Mpc1*^*iCKO*^ mice compared with controls, a finding also reported by others^17,18^ (Fig. 1c). ^13^C labelling in the M+1 and M+2 isotopomers of representative TCA metabolites succinate and malate from *Mpc1*^*iCKO*^ hearts was increased, but no changes in M+3 isotopomer labelling were seen, indicating enhanced glucose contribution through acetyl-CoA entry to the TCA cycle^19^ (Extended Data Fig. 1f,g). The contribution of lactate carbon to TCA cycle metabolites trended lower in *Mpc1*^*iCKO*^ hearts but was not significantly different for any individual metabolite (Fig. 1d and Extended Data Fig. 1h,i). Using a linear algebra-based approach^6^, our combined glucose and lactate infusion data showed that *Mpc1*^*iCKO*^ hearts used the same total amount of carbohydrate-sourced fuel to supply the TCA cycle, but that it was paradoxically shifted towards glucose in MPC1-deficient hearts (Extended Data Fig. 1j). We performed additional infusions of other major cardiac fuels (palmitate, oleate and 3-hydroxybutyrate) to determine whether *Mpc1*^*iCKO*^ mice displayed systemic alterations in cardiac fuel choice that may have confounded our glucose and lactate tracing results (Extended Data Fig. 1k–m). However, the contributions of these fuels to the cardiac TCA cycle were not statistically different from controls, suggesting that heart glucose metabolism is resilient to loss of the MPC in the short term.

## Lactate is a fuel for cardiomyocytes

**Extended Data Fig. 2:**
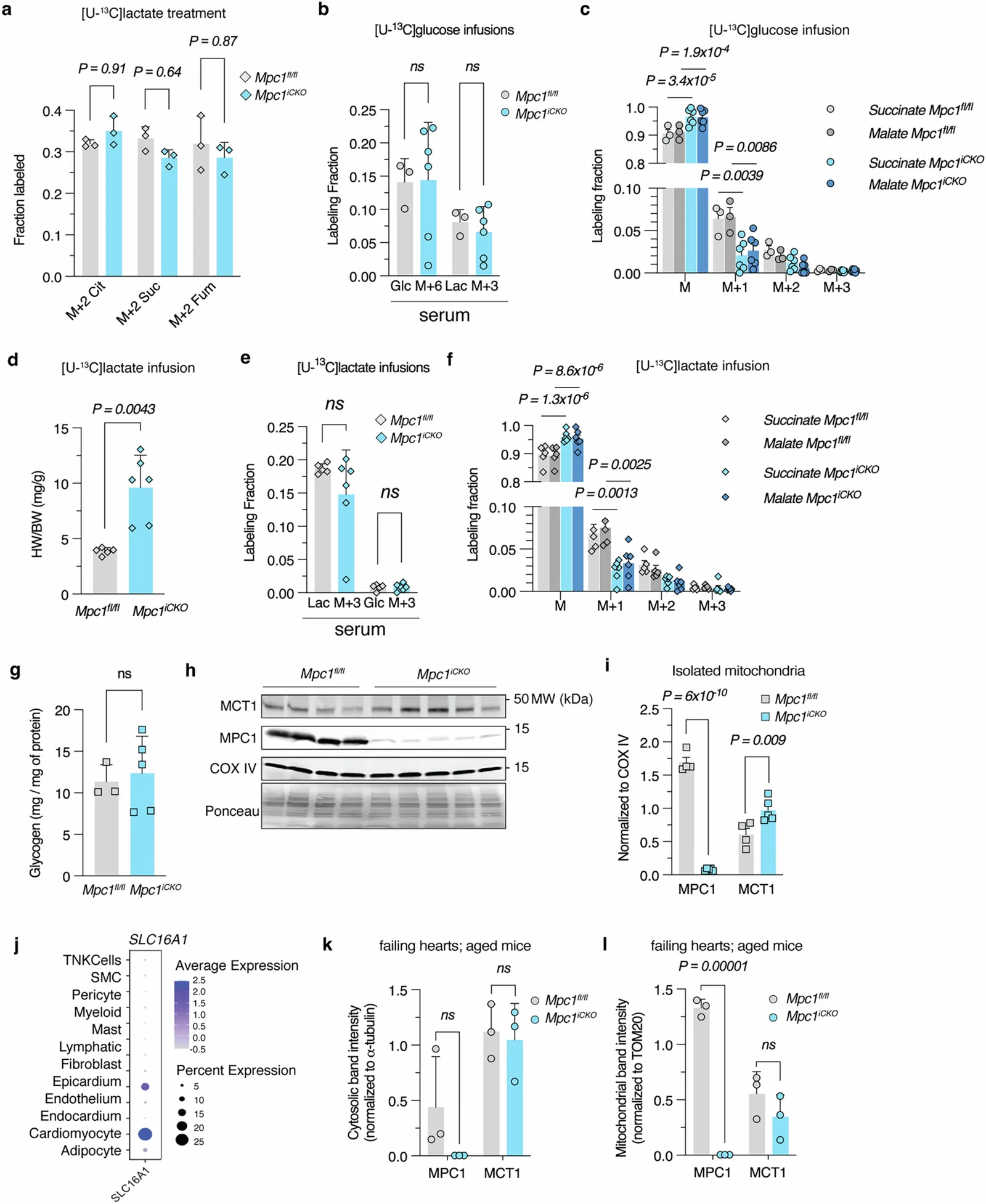
Adult cardiomyocytes preferentially metabolize lactate over glucose. **a**, Isotope corrected M + 2 labeling fractions of TCA cycle intermediates from ACMs treated with [U-^13^C]lactate, *n* = *3* wells. **b**, Serum glucose and lactate labelling fractions after 1 hr [U-^13^C]glucose tail vein infusion in fasted aged *Mpc1*^*fl/fl*^ *(n* = *3)* or *Mpc1*^*iCKO*^ *(n* = *6)*. **c**, ^13^C-enrichment of succinate and malate from myocardium infused with [U-^13^C]glucose (*Mpc1*^*fl/fl*^ *(n* = *3)* or *Mpc1*^*iCKO*^ *(n* = *6)*). **d**, Heart weight (mg) normalized to body weight (g) of aged *Mpc1*^*fl/fl*^ *(n* = *5) and Mpc1*^*iCKO*^ *(n* = *6)* mice infused with [U-^13^C]lactate. **e**, Serum glucose and lactate labelling fractions after 1 hr [U-^13^C]lactate tail vein infusions in aged *Mpc1*^*fl/fl*^ *(n* = *5)* or *Mpc1*^*iCKO*^ *(n* = *6)* mice. **f**, ^13^C-enrichment of succinate and malate from myocardium infused with [U-^13^C]lactate (*Mpc1*^*fl/fl*^*: n* = *5, Mpc1*^*iCKO*^*: n* = *6)*. **g**, Glycogen abundance (mg/mg or protein) from aged *Mpc1*^*fl/fl*^ *(n* = *3)* or *Mpc1*^*iCKO*^ *(n* = *5)* hearts. **h**, Expression of *SLC16A1* transcripts from single-nuclei data^24^ from donor hearts mapped to cell types. **i**, Immunoblot and ponceau stain and **j**, quantification of Mct1 and Mpc1 in isolated mitochondria from *Mpc1*^*fl/fl*^ *(n* = *4)* or *Mpc1*^*iCKO*^ *(n* = *5)*, 4 weeks post injection. All data represent mean ± SEM. Significance determined by multiple comparisons corrected (Holm-Sídák method) unpaired two-tailed t tests (a-b,e,i,k,l), two-way ANOVA with Tukey’s multiple comparison correction (c,f) or two-tailed t-tests (d,g). ns, not significant (p > 0.05). Unmarked comparisons are ns. Source Data

Our data from young *Mpc1*^*iCKO*^ mice suggests the presence of an MPC-independent mitochondrial import pathway for glucose and lactate carbons. To better understand the cardiomyocyte-intrinsic metabolism of glucose and lactate, we used primary cultured adult cardiomyocytes (ACMs). Control ACMs were competent to oxidize glucose as assessed by ^13^C incorporation into TCA cycle metabolites (Fig. 1e). However, when unlabelled lactate was added to standard lactate-free ACM culture medium (DMEM) containing [U-^13^C]glucose, labelling from glucose was attenuated, suggesting a strong preference for lactate over glucose oxidation (Fig. 1e). Lactate consumption was not affected by loss of MPC1 as ACMs from *Mpc1*^*iCKO*^ and *Mpc1*^*fl/fl*^ mice metabolized lactate into TCA metabolites equivalently (Extended Data Fig. 2a).

*Mpc1*^*iCKO*^ mice develop heart failure^16,17,20,21^ and we investigated whether changes in glucose and lactate oxidation were now present. *Mpc1*^*iCKO*^ mice at 16–20 wpi exhibited reduced left ventricular ejection fraction (LVEF) and hypertrophy (Fig. 1f,g). To successfully introduce stable isotopes into these mice with heart failure, we adopted our protocol to employ a tail vein catheter infusion administered under anaesthesia. Oxidation of glucose and lactate carbon was significantly reduced in failing *Mpc1*^*iCKO*^ hearts, despite similar pyruvate and lactate labelling indicating that carbohydrate uptake was not altered (Fig. 1h,i and Extended Data Fig. 2b–f). Total cardiac glycogen was unchanged (Extended Data Fig. 2g). The observed reduction in the oxidation of infused lactate in failing *Mpc1*^*iCKO*^ hearts support the hypothesis that there is an MPC1-independent path for lactate oxidation that supports cardiac function.

To identity a potential alternative mitochondrial transporter of lactate, we conducted immunoblot analysis of purified mitochondria from myocardium and observed expression of the lactate transporter MCT1 (encoded by *Slc16a1*) in the mitochondrial fraction of healthy and failing *Mpc1*^*iCKO*^ hearts (Fig. 1j and Extended Data Fig. 2h,i). MCT1 has previously been reported in mitochondria from cardiac tissue^22,23^. Analysis of published single-nuclei RNA-sequencing data from human hearts showed that *SLC16A1* transcripts are predominantly expressed in cardiomyocytes^24^ (Extended Data Fig. 2j). Notably, MCT1 expression was increased in the mitochondria of young healthy *Mpc1*^*iCKO*^ hearts but not failing hearts (Extended Data Fig. 2i,k,l). The existence of mitochondrial lactate import and metabolism has been proposed for decades, but its physiological relevance and mechanistic basis remain actively contested within the field^25,26^.

## MCT1 is required for lactate oxidation

**Fig. 2:**
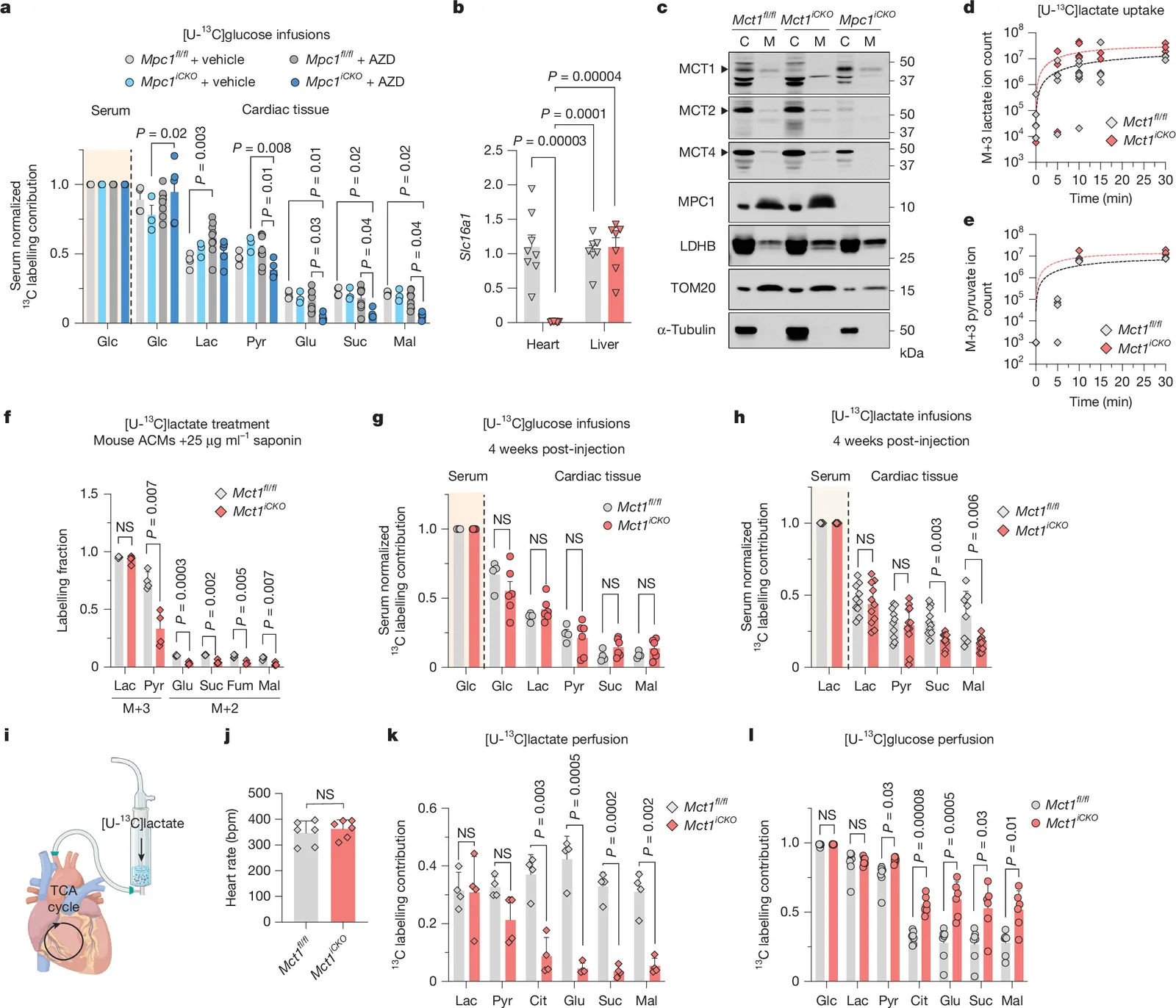
MCT1 is required for the oxidation of lactate. **a**, Myocardial ^13^C labelling of glycolytic and TCA cycle metabolites from *Mpc1*^*fl/fl*^ and *Mpc1*^*iCKO*^ mice treated with either AZD or vehicle and infused with [U-^13^C]glucose (*Mpc1*^*fl/fl*^ vehicle *n* = 4, *Mpc1*^*iCKO*^ vehicle *n* = 3, *Mpc1*^*fl/fl*^ AZD *n* = 8, *Mpc1*^*iCKO*^ *n* = 5). **b**, Expression of *Slc16a1* transcripts in heart and liver tissue from *Mct1*^*fl/fl*^ (*n* = 8) and *Mct1*^*iCKO*^ (*n* = 7) mice quantified by real-time quantitative PCR. **c**, Representative immunoblots of the indicated proteins from mitochondrial and cytoplasmic fractions of *Mct1*^*fl/fl*^, *Mct1*^*iCKO*^ and *Mpc1*^*iCKO*^ hearts. Experiment repeated on three independent sets of mice. **d**, M+3 lactate ion counts from freshly isolated primary adult cardiomyocytes from *Mct1*^*fl/fl*^ and *Mct1*^*iCKO*^ hearts incubated with [U-^13^C]lactate for indicated times. **e**, M+3 pyruvate ion counts from the same experiment as **d**. **f**, ^13^C labelling of pyruvate and TCA cycle metabolites from *Mct1*^*fl/fl*^ or *Mct1*^*iCKO*^ ACMs incubated with [U-^13^C]lactate after 5 min saponin treatment. *n* = 4. **g**, Myocardial ^13^C metabolite labelling following [U-^13^C]glucose infusions in awake *Mct1*^*fl/fl*^ (*n* = 4) or *Mct1*^*iCKO*^ (*n* = 6) mice. **h**, Myocardial ^13^C metabolite labelling following [U-^13^C]lactate infusions in awake *Mct1*^*fl/fl*^ (*n* = 10) or *Mct1*^*iCKO*^ (*n* = 11) mice. **i**, Schematic representation of ex vivo heart perfusions. Created in BioRender; Carrington, J. https://BioRender.com/ps8ckjv (2026). **j**, Heart rates of *Mct1*^*fl/fl*^ (*n* = 6), or *Mct1*^*iCKO*^ (*n* = 6) mice 1 min prior to termination of ex vivo perfusions with ^13^C-labelled lactate. bpm, beats per minute. **k**, ^13^C labelling of metabolites from [U-^13^C]lactate perfusions in *Mct1*^*fl/fl*^ (*n* = 4) or *Mct1*^*iCKO*^ (*n* = 4) hearts. **l**, ^13^C labelling of metabolites from [U-^13^C]glucose perfusions in *Mct1*^*fl/fl*^ (*n* = 7) or *Mct1*^*iCKO*^ (*n* = 6) hearts. Data are mean ± s.e.m. *P* values from two-way ANOVA (**a**,**b**) or multiple comparisons-corrected (Holm–Sídák method) unpaired two-tailed *t*-tests (**f**–**h**,**k**,**l**). Source Data

**Extended Data Fig. 3:**
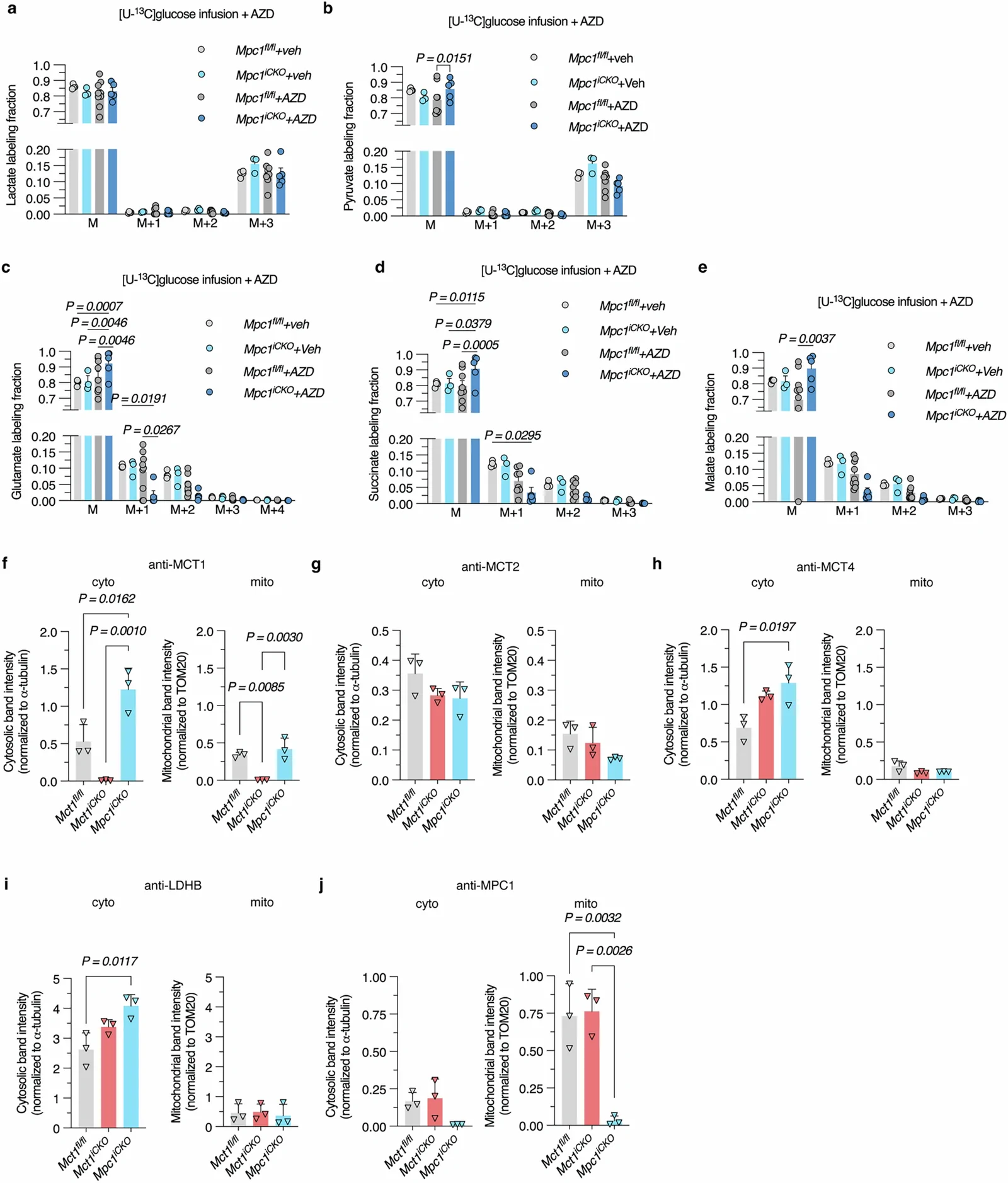
Inhibition of MCT1 blocks glucose utilization in *Mpc1*^*iCKO*^ hearts. **a**, Lactate, **b**, pyruvate **c**, glutamate, **d**, succinate, and **e**, malate labelling fraction from [U-^13^C]glucose infused myocardium with and without co-administration of AZD inhibitor (*Mpc1*^*fl/fl*^ Veh *n* = *4*, *Mpc1*^*iCKO*^ Veh *n* = *3*, *Mpc1*^*fl/fl*^ AZD *n* = *8*, *Mpc1*^*iCKO*^ *n* = 5). Quantification of **f**, MCT1, **g**, MCT2, **h**, MCT4, **i**, LDHB, **j**, MPC1, from immunoblots of cytoplasmic and mitochondrial fractions of *Mct1*^*fl/fl*^, *Mct1*^*iCKO*^, and *Mpc1*^*iCKO*^ hearts normalized to α-tubulin or TOM20 respectively. (*n* = *3* hearts). All data represent mean ± SEM. Significance determined by two-way ANOVA with Tukey’s multiple comparison correction (a-e) or one-way ANOVA with Dunnett’s multiple comparison test (f-j). ns, not significant (p > 0.05). Unmarked comparisons are ns. Source Data

To test whether MCT1 can provide an alternative mechanism for carbohydrate carbon entry and oxidation in mitochondria, we repeated our [U-^13^C]glucose tracing in young, healthy *Mpc1*^*iCKO*^ mice cotreated with AZD3965 (AZD), a potent MCT1 inhibitor with selectivity over MCT2^27^. AZD treatment in control mice had no effect on the oxidation of glucose carbon as measured by TCA cycle metabolite labelling (Fig. 2a and Extended Data Fig. 3a–e). However, when AZD was administered to *Mpc1*^*iCKO*^ mice, glucose labelling of TCA cycle substrates was ablated. Although MCT1 may have a role in the transport of lactate across both the plasma membrane and IMM, our tracing data support an essential mitochondrial role, as glucose carbon uptake is not dependent on MCT1 activity. Overall, our data suggest functional redundancy between MCT1 and the MPC for the uptake of three-carbon equivalents into the mitochondria.

To further explore the role of MCT1 as a mitochondrial lactate transporter, we developed a tamoxifen-inducible, cardiac-specific *Mct1* knockout mouse: *Mct1*^*fl/fl*^*; αMHC*^*MerCreMer+/−*^ (hereafter *Mct1*^*iCKO*^). We validated knockout by PCR and MCT1 immunoblots from whole-heart and mitochondrial preparations (Fig. 2b,c and Extended Data Fig. 3f). Across genotypes, a larger fraction of MCT1 signal was associated with mitochondria than MCT2 or MCT4 (Fig. 2c and Extended Data Fig. 3f–h). When comparing *Mpc1*^*iCKO*^, *Mct1*^*iCKO*^ and control hearts from young mice, we observed an increase in cytosolic and total MCT1, MCT4 and LHDB protein expression in *Mpc1*^*iCKO*^ hearts, suggesting a compensatory lactate metabolism axis (Extended Data Fig. 3f,h,i). No significant changes in the expression of these proteins or MPC1 and MCT2 were observed in *Mct1*^*iCKO*^ hearts (Extended Data Fig. 3f–j).

**Extended Data Fig. 4:**
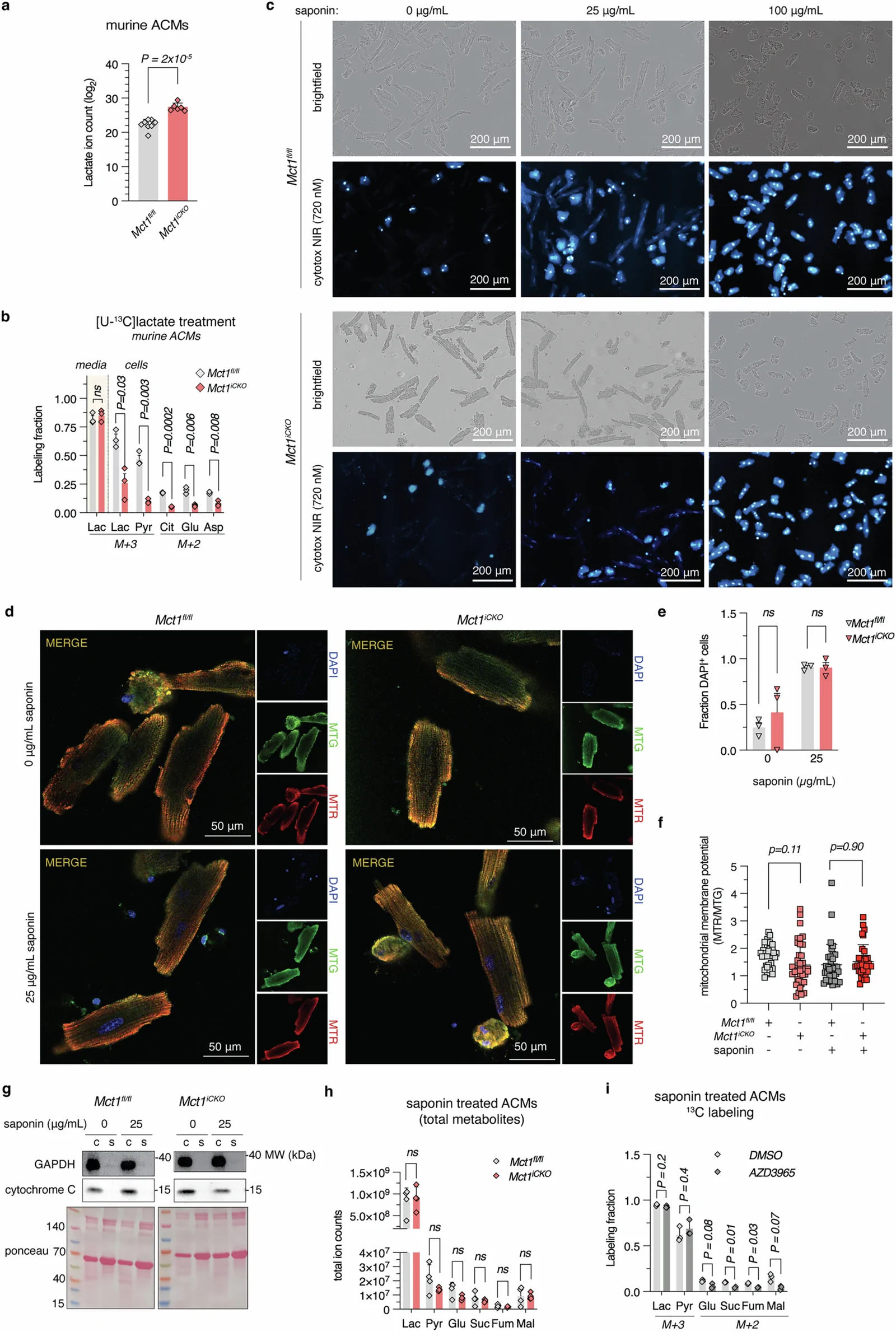
Lactate oxidation in permeabilized adult cardiomyocytes is MCT1 dependent. **a**, Total lactate ion count (log_2_) from ACMs incubated in HPLM media. **b**,^13^C enrichment in TCA cycle metabolites from *Mct1*^*fl/fl*^ or *Mct1*^*iCKO*^ ACMs incubated with [U-^13^C]lactate for 2 h in HPLM (*n* = *3 wells)*. **c**, Brightfield and near-IR imaging of Cytotox NIR treated freshly isolated murine adult cardiomyocytes treated with the indicated saponin concentration for 5 min before washout and addition of dye. Representative brightfield and fluorescence images from Incucyte instrument **d**, Confocal images of ACMs treated with saponin before washout and addition of DAPI, followed by MitoTracker Green FM (MTG) and MitoTracker Red CMXRos (MTR) dyes. Imaging was conducted after 20 min of incubation and washes with MAB at 40x magnification on a Zeiss LSM 880 Airyscan confocal microscope (representative images from *n* = *3* wells imaged/ condition). **e**, Quantification of DAPI+ cells (*n* = *3 wells*). **f**, Quantification of mitochondrial membrane potential based on mean fluorescence intensity of MTR/MTG in saponin-treated cardiomyocytes (*n* = *32-35 individual ACMs*, significance by Welch’s t-test). **g**, Immunoblots of cytochrome C and GAPDH from supernatants (s) or cells (c) upon treatment with saponin for 5 min followed by a 10 min incubation. Ponceau stain for total protein loading. **h**, Total ion counts of lactate, pyruvate and TCA metabolites from Fig. 2f (*n* = *4*). **i**, ^13^C enrichment in TCA cycle metabolites from *Mct1*^*fl/fl*^ ACMs treated with AZD3965 and incubated with [U-^13^C]lactate for 10 min after 5 min saponin (25 μg/mL) treatment (*n* = *3 wells*). Lac: lactate; Pyr: pyruvate; Suc: succinate; Fum: fumarate; Mal: Malate; Glu: glutamate; Asp: aspartate. All data represent mean ± SEM. Significance determined by unpaired two-tailed t tests (b) or multiple comparisons corrected (Holm-Sídák method) unpaired two-tailed t tests (c,f,g,i,j). ns, not significant (p > 0.05). Unmarked comparisons are ns. Source Data

We incubated ACMs from *Mct1*^*fl/fl*^ (control) and *Mct1*^*iCKO*^ mice with [U-^13^C]lactate to test whether cytosolic uptake of lactate is lost without MCT1. The rate of cytosolic uptake of lactate and production of labelled pyruvate was unaffected by loss of MCT1, suggesting that the observed oxidative phenotypes were unlikely to be the result of changes in cytosolic import (Fig. 2d,e). At steady state, lactate contributed robustly to citrate (M+2) labelling in control ACMs, but this was sharply reduced in *Mct1*^*iCKO*^ ACMs. The overall lactate labelling fraction was also reduced, but this was owing to a sharp increase in the total levels of lactate present (Extended Data Fig. 4a,b). To further investigate whether changes in mitochondrial uptake and oxidation of lactate were dependent on MCT1 and independent of its known roles on the plasma membrane, we treated isolated ACMs with saponin to selectively permeabilize the plasma membrane^28^ (Extended Data Fig. 4c,d). Although plasma membrane integrity was disrupted, mitochondria membrane potential was maintained and cytochrome *c* was not released (Extended Data Fig. 4d–g). Upon incubation with [U-^13^C]lactate, we observed robust oxidation in *Mct1*^*fl/fl*^ ACMs, as evidenced by TCA cycle labelling that was attenuated in *Mct1*^*iCKO*^ cells and upon treatment with AZD3965 (Fig. 2f and Extended Data Fig. 4h,i).

**Extended Data Fig. 5:**
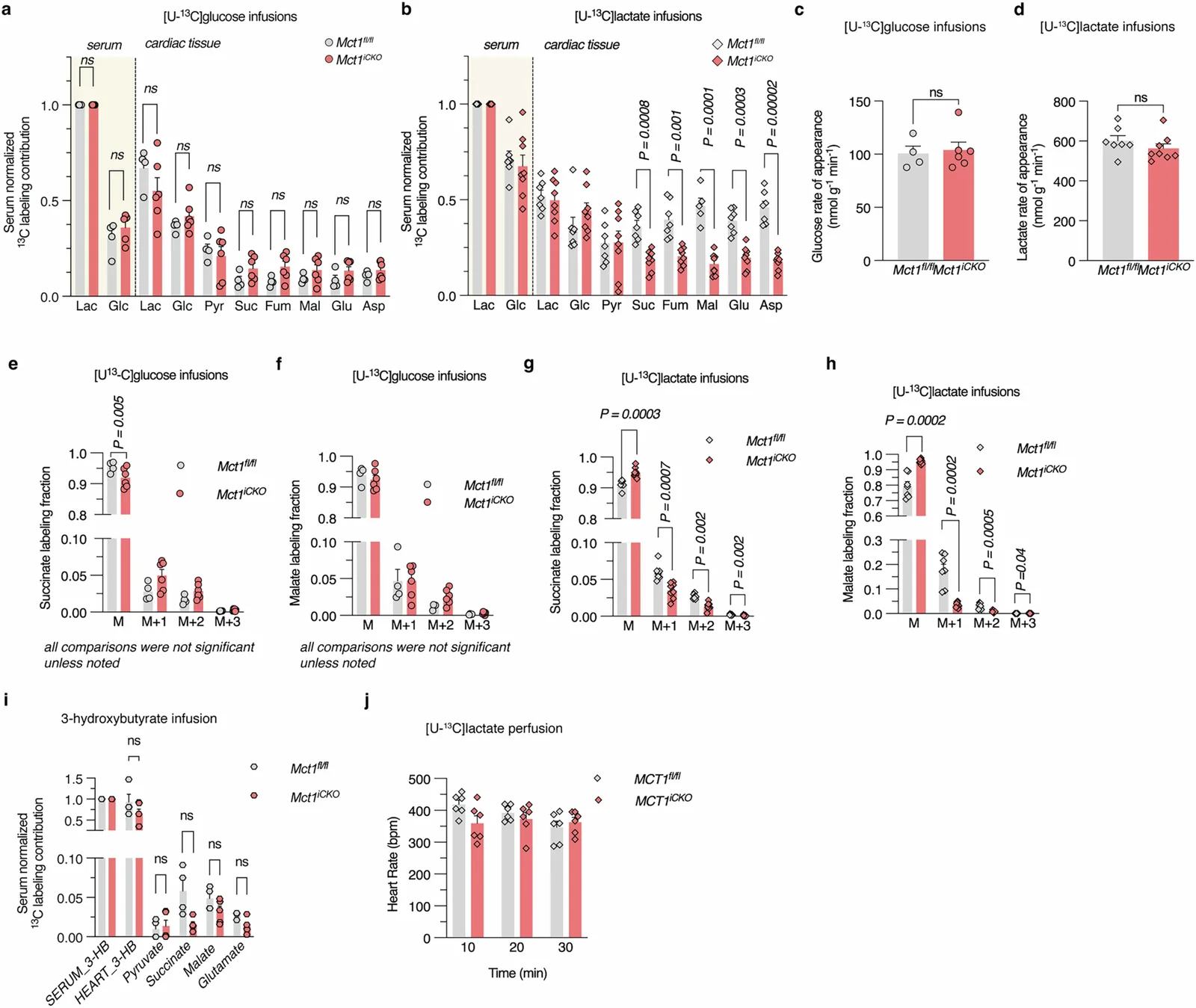
Cardiac fuel usage in *Mct1*^*iCKO*^ mice. **a**, Myocardial ^13^C labeling of glucose, lactate, pyruvate and TCA cycle metabolites from [U-^13^C]glucose infusions in *Mct1*^*fl/fl*^ (*n* = *4*), or *Mct1*^*iCKO*^ (*n* = *6*) mice. **b**, Myocardial ^13^C labeling of glucose, pyruvate, lactate and TCA cycle metabolites from [U-^13^C]lactate infusions in *Mct1*^*fl/fl*^ (*n* = *7*) or *Mct1*^*iCKO*^ (*n* = *8*) mice. **c**, Glucose and **d**, lactate rate of appearance calculated from steady state labelling fractions. **e**, Succinate and **f**, malate ^13^C labelling fractions from [U-^13^C]glucose infused myocardium (*Mct1*^*fl/fl*^ *n* = *4*, *Mct1*^*iCKO*^ *n* = *6*). **g**, Succinate and **h**, malate ^13^C labelling fractions from [U-^13^C]lactate infused myocardium (*Mct1*^*fl/fl*^ *n* = *7*, *Mct1*^*iCKO*^ *n* = *8*). **i**. Myocardial ^13^C labeling of pyruvate and TCA cycle metabolites from [U-^13^C]3-hydroxybutyrate infusions in *Mct1*^*fl/fl*^ (*n* = *4*), or *Mct1*^*iCKO*^ (*n* = *4*) mice. **j**, Measured heart rate of *Mct1*^*fl/fl*^ (*n* = *6)* and *Mct1*^*iCKO*^ *(n* = *6*) hearts during [U-^13^C]lactate Langendorff perfusions (ex vivo). Glc: glucose; Lac: lactate; Pyr: pyruvate; Suc: succinate; Fum: fumarate; Mal: Malate; Glu: glutamate; Asp: aspartate. All data represent mean ± SEM. Significance determined by unpaired two-tailed t tests (c,d), multiple comparisons corrected (Holm-Sídák method) unpaired two-tailed t tests (a,b,e-i), two-way ANOVA with Tukey’s multiple comparison correction (j) ns, not significant (p > 0.05). Unmarked comparisons are ns. Source Data

We infused awake *Mct1*^*iCKO*^ mice with [U-^13^C]glucose or [U-^13^C]lactate to determine whether MCT1 was necessary for the oxidation of lactate in vivo. Serum glucose and lactate metabolic parameters were not different by genotype (Extended Data Fig. 5a–d). In *Mct1*^*iCKO*^ hearts, the entry of lactate carbon into TCA metabolites was significantly reduced, whereas labelling from glucose was unchanged (Fig. 2g,h and Extended Data Fig. 5a,b,e–h). Of note, loss of MCT1 did not alter tissue lactate or pyruvate enrichment, indicating that the observed TCA cycle defects were not due to a loss of plasma membrane lactate transport. Oxidation of 3-hydroxybutyrate was also not affected (Extended Data Fig. 5i). To isolate the effects of MCT1 on lactate oxidation alone, we used an ex vivo perfused heart model (Langendorff) with a non-recirculating solution to prevent isotopic exchange between substrates. We cannulated hearts and perfused them with a working buffer containing the major cardiac energy substrates (glucose, lactate, pyruvate and bovine serum albumin (BSA)-conjugated fatty acids)^11^ (Fig. 2i). Final heart rate was unchanged between *Mct1*^*iCKO*^ and control mice (Fig. 2j and Extended Data Fig. 5j). We traced lactate utilization by replacing the unlabelled lactate with [U-^13^C]lactate (Fig. 2k). Loss of MCT1 prevented nearly all lactate oxidation, but had no effect on lactate enrichment within the tissue. In a second perfusion experiment, we observed that oxidation of [U-^13^C]glucose was increased in *Mct1*^*iCKO*^ hearts, presumably compensating for the loss of lactate oxidation (Fig. 2l).

## MCT1 and LDHB localize to mitochondria

**Fig. 3:**
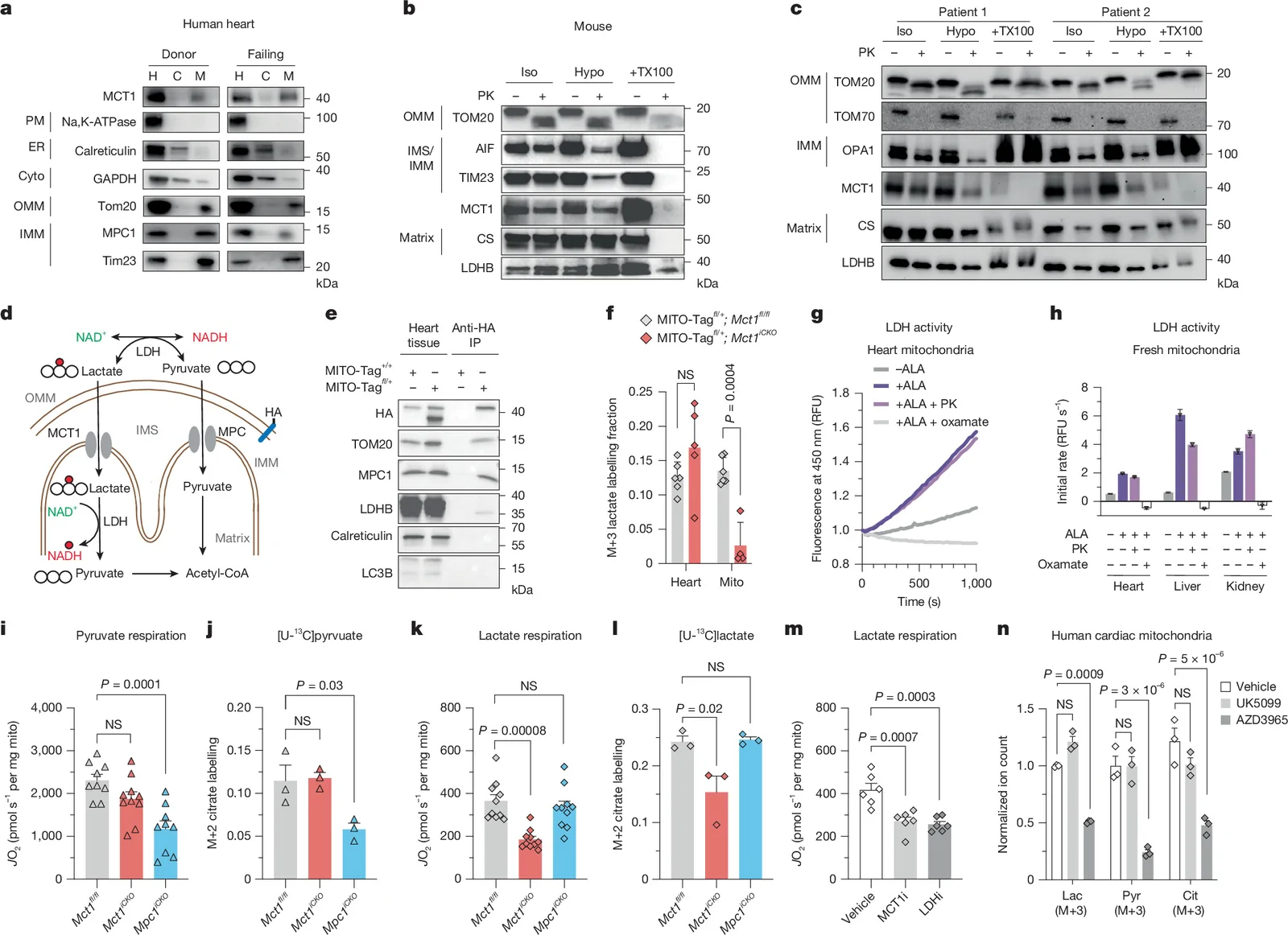
Loss of MCT1 impairs lactate entry and oxidation within mitochondria. **a**, Immunoblots of indicated protein markers from whole heart (H), cleared cytoplasmic fraction (C) and cleared mitochondrial fraction (M) of a representative non-failing donor heart and a heart failure (failing) explant. ER, endoplasmic reticulum. **b**, Immunoblots of indicated protein markers from a proteinase K (PK) protection assay conducted on freshly isolated mitochondria from mouse myocardium. Representative of *n* = 5 experiments. Hypo, hypotonic solution; IMS, intermembrane space; Iso, isotonic solution. TX100, Triton X-100. **c**, Immunoblots of indicated protein markers from a proteinase K protection assay conducted on mitochondria isolated from previously frozen myocardial tissue from two independent human heart failure explants. **d**, Schematic of [2-^2^H]lactate as a tracer for lactate metabolism. **e**, Immunoblots of indicated proteins from *MITO-Tag*^*fl/+*^ myocardium and mitochondrial lysates. Anti-HA IP, anti-haemagglutinin immunoprecipitation. **f**, Lactate M+1 labelling fractions from myocardial tissue and mitochondria from hearts of indicated genotypes subjected to ex vivo perfusion with [2-^2^H]lactate. **g**, LDH activity assays conducted on freshly isolated cardiac mitochondria pretreated with indicated reagents. ALA, alamethicin. **h**, Initial LDH reaction rates from mitochondria isolated from mouse organs with and without permeabilization (calculated from *n* = 3 per condition). Data are mean ± 95% confidence interval. **i**, Oxygen consumption rates (*J*O_2_) of isolated cardiac mitochondria respiring on pyruvate from the indicated genotypes (*Mct1*^*fl/fl*^ *n* = 9, *Mct1*^*iCKO*^ *n* = 10, *Mpc1*^*iCKO*^ *n* = 9). **j**, Citrate M+2 labelling fraction from isolated mouse cardiac mitochondria incubated with [U-^13^C]pyruvate (*Mct1*^*fl/fl*^ *n* = 3, *Mct1*^*iCKO*^ *n* = 3, *Mpc1*^*iCKO*^ *n* = 3). **k**, Oxygen consumption rates of isolated mouse cardiac mitochondria respiring on lactate (*Mct1*^*fl/fl*^ *n* = 10, *Mct1*^*iCKO*^ *n* = 10, *Mpc1*^*iCKO*^ *n* = 10). **l**, Citrate M+2 labelling fraction from isolated mouse cardiac mitochondria incubated with [U-^13^C]lactate (*Mct1*^*fl/fl*^ *n* = 3, *Mct1*^*iCKO*^ *n* = 3, *Mpc1*^*iCKO*^ *n* = 3). **m**, Oxygen consumption rates of isolated mouse cardiac mitochondria respiring on lactate treated with DMSO (*n* = 6), MCT1 inhibitor (MCTi; 7ACC2: *n* = 6) or pan-LDH inhibitor (LDHi; GSK 2837808A: *n* = 6). **n**, Normalized ion counts of lactate (M+3), pyruvate (M+3) and citrate (M+2) from non-failing human donor cardiac mitochondria incubated with [U-^13^C]lactate (*n* = 3 human hearts). Data are mean ± s.e.m. unless indicated. *P* values from multiple comparisons-corrected (Holm–Sídák method) unpaired two-tailed *t*-tests (**f**), one-way ANOVA with Dunnett’s multiple comparison test (**i**–**m**) or two-way ANOVA (**n**). Source Data

**Extended Data Fig. 6:**
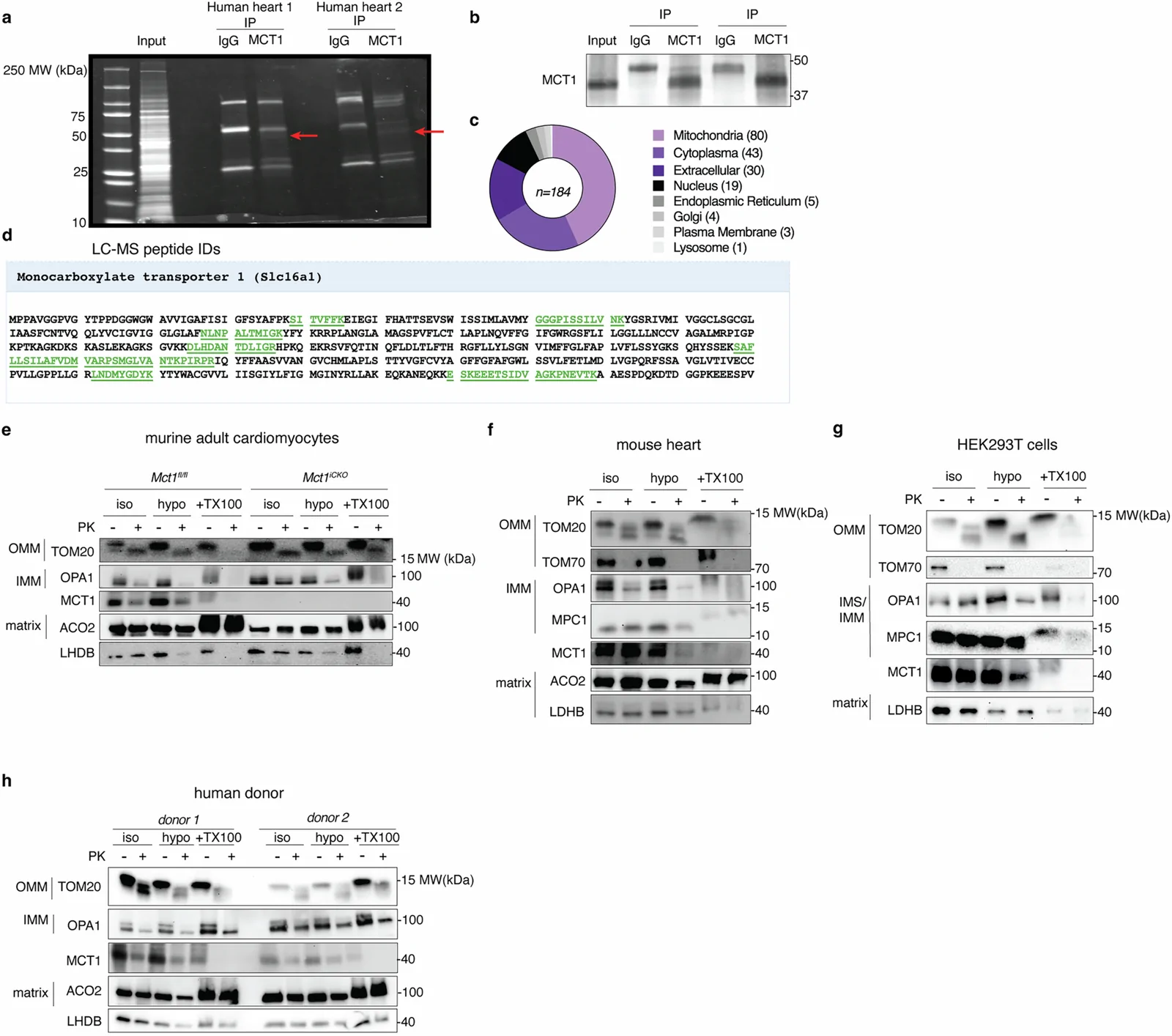
Identification of MCT1 in purified mitochondria. **a**, Coomassie blue stained gel of anti-MCT1 immunoprecipitation from mitochondria purified from two unique human hearts. Eluted immunoprecipitants were run on a SDS Page gel and bands at the correct size (43kD, arrow) were cut out and sent for proteomic analysis. **b**, corresponding immunoblot of MCT1 of the IP experiment. **c**, Summary of unique protein IDs from MCTI-IP performed on purified mitochondria. **d**, Sequence coverage (peptides) of human MCT1 from LC-MS/MS measurements of protein lysates from IP pulldown in b. **e**, Immunoblot of proteinase-K membrane protection assay using murine primary adult cardiomyocytes from *Mct1*^*fl/fl*^ and *Mct1*^*iCKO*^ hearts. Representative of 3 independent isolations. **f**, Immunoblot of proteinase-K membrane protection assay conduced on mitochondria freshly isolated from whole wild-type C57Bl/6 J mouse heart. Representative of 6 independent isolations. **g**, Immunoblot of proteinase-K membrane protection assay using HEK293T cells. Representative of 6 independent experiments. **h**, Immunoblot of proteinase-K membrane protection assay using human donor heart tissue (*n* = *2*).

Paralleling our observations in mouse samples, we detected MCT1 in purified mitochondrial preparations obtained from myocardium of human donors and patients with heart failure (Fig. 3a). To confirm the identity of MCT1 within human mitochondria, we immunoprecipitated MCT1 from purified cardiac mitochondria followed by proteomic analysis. MCT1 peptides were identified together with a high representation of mitochondrial proteins, but not plasma membrane markers, supporting the existence of a mitochondrial MCT1 fraction (Extended Data Fig. 6a–d and Supplementary Table 3). To provide additional evidence of MCT1 and LDHB localization within the mitochondria, we performed proteinase K protection assays using purified mouse and human cardiac mitochondria. In isotonic buffer, intact membranes protect the IMM, intermembrane space and matrix proteins from proteinase K, whereas outer mitochondrial membrane (OMM) proteins are digested. Hypotonic buffer disrupts the OMM, exposing OMM, intermembrane space and some IMM proteins. Detergent (Triton X-100) solubilizes all membranes and denatures protein, rendering all mitochondrial proteins sensitive to proteinase K. Mitochondria from mouse myocardium or isolated cardiomyocytes subjected to these conditions displayed an MCT1 protection pattern resembling that of an IMM protein (Fig. 3b and Extended Data Fig. 6e,f). Across preparations, MCT1 was relatively resistant to proteinase K in isotonic buffer, unlike OMM proteins such as TOM20. In these preparations, we also observed a protected LDHB band that showed similar digestion patterns to citrate synthase, a matrix protein. Similar protection patterns were observed in mitochondria from a human cell line, failing human hearts and non-failing human donor hearts (Fig. 3c and Extended Data Fig. 6g,h). Samples from human hearts showed higher variability and more overall degradation due to sample handling differences including freeze–thaw cycles.

**Extended Data Fig. 7:**
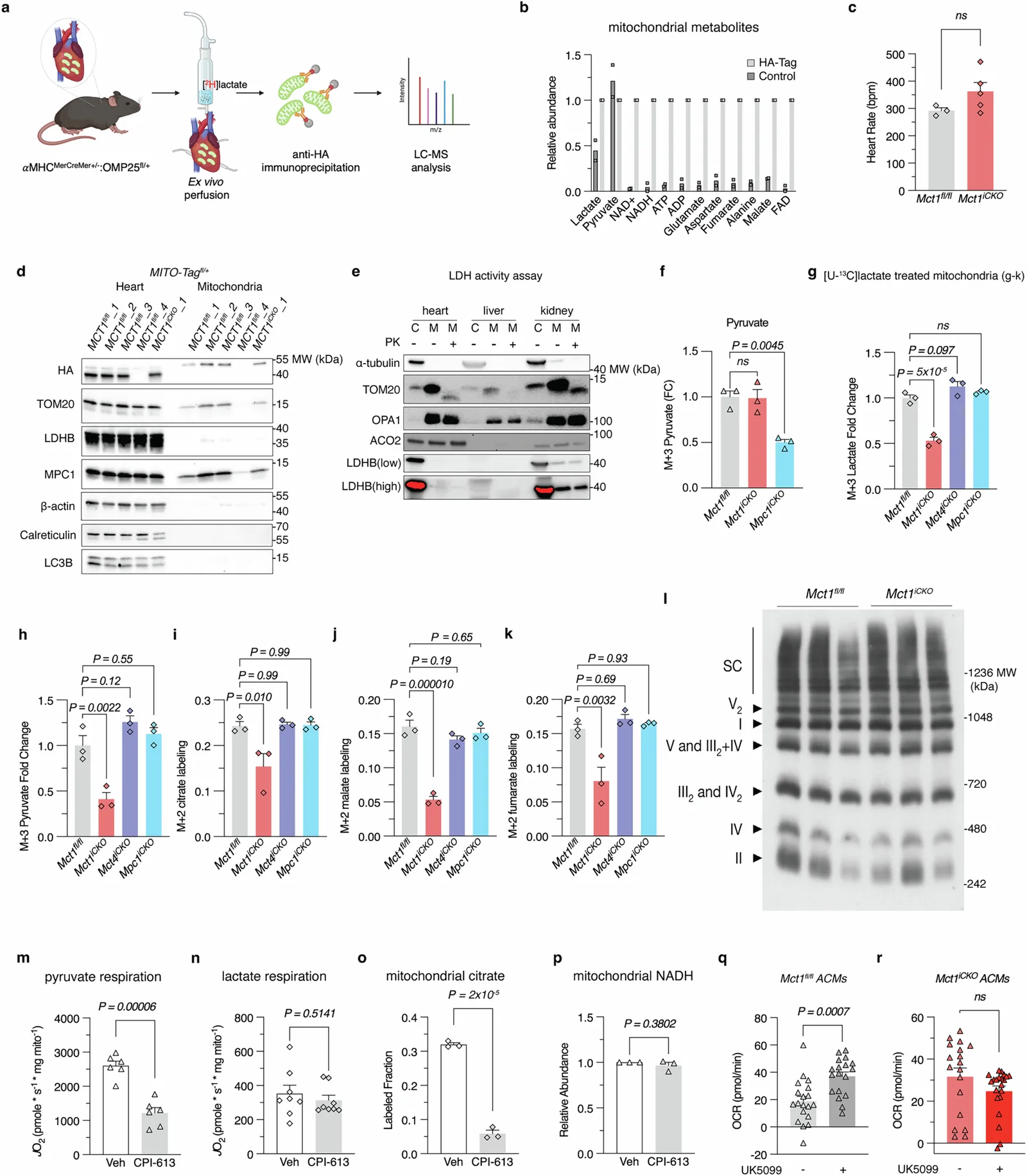
Mitochondrial metabolism and respiration on lactate depends on MCT1. **a**, Schematic representation of ex vivo perfusions with [^2^H]lactate in *αMHC*^*MerCreMer+/−*^*:MITO-Tag*^*fl/+*^ (OMP25) hearts followed by anti-HA immunoprecipitation and LC-MS analysis. Created in BioRender; Visker, J. https://BioRender.com/s2dxuqx (2026). **b**, Relative abundance of mitochondrial metabolites from freshly isolated *αMHC*^*MerCreMer+/−*^*:MITO-Tag*^*fl/+*^ test hearts (*n* = *2* independent extractions). **c**, Heart rate (bpm: beats per minute) in *Mct1*^*fl/fl*^ (*n* = *3)* and *Mct1*^*iCKO*^ *(n* = *5*) from [^2^H]lactate perfusions (ex vivo). **d**, Immunoblot of HA, TOM20, LDHB, MPC1, β-actin, Calreticulin, and LC3B in *MITO-Tag*^*fl/+*^ hearts, and mitochondrial fractions from *Mct1*^*fl/fl*^, and *Mct1*^*iCKO*^ mice. **e**, Immunoblot from isolated mitochondria used in alamethicin LDH assays. **f**, Relative abundance (fold change) of M + 3 pyruvate in mitochondria from *Mct1*^*fl/fl*^, *Mct1*^*iCKO*^, and *Mpc1*^*iCKO*^ treated with [U-^13^C]pyruvate. **g**, Relative abundance (fold change, FC) of M + 3 lactate in mitochondria from treated with [U-^13^C]lactate, and labeling fraction of downstream metabolites, (**h**) M + 3 pyruvate, (**i**) M + 2 citrate, (**j**) M + 2 malate (**k**) and M + 2 fumarate (*n* = *3 per genotype)*. **l**, Native blue stain gel of mitochondria isolated from *Mct1*^*fl/fl*^ *(n* = *3)*, and *Mct1*^*iCKO*^ *(n* = *3)* hearts. **m, n**, Oxygen consumption (*J*O_2_) rates of isolated cardiac mitochondria incubated with pyruvate or lactate and treated with DMSO (Veh), or CPI-613 (*n* = *6* hearts for pyruvate, *n* = *8* hearts for lactate). **o**, Citrate M + 2 labeling fraction from isolated cardiac mitochondria incubated with [U-^13^C]lactate and treated with DMSO (Veh) or CPI-613, *n* = *3*. **p**, Normalized NADH ion intensities from isolated mitochondria treated with DMSO (Veh), or CPI-613, *n* = *3*. **q, r**, Basal oxygen consumption rates upon lactate addition to ACMs pre-treated with UK5099 or Vehicle in *Mct1*^*fl/fl*^ or *Mct1*^*iCKO*^ ACMs (Seahorse Assay). All data represent mean ± SEM. Significance determined by unpaired two-tailed t tests (c, m-r), or one-way ANOVA with Dunnett’s multiple comparison test (f-k). ns, not significant (p > 0.05). Unmarked comparisons are ns. Source Data

We next tested whether MCT1 was required for the mitochondrial uptake of lactate using [2-^2^H]lactate tracing. During LDH-mediated oxidation, the deuterium at C2 in [2-^2^H]lactate is transferred to NAD^+^, generating NAD^2^H (Fig. 3d). The presence of ^2^H-lactate in the mitochondria signifies that lactate entered the organelle before conversion to pyruvate^29^. However, because mitochondrial isolation from a mouse heart is slow compared with global LDH activity, this approach is unsuitable for in vivo studies. To overcome this limitation, we again employed our Langendorff ex vivo heart system in which we perfused [2-^2^H]lactate into *Mct1*^*iCKO*^ hearts from mice also expressing a cardiomyocyte-specific mitochondria-localized affinity tag (MITO-Tag)^30^ (Extended Data Fig. 7a). We developed a protocol for the rapid isolation of cardiac mitochondria and quenching of lactate metabolism using inhibitor cocktails (Fig. 3e and Extended Data Fig. 7b). In perfused hearts, heart rate and whole-heart ^2^H-lactate labelling were similar across genotypes (Fig. 3f and Extended Data Fig. 7c). However, in the mitochondrial fraction, ^2^H-lactate was absent from *Mct1*^*iCKO*^ samples (Fig. 3f and Extended Data Fig. 7d).

Proteinase K and immunoblot assays identified LDHB within the mitochondrial matrix (Fig. 3b,c). We quantified total matrix LDH activity by performing in vitro LDH assays on purified cardiac mitochondria with and without permeabilization and proteinase K treatment (Fig. 3g). Alamethicin treatment permeabilizes both mitochondrial membranes to small molecule transit (for example, NAD^+^ and ATP) while retaining normal matrix protein localization^31^. Cardiac LDH activity was both highly dependent on permeabilization and insensitive to proteinase K treatment, consistent with matrix localization (Fig. 3g,h and Extended Data Fig. 7e). Liver and kidney mitochondrial LDH activity showed different sensitivities to permeabilization and proteinase K treatment.

## MCT1 enables mitochondrial lactate oxidation

After identifying MCT1 in the IMM and LDH activity in mitochondrial preparations, we purified mitochondria from *Mct1*^*fl/fl*^ (control), *Mct1*^*iCKO*^ and *Mpc1*^*iCKO*^ hearts and characterized their respiration and metabolism on different substrates. Mitochondria from control and *Mct1*^*iCKO*^ hearts respired equivalently on pyruvate, but *Mpc1*^*iCKO*^ mitochondria had reduced oxygen consumption rates (*J*O_2_), impaired ^13^C-citrate production and suppressed pyruvate uptake (Fig. 3i,j and Extended Data Fig. 7f). Conversely, respiration on lactate was similar in both control and *Mpc1*^*iCKO*^ mitochondria but was reduced in mitochondria from *Mct1*^*iCKO*^ hearts (Fig. 3k). [U-^13^C]lactate import and oxidation into TCA cycle metabolites was attenuated in *Mct1*^*iCKO*^ mitochondria but unchanged in mitochondria isolated from the hearts of mice lacking the lactate exporter MCT4 (encoded by *Slc16a3*)^16^ (Fig. 3l and Extended Data Fig. 7g–k). Mitochondria from *Mct1*^*iCKO*^ mice showed no gross defect in mitochondrial electron transport chain complex or supercomplex assembly (Extended Data Fig. 7l). Acute treatment of mitochondria isolated from control mice with an MCT1 inhibitor (7ACC2) or a pan-LDH inhibitor (GSK 2837808A) inhibited respiration on lactate (Fig. 3m). Finally, we treated purified mitochondria from non-failing donor hearts with [U-^13^C]lactate and observed impaired import and oxidation to pyruvate and citrate when treated with an MCT1 inhibitor but not with an MPC inhibitor (Fig. 3n).

The oxidation of one lactate molecule within the mitochondria by LDH to produce pyruvate generates one equivalent of NADH that can transfer electrons directly into the electron transport chain. To test whether this LDH activity could generate sufficient NADH for respiration, we inhibited the TCA cycle with the pyruvate- and α-ketoglutarate dehydrogenase inhibitor CPI-613^32^. As expected, mitochondrial respiration on pyruvate was sharply reduced by CPI-613 treatment, however lactate respiration was unaltered despite M+2 citrate production from ^13^C-lactate being suppressed (Extended Data Fig. 7m–o). NADH levels were unchanged (Extended Data Fig. 7p). Total oxygen consumption on lactate was consistently lower than on pyruvate under minimal assay conditions (buffer, mitochondria, substrate, malate and ADP). Because lactate is converted to pyruvate by LDH, equivalent respiration would be expected if oxidation proceeded identically, suggesting that lactate oxidation may be incomplete. We hypothesized that in the absence of exogenous pyruvate, lactate oxidation may be impaired through pyruvate efflux via the MPC. Pre-treatment of ACMs with UK5099, a potent MPC inhibitor, enhanced lactate-supported respiration (Extended Data Fig. 7q,r). Collectively, these data support a model in which mitochondrial LDH-dependent lactate metabolism supports respiratory flux.

## Loss of MCT1 accelerates heart failure

**Fig. 4:**
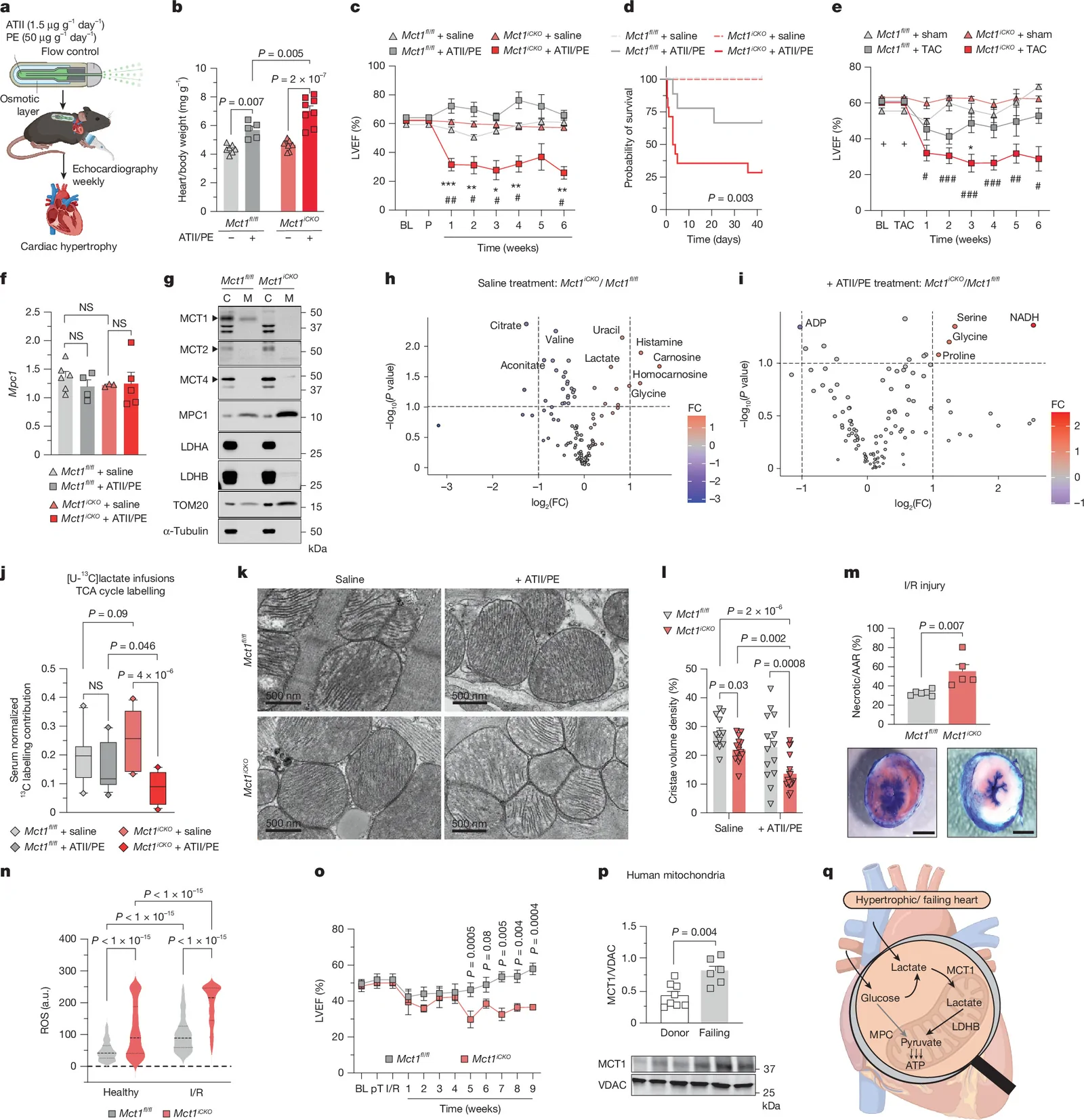
MCT1 is necessary to preserve cardiac ejection fraction upon injury. **a**, ATII/PE dosing in mice. Created in BioRender; Visker, J. https://BioRender.com/4xerzxv (2026). **b**,**c**, Heart weight to body weight ratio (**b**) and LVEF (**c**) after 42 days of ATII/PE (*Mct1*^*fl/fl*^ saline *n* = 7, *Mct1*^*fl/fl*^ ATII/PE *n* = 5, *Mct1*^*iCKO*^ saline *n* = 8, *Mct1*^*iCKO*^ ATII/PE *n* = 8). *P < 0.05, **P < 0.01, comparing *Mct1*^*fl/fl*^ ATII/PE versus *Mct1*^*iCKO*^ ATII/PE. ^#^*P* < 0.05, ^##^*P* < 0.01, comparing *Mct1*^*iCKO*^ ATII/PE versus *Mct1*^*iCKO*^ saline (Methods); *P* values are listed in Supplementary Table 1. BL, baseline. P, pump implant. **d**, Survival after ATII/PE treatment. Significance determined by Gehen–Breslow–Wilcoxon test. **e**, LVEF of mice subjected to TAC (*Mct1*^*fl/fl*^ sham *n* = 5, *Mct1*^*fl/fl*^ TAC *n* = 9, *Mct1*^*iCKO*^ sham *n* = 4, *Mct1*^*iCKO*^ TAC *n* = 10). *P < 0.05, **P < 0.01 comparing *Mct1*^*fl/fl*^ TAC versus *Mct1*^*iCKO*^ TAC. ^#^*P* < 0.05, ^##^*P* < 0.01, ^###^*P* < 0.001, comparing *Mct1*^*iCKO*^ TAC versus *Mct1*^*iCKO*^ sham. †*P* < 0.05, comparing *Mct1*^*fl/fl*^ sham versus *Mct1*^*iCKO*^ sham. *P* values are listed in Supplementary Table 2. **f**, Quantification of *Mpc1* transcripts from treated hearts (*Mct1*^*fl/fl*^ saline *n* = 6, *Mct1*^*fl/fl*^ ATII/PE *n* = 4, *Mct1*^*iCKO*^ saline *n* = 3, *Mct1*^*iCKO*^ ATII/PE *n* = 5). **g**, Immunoblots of fractionated, treated hearts. **h**, Metabolites from saline-treated hearts (*Mct1*^*fl/fl*^ *n* = 7, *Mct1*^*iCKO*^ *n* = 4). *P* values from uncorrected Student’s *t*-test. FC, fold change. **i**, Metabolites from ATII/PE-treated hearts (*Mct1*^*fl/fl*^ *n* = 3, *Mct1*^*iCKO*^ *n* = 5). *P* values from uncorrected Student’s *t*-test. **j**, TCA cycle labelling after in vivo [U-^13^C]lactate infusion in anaesthetized mice (*Mct1*^*fl/fl*^ saline *n* = 6, *Mct1*^*fl/fl*^ ATII/PE *n* = 4, *Mct1*^*iCKO*^ saline *n* = 4,* Mct1*^*iCKO*^ ATII/PE *n* = 6). Boxes represent 25th to 75th percentiles and whiskers extend to 10th and 90th percentiles. **k**, Transmission electron microscopy of ATII/PE-treated *Mct1*^*iCKO*^ a*n*d *Mct1*^*fl/fl*^ hearts. **l**, Cristae volume density (*n* = 3 mice per group; 12–17 images per mouse). **m**, Evans Blue and triphenyltetrazolium chloride staining following in vivo ischemia–reperfusion (I/R) injury (*n* = 5). Scale bars, 2 mm. **n**, Mitosox ROS reporter in ACMs from indicated hearts subjected to ischemia–reperfusion (>100 cells per group). a.u., arbitrary units. **o**, LVEF of mice subjected to ischemia–reperfusion injury (*Mct1*^*fl/fl*^ *n* = 11, *Mct1*^*iCKO*^ *n* = 9). **p**, MCT1 bands from human cardiac mitochondria (donor *n* = 8, heart failure *n* = 6). **q**, Our model of MCT1-dependent lactate metabolism. Created in BioRender; Napoli, G. https://BioRender.com/kl2xa6y (2026). Data are mean ± s.e.m. *P* values from two-way ANOVA (**b**,**f**,**j**,**l**,**n**), unpaired two-tailed *t*-tests with pooled variance (**o**) or unpaired two-tailed *t*-tests (**m**,**p**). Source Data

**Extended Data Fig. 8:**
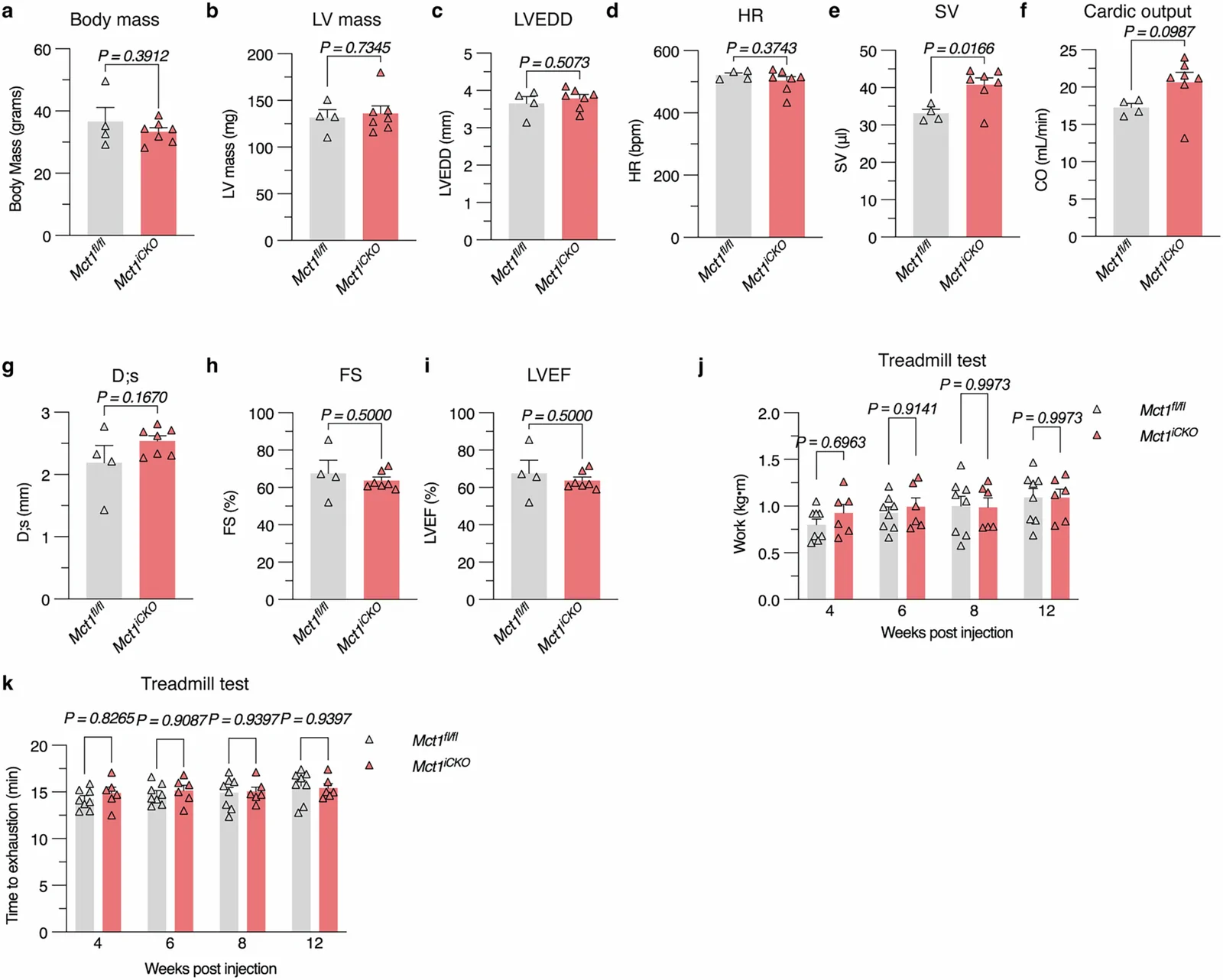
Normal cardiac function and exercise tolerance in 1-year old *Mct1*^*iCKO*^. Echocardiography was performed on mice (*Mct1*^*fl/fl*^ *n* = *4*, and *Mct1*^*iCKO*^ *n* = *7*) older than 11 months of age. **a**, Body mass (g: grams). **b**, absolute left ventricular mass (LV mass: mg). **c**, Left ventricular end diastolic diameter (LVEDD: mm). **d**, Heart rate (HR: bpm). **e**, Stroke volume (SV: μL). **f**, Cardiac output (CO: mL/min). **g**, Left ventricular end systolic diameter (D;s: mm). **h**, Fractional shortening (FS: %). **i**, Left ventricular ejection fraction (LVEF: %). **j** and **k**, Mice were subjected to exercise tolerance tests on a small rodent treadmill and work (kg/m) and time to exhaustion (min) was calculated at 4 w.p.i, 6 w.p.i, 8 w.p.i, and 12 w.p.i. (*Mct1*^*fl/fl*^ *n* = *8*, and *Mct1*^*iCKO*^ *n* = *6*). All data represent mean ± SEM. Significance determined by unpaired two-tailed t tests (a-i) and multiple comparisons corrected (Holm-Sídák method) unpaired two-tailed t tests (j,k). ns, not significant, (p > 0.05). Unmarked comparisons are ns. Source Data

**Extended Data Fig. 9:**
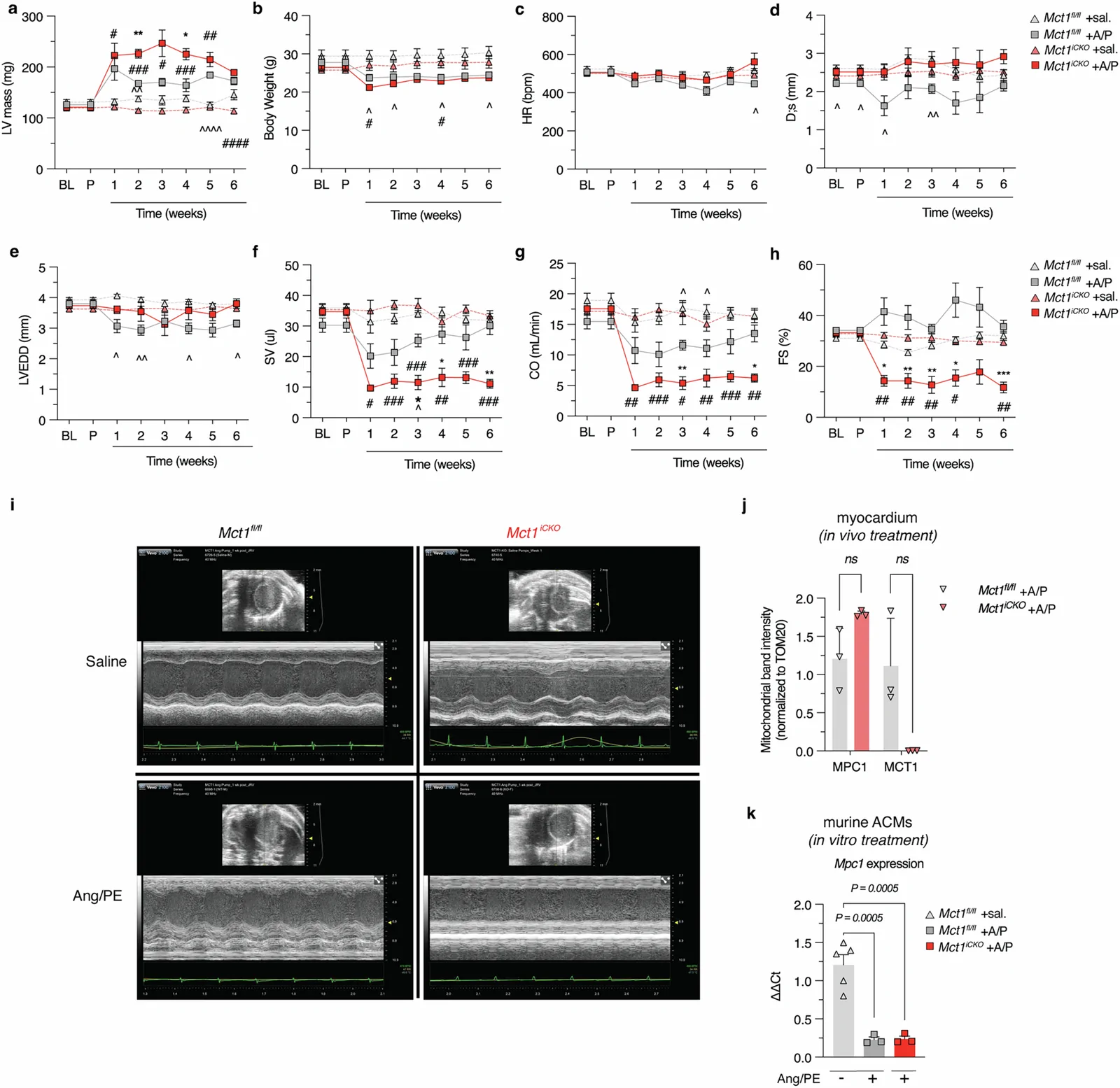
MCT1 deficient hearts have impaired cardiac function upon ANG/PE treatment. Mice (*Mct1*^*fl/fl*^, and *Mct1*^*iCKO*^) were treated with angiotensin II (Ang; 1.5 μg/g/day) and phenylephrine (PE; 50 μg/g/day), or normal saline (0.9%, sterile) by osmotic minipump for six weeks (42 days) (*Mct1*^*fl/fl*^ saline *n* = *7*, *Mct1*^*fl/fl*^ Ang/PE *n* = *8*, *Mct1*^*iCKO*^ saline *n* = *4*, *Mct1*^*iCKO*^ Ang/PE *n* = *10*). Cardiac function was assessed with weekly echocardiography. **a**, Absolute left ventricular mass (LV mass: mg). **b**, Body weight (g). **c**, Heart rate (HR: bpm). **d**, Left ventricular end systolic diameter (D;s: mm). **e**, Left ventricular end diastolic diameter (LVEDD: mm). **f**, Stroke volume (SV: μL). **g**, Cardiac output (CO: mL/min). **h**, Fractional shortening (FS: %). **i**, Representative echocardiography images for *Mct1*^*fl/fl*^, and *Mct1*^*iCKO*^ treated with either saline or Ang/PE. **j**, Quantification of MCT1 and MPC1 protein levels from immunoblots performed on isolated mitochondria from *Mct1*^*fl/fl*^, and *Mct1*^*iCKO*^ myocardium treated with Ang/PE (*n* = *3* hearts). **k**, Quantification of *Mpc1* transcript levels from Ang/PE treated ACMs (*Mct1*^*fl/fl*^ saline *n* = 5, *Mct1*^*fl/fl*^ Ang/PE *n* = 3, *Mct1*^*iCKO*^ Ang/PE *n* = 3). All data represent mean ± SEM. For all echocardiography measurements (a-h), a mixed-effects model (repeated measures ANOVA) with the Geisser-Greenhouse correction was used. If significant (p < 0.05), then a Tukey’s multiple comparison test was applied with individual variances computed for each comparison. ^=p < 0.05, ^^=p < 0.01 comparing *Mct1*^*fl/fl*^ Ang/PE vs. *Mct1*^*fl/fl*^ saline. *=p < 0.05, **=p < 0.01 comparing *Mct1*^*fl/fl*^ Ang/PE vs. *Mct1*^*iCKO*^ Ang/PE. ^#^=p < 0.05, ^##^=p < 0.01, ^###^=p < 0.001 comparing *Mct1*^*iCKO*^ Ang/PE vs. *Mct1*^*iCKO*^ saline. Exact p values are listed in Supplementary Table 1. Significance determined multiple comparisons corrected (Holm-Sídák method) unpaired two-tailed t tests or by one-way ANOVA with Dunnett’s multiple comparison test (k). ns, not significant (p > 0.05). Source Data

**Extended Data Fig. 10:**
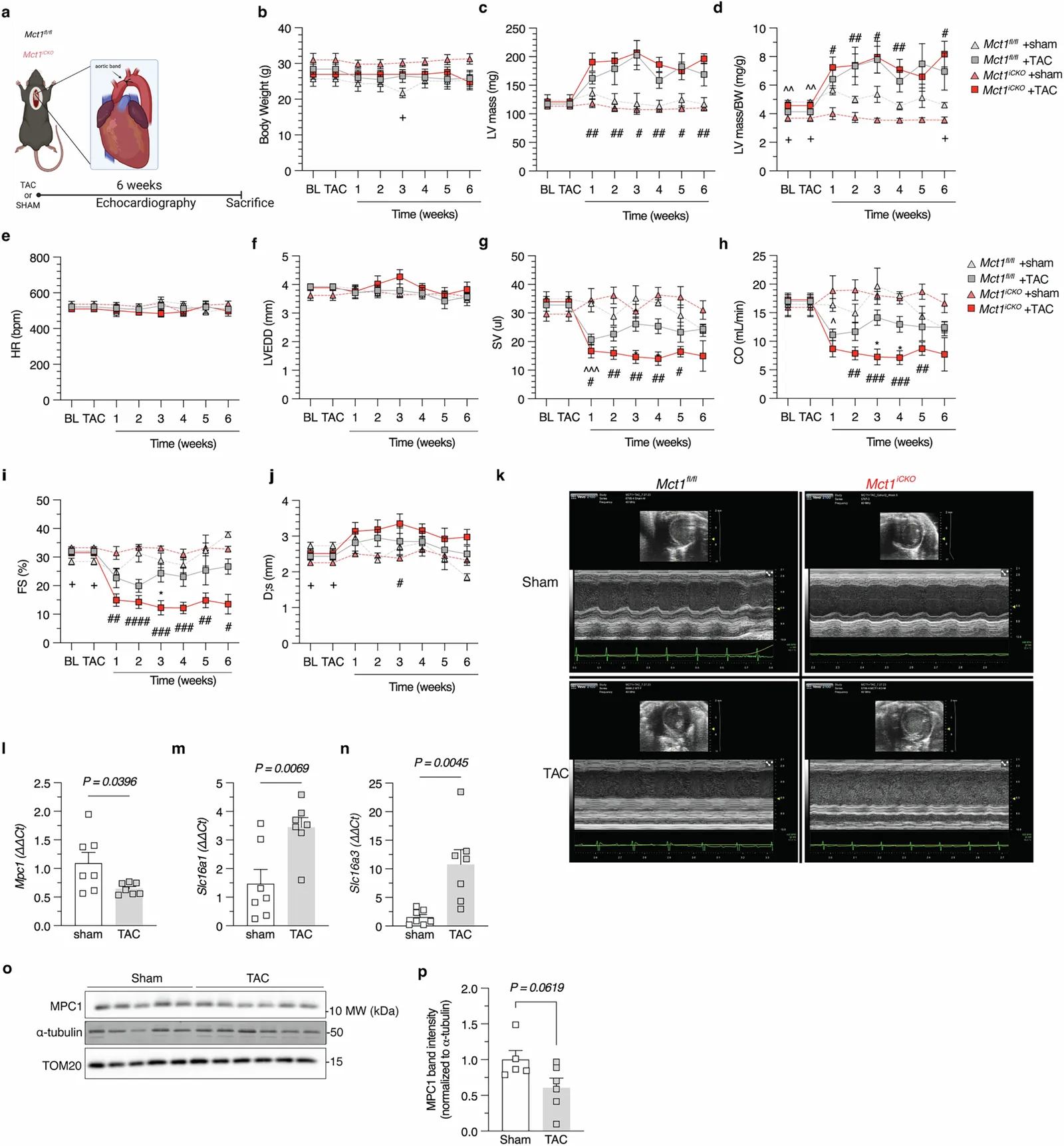
MCT1 deficient hearts have impaired cardiac function upon TAC-induced pressure overload. Mice (*Mct1*^*fl/fl*^, and *Mct1*^*iCKO*^) were subjected to transverse aortic constriction (TAC), or a sham surgery (*Mct1*^*fl/fl*^ sham *n* = *5*, *Mct1*^*fl/fl*^ TAC *n* = *9*, *Mct1*^*iCKO*^ sham *n* = *4*, *Mct1*^*iCKO*^ TAC *n* = *10*). Cardiac function was assessed by weekly echocardiography. **a**, Schematic of experimental design. Created in BioRender; Visker, J. https://BioRender.com/09lonbc (2026). **b**, Body weight (g). **c**, absolute left ventricular mass (LV mass: mg). **d**, Standardized LV mass to body weight (LV mass/BW: mg/g). **e**, Heart rate (HR: bpm). **f**, Left ventricular end diastolic diameter (LVEDD: mm). **g**, Stroke volume (SV: μL). **h**, Cardiac output (CO: mL/min). **i**, Fractional shortening (FS: %). **j**, Left ventricular end systolic diameter (D;s: mm). **k**, Representative echocardiography images for *Mct1*^*fl/fl*^, and *Mct1*^*iCKO*^ that underwent TAC or sham surgery. **l, m, n**, Quantified transcript levels of *Mpc1*, *Slc16a1* and *Slc16a3* from control (sham) and TAC hearts from C57Bl/6 animals (sham *n* = *7*, TAC *n* = *7*). **o**. Immunoblots of MPC1, TOM20 and α-tubulin from whole-heart extracts (*n* = *5-6* hearts). **p**. Quantification of MPC1 immunoblots from o. All data represent mean ± SEM. Significance determined by a mixed-effects model (repeated measures ANOVA) with the Geisser-Greenhouse correction. If significant (p < 0.05), then a Tukey’s multiple comparison test was applied with individual variances computed for each comparison (b-j). ^=p < 0.05, ^^=p < 0.01 comparing *Mct1*^*fl/fl*^ TAC vs. *Mct1*^*fl/fl*^ Sham. *=p < 0.05, **=p < 0.01 comparing *Mct1*^*fl/fl*^ TAC vs. *Mct1*^*iCKO*^ TAC. ^#^=p < 0.05, ^##^=p < 0.01, ^###^=p < 0.001 comparing *Mct1*^*iCKO*^ TAC vs. *Mct1*^*iCKO*^ Sham. ^+^=p < 0.05, comparing *Mct1*^*fl/fl*^ Sham vs. *Mct1*^*iCKO*^ Sham. Exact p values are listed in Supplementary Table 2. Significance determined by unpaired two-tailed t tests (l-n, p). ns, not significant (p > 0.05). Source Data

Having established that MCT1 is required for lactate oxidation, we explored whether loss of this biochemical functionality affects cardiac physiology. *Mct1*^*iCKO*^ mice did not show evidence of structural or functional cardiac abnormalities by 1 year of age as measured by echocardiography (Extended Data Fig. 8a–i). Consistent with this, *Mct1*^*iCKO*^ mice exhibited unchanged maximal exercise capacity over time (Extended Data Fig. 8j,k). We hypothesized that the absence of a phenotype was a consequence of the functional redundancy between MCT1 and MPC in cardiomyocytes. Therefore, we turned to a neurohormonal agonist-induced model of cardiac hypertrophy that we previously reported to decrease *Mpc1* activity^16^. We surgically implanted mice with osmotic minipumps delivering angiotensin II and phenylephrine (ATII/PE) to induce cardiac hypertrophy (Fig. 4a). After 6 weeks, all treated mice developed cardiac hypertrophy, which was more pronounced in *Mct1*^*iCKO*^ mice (Fig. 4b and Extended Data Fig. 9a). Body weight, heart rate, left ventricular end systolic diameter and left ventricular end diastolic diameter were not significantly different between genotypes and were only modestly affected by treatment (Extended Data Fig. 9b–e). By contrast, systolic function as measured by stroke volume was reduced in *Mct1*^*iCKO*^ mice, leading to lower cardiac output, fractional shortening and impaired LVEF (Fig. 4c and Extended Data Fig. 9f–i). Consequently, ATII/PE-treated *Mct1*^*iCKO*^ mice exhibited increased mortality (Fig. 4d). Similarly, loss of MCT1 accelerated the development of heart failure in mice when cardiac hypertrophy was induced by pressure overload caused by transverse aortic constriction (TAC) (Fig. 4e and Extended Data Fig. 10a–k).

To understand the accelerated development of heart failure in *Mct1*^*iCKO*^ mice, we performed targeted gene expression analysis on ATII/PE and TAC-treated hearts. Neither loss of *Mct1* nor ATII/PE treatment led to a reduction in *Mpc1* gene or protein expression in myocardium extracts (Fig. 4f,g and Extended Data Fig. 9j). However, in purified adult cardiomyocytes treated with ATII/PE we observed a reduction in *Mpc1* mRNA levels (Extended Data Fig. 9k). By contrast, TAC treatment led to a reduction in myocardial *Mpc1* transcript levels and a trend towards reduced protein as well (Extended Data Fig. 10l,o,p). *Slc16a1* and *Slc16a3* transcripts were increased in control mice (Extended Data Fig. 10m,n). These data suggest that in some models of heart failure, MPC1 is suppressed and MCT1 may compensate.

## MCT1 loss impairs mitochondrial function

**Extended Data Fig. 11:**
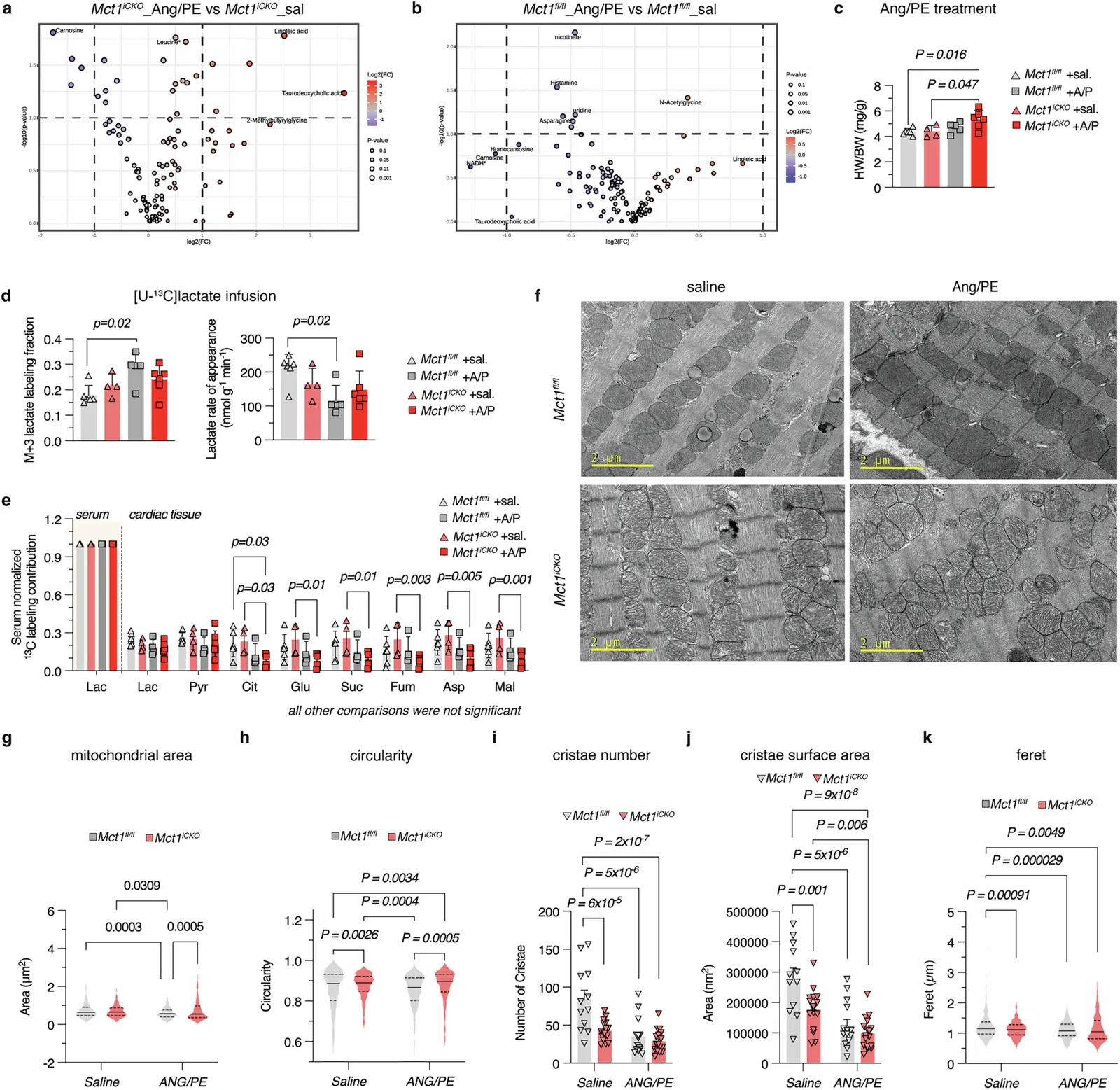
Metabolic and mitochondrial changes upon loss of MCT1. **a, b**, Volcano plots showing statistically significantly differentially expressed polar metabolites from Ang/PE and saline treated myocardium from *Mct1*^*iCKO*^ and *Mct1*^*fl/fl*^ animals respectively (*Mct1*^*fl/fl*^ saline *n* = *7*, *Mct1*^*fl/fl*^ Ang/PE *n* = *3*, *Mct1*^*iCKO*^ saline *n* = *4*, *Mct1*^*iCKO*^ Ang/PE *n* = *5*, p values from uncorrected Student’s t test). **c**, Heart weight normalized to body weight (mg/g) from *Mct1*^*fl/fl*^ and *Mct1*^*iCKO*^ animals treated with either saline or Ang/PE for 1 week and used in experiment h (*Mct1*^*fl/fl*^ saline *n* = *6*, *Mct1*^*fl/fl*^ Ang/PE *n* = *5*, *Mct1*^*iCKO*^ saline *n* = *4*, *Mct1*^*iCKO*^ Ang/PE *n* = *6*). **d**, Serum lactate labeling fraction and rate of appearance (R_a_) (*Mct1*^*fl/fl*^ saline *n* = *6*, *Mct1*^*fl/fl*^ Ang/PE *n* = *5*, *Mct1*^*iCKO*^ saline *n* = *4*, *Mct1*^*iCKO*^ Ang/PE *n* = *6*). **e**, Heart ^13^C labeling of lactate, pyruvate and TCA cycle metabolites from tail vein [U-^13^C]lactate infusions in *Mct1*^*fl/fl*^, or *Mct1*^*iCKO*^ mice treated with either saline or Ang/PE (*Mct1*^*fl/fl*^ saline *n* = *6*, *Mct1*^*fl/fl*^ Ang/PE *n* = *4*, *Mct1*^*iCKO*^ saline *n* = *4*, *Mct1*^*iCKO*^ Ang/PE *n* = *5*). All unmarked comparisons are not significant. **f**, Representative large-scale images from transmitted electron microscopy (TEM) conducted on saline or Ang/PE treated *Mct1*^*iCKO*^ and *Mct1*^*fl/fl*^ hearts. Representative images from *n* = *4* hearts and multiple images from each heart. **g-k**, Quantified mitochondrial structural parameters from TEM images showing mitochondrial area (**g**), circularity (**h**), cristae number (**i**), cristae surface area (**j**), and Feret (**k**) in saline or Ang/PE treated *Mct1*^*iCKO*^ and *Mct1*^*fl/fl*^ hearts. All data represent mean ± SEM. Significance determined by one-way ANOVA with Dunnett’s multiple comparison test (c,d) or two-way ANOVA with Tukey’s multiple comparison correction (e-k). ns, not significant (p > 0.05). Unmarked comparisons are ns. Source Data

To reconcile the severity of our cardiac phenotype across models with conflicting gene expression data, we performed metabolomic analysis on ATII/PE-treated myocardium. There were significant genotype-specific differences in metabolite abundances between *Mct1*^*iCKO*^ and *Mct1*^*fl/fl*^ hearts upon ATII/PE treatment (Fig. 4h,i and Extended Data Fig. 11a,b and Supplementary Table 4). Saline-treated *Mct1*^*iCKO*^ hearts had increased levels of lactate, histamine, carnosine and glycine and reduced levels of citrate and isocitrate compared with *Mct1*^*fl/fl*^ hearts. Upon ATII/PE treatment, metabolite changes in *Mct1*^*iCKO*^ hearts were of greater magnitude and NADH was the most upregulated metabolite, indicative of an impairment in oxidative phosphorylation (Fig. 4i). Consistent with a critical mitochondrial defect, all purine nucleotides were downregulated, with ADP being the most statistically significantly reduced metabolite in failing *Mct1*^*iCKO*^ hearts (Fig. 4i). Metabolome differences were much less pronounced upon ATII/PE treatment in control hearts (Extended Data Fig. 11b).

We next performed [U-^13^C]lactate infusions on mice treated with ATII/PE to understand whether the observed steady-state metabolic changes between *Mct1*^*iCKO*^ hearts before and after ATII/PE treatment reflected changes in cardiac substrate metabolism. Mice were treated with ATII/PE for 1 week prior to anaesthetized tail vein infusions to reduce procedure-induced mortality. ATII/PE treatment led to a rapid increase in the ratio of heart weight to body weight in *Mct1*^*iCKO*^ mice (Extended Data Fig. 11c). Lactate turnover was systemically lower under anaesthesia in all conditions compared with awake mice and was further reduced in *Mct1*^*fl/fl*^ mice subjected to ATII/PE (Extended Data Fig. 11d). Treatment did not affect the contribution of lactate to TCA labelling in *Mct1*^*fl/fl*^ mice (Fig. 4j and Extended Data Fig. 11e). By contrast, the contribution of lactate to the TCA cycle (malate, succinate and glutamate) was attenuated in hearts from *Mct1*^*iCKO*^-treated mice with ATII/PE compared with saline-treated controls and *Mct1*^*fl/fl*^ ATII/PE mice (Fig. 4j and Extended Data Fig. 11e).

We further investigated the nature of the impaired mitochondrial function in *Mct1*^*iCKO*^ mice using electron microscopy (Fig. 4k and Extended Data Fig. 11f). Total mitochondria area was not different between saline conditions and increased upon ATII/PE treatment in the *Mct1*^*iCKO*^ group (Extended Data Fig. 11g). Relatedly, the overall mitochondrial morphology differed significantly between saline and ATII/PE samples (Extended Data Fig. 11h–k). Cristae volume density was reduced in *Mct1*^*iCKO*^ compared with *Mct1*^*fl/fl*^ mitochondria and this difference was more pronounced under ATII/PE treatment (Fig. 4l). The high degree of circularity and reduced cristae volume density of mitochondria has been shown to correlate with oxidative stress^33,34^ and may indicate a role for MCT1 in cristae maintenance and contribute to the global impairment in oxidation seen in *Mct1*^*iCKO*^ with ATII/PE treatment.

## MCT1 is protective for ischemic injury

**Extended Data Fig. 12:**
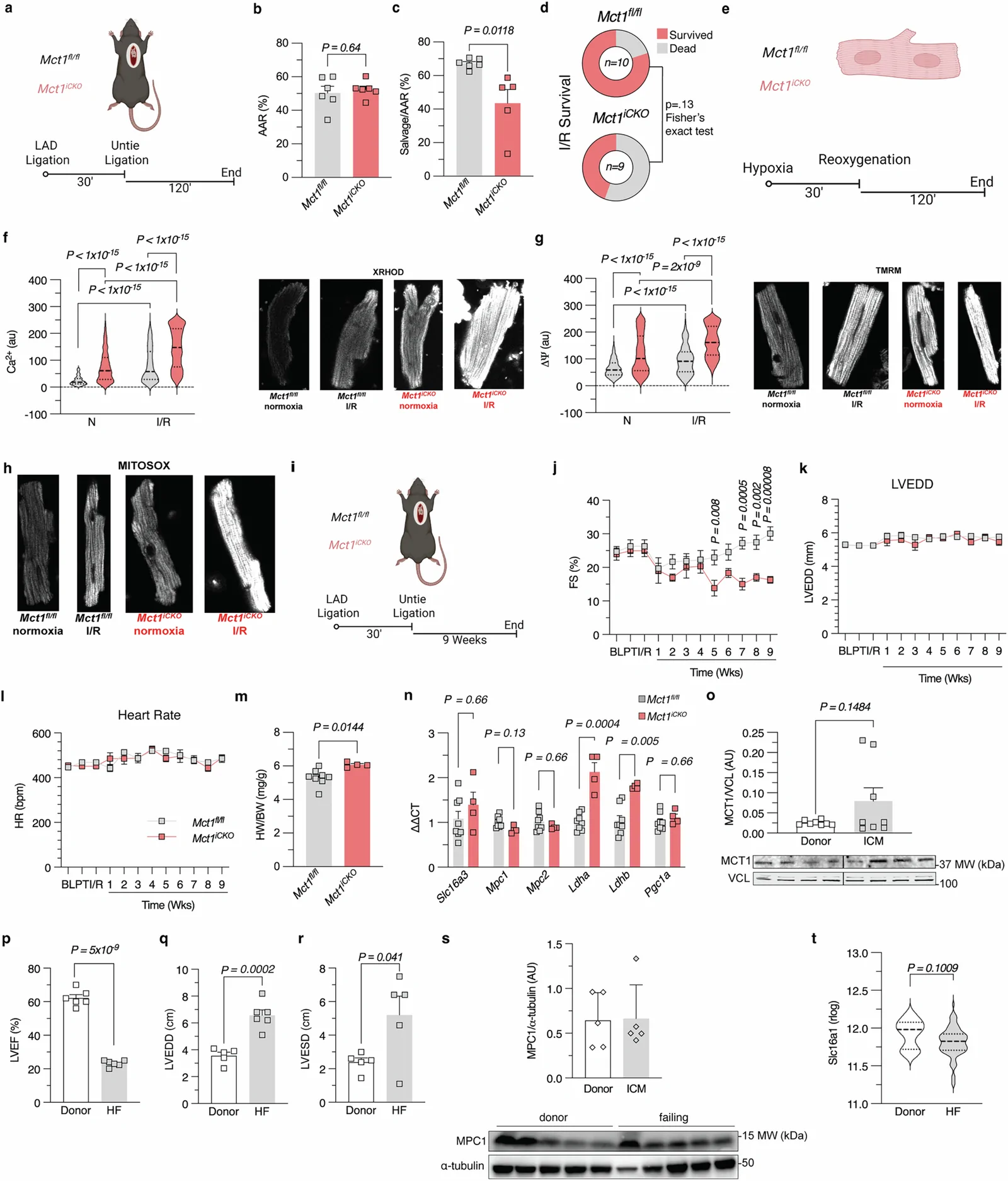
Mct1 deletion leads to enhanced cell death, increased hypertrophy, and impairs mitochondrial function following hypoxia and ischemia. **a**, Schematic of in vivo acute ischemia-reperfusion injury (I/R) in *Mct1*^*fl/fl*^, and *Mct1*^*iCKO*^ mice. Created in BioRender; Visker, J. https://BioRender.com/1xyw964 (2026). **b**, Area at risk (AAR: %) after I/R injury (*n* = *6*). **c**, Myocardial salvage standardized to the AAR (*n* = *6*). **d**, Survival rates of the mice subjected to acute I/R injury (*Mct1*^*fl/fl*^ *n* = *10*, *Mct1*^*iCKO*^ *n* = *13*). **e**, Schematic of in vitro hypoxia and reoxygenation experiment using ACMs from *Mct1*^*fl/fl*^, and *Mct1*^*iCKO*^ hearts. **f-h**, Mitochondrial specific reporters for calcium (Ca^2+^: XRhod), membrane potential (Δψ: TMRM), and reactive oxygen species (ROS: mitosox), respectively in ACMs isolated from *Mct1*^*fl/fl*^, and *Mct1*^*iCKO*^ hearts. Images and quantification ( > 100 cells/ group) shown for each dye. Mitosox quantification is shown as Fig. 4n. **i**, Diagram representing in vivo chronic ischemia-reperfusion injury (I/R) in *Mct1*^*fl/fl*^, and *Mct1*^*iCKO*^ mice. Mice were subjected to cardiac injury and surviving mice allowed to recover (*Mct1*^*fl/fl*^ *n* = *11*, and *Mct1*^*iCKO*^ *n* = *9*). Echocardiograms were performed weekly for nine weeks following I/R injury. Created in BioRender; Visker, J. https://BioRender.com/1xyw964 (2026). **j**, Fractional shortening (FS: %). **k**, Left ventricular end diastolic diameter (LVEDD: mm). **l**, Heart rate (HR: beats per minute; bpm). **m**, Heart weight standardized to body weight (mg/h) in *Mct1*^*fl/fl*^, and *Mct1*^*iCKO*^ mice at the end of nine-weeks following I/R injury. **n**, Quantified transcript levels of *Slc16a1*, *Mpc1*, *Mpc2, Ldha, Ldhb, Pgc1α* from *Mct1*^*fl/fl*^ (*n* = *9*) and *Mct1*^*iCKO*^ animals (*n* = *4*). **o**, Relative MCT1 abundance normalized to Vinculin (VCL) (AU: arbitrary units) in non-failing donor heart samples compared to samples acquired from advanced heart failure (HF) patients undergoing transplant due to ischemic cardiomyopathy (ICM) (donors *n* = *8*, ICM *n* = *8*). **p-r**, Echocardiography comparing non-failing donor hearts to advanced HF patients LVEF (%), LVEDD (cm), and LVESD (cm). **s**, Immunoblot of MPC1 from frozen patient myocardium samples matching a. **t**, Standardized r-log values from transcriptomics (RNA sequencing) comparing non-failing donor heart samples to samples acquired from advanced HF patients at the time of left ventricular assist device (LVAD) implant. Data are plotted as mean ± SEM. Significance determined by unpaired two-tailed t tests (b,c,m,o-t), multiple comparisons corrected (Holm-Sídák method) unpaired two-tailed t tests (l,n), two-way ANOVA with Tukey’s multiple comparison correction (f,g). ns, not significant (p > 0.05). Unmarked comparisons are ns. Source Data

Increased MCT1 expression has been reported in myocardium during ischemia–reperfusion injury^35,36^. To study the consequences of MCT1 loss during ischemia–reperfusion injury and to explore the connection between MCT1 and susceptibility to mitochondrial oxidative stress, we studied *Mct1*^*iCKO*^ mice subjected to ischemia–reperfusion^37^ (Extended Data Fig. 12a). *Mct1*^*iCKO*^ mice had myocardial areas at risk for infarction of similar size, but larger necrotic areas and a trend towards higher mortality compared with control littermates (Fig. 4m and Extended Data Fig. 12b–d). To understand whether these effects were correlated with intrinsic defects in *Mct1*^*iCKO*^ cardiomyocyte function upon hypoxia we utilized an in vitro hypoxia–reoxygenation setup that models ischemia–reperfusion injury^37^ (Extended Data Fig. 12e). At baseline, *Mct1*^*iCKO*^ ACMs had increased mitochondrial membrane potential (Δ*Ψ*), higher calcium (Ca^2+^) levels, and elevated reactive oxygen species (ROS) that were further increased after hypoxia–reoxygenation (Fig. 4n and Extended Data Fig. 12f–h). The MCT1-dependent changes in ROS are congruent with our mitochondrial ultrastructure data and suggest a role for MCT1 in the structure and function of mitochondria which become pathogenic upon cellular damage and energetic stress.

Collectively, our data indicate a key role for cardiac MCT1 in metabolic adaptation to models of both acute and chronic cardiac stress. To assess whether MCT1 was important for recovery from injury and cardiac remodelling, we allowed *Mct1*^*iCKO*^ mice subjected to ischemia–reperfusion injury to recover (Extended Data Fig. 12i). In *Mct1*^*fl/fl*^ and *Mct1*^*iCKO*^ mice, LVEF decreased by a similar amount immediately after the procedure. However, in *Mct1*^*fl/fl*^ mice systolic function returned to baseline by 7 weeks whereas *Mct1*^*iCKO*^ mice showed no recovery of heart function over the duration of the study (Fig. 4o and Extended Data Fig. 12j–m). In the failing hearts of *Mct1*^*iCKO*^ mice, expression of *Lhda* and *Ldhb* transcripts was significantly increased, and *Mpc1* expression was decreased, indicating glucose and lactate metabolic pathway stress that was independent of *Ppargc1a* (encoding PGC1α; expression was unchanged; Extended Data Fig. 12n). Finally, analysis of mitochondria from patients with advanced heart failure with reduced ejection fraction and non-failing donor controls revealed increased mitochondrial MCT1 localization without a statistically significant change in total MCT1 or MPC protein levels (Fig. 4p and Extended Data Fig. 12o–s). Among all patients in our cohort, myocardial *SLC16A1* transcript levels were reduced in heart failure, however the difference was not statistically significant (Extended Data Fig. 12t).

## Discussion

Our findings demonstrate that in the myocardium, glucose-derived carbons can enter the TCA cycle independently of the MPC through a pathway that is dependent on MCT1 (Fig. 2a). Using complementary approaches, we show that MCT1 resides in the IMM and its loss impairs lactate oxidation at the mitochondrial, cellular and tissue level. Loss of MCT1 compromises metabolic adaptation to cardiac stress, leading to structural and functional deterioration and ultimately heart failure. These results support a model in which mitochondrial lactate import enables glucose and lactate carbons to fuel the TCA cycle independent of the MPC while preserving cytosolic redox balance and sustaining glycolysis (Fig. 4q). Our model reconciles the long-standing observation of high systemic lactate fluxes in mammalian physiology^6,7,38^, mitochondrial lactate import independent of the MPC in purified systems^39^ and direct evidence from cellular lactate biosensors showing substantial lactate accumulation within mitochondria^40,41^.

Our data extend and refine the ‘lactate shuttle’ hypothesis developed by physiologists^29,42–46^. The rapid uptake and oxidation of lactate is a hallmark of exercise metabolism^47–49^. However, cytosolic lactate oxidation is inherently constrained by competition with glycolysis for NAD^+^, requiring the invocation of compartmentalized metabolism at the cellular or subcellular level^8,25^. Locating LDHB-driven lactate oxidation within the mitochondrial matrix resolves this conflict. The lower Michaelis constant (*K*_m_) of LDHB for pyruvate compared with LDHA^50^ favours forward lactate oxidation under non-equilibrium cardiac conditions, enhancing metabolic flexibility. In the mitochondria, lactate can shuttle an additional pair of cytosolic electrons into NADH for transfer into the mitochondrial quinone pool to fuel complex I activity. ATP production from lactate depends upon oxygen to serve as the terminal electron acceptor and drive the equilibrium of the reaction towards carbon oxidation. The lactate shuttle hypothesis, however, has been vigorously contested on the basis of conflicting data from isolated mitochondrial systems^41^ and on biochemical grounds^25,26^. Although our in vivo data cannot rigorously separate the dual roles of MCT1 in mitochondrial and cytoplasmic lactate transport, our work based on functional and tracing data from purified mitochondria, permeabilized cells, isolated organs and mice under physiological conditions establishes MCT1 as necessary for mitochondrial lactate oxidation in heart. We note that direct comparisons of in vivo cardiac metabolism between healthy and failing hearts are affected by systemic metabolic perturbations from anaesthesia.

Mitochondrial lactate transport provides crucial redundancy for carbohydrate entry into the mitochondria, enabling cardiomyocytes to maintain glucose oxidation even in the absence of the MPC^17,18^. We show that this redundancy is critical to support the heart’s need for a rapid and flexible energy supply in the face of cardiac stress and position mitochondrial lactate metabolism as a central determinant of cardiac structure, function and resilience.

## Methods

### Human heart samples

Human myocardial tissue samples were acquired from patients through the Utah Cardiac Recovery Program at the University of Utah under an approved IRB protocol (IRB-00030622). All samples were collected with informed consent from patients, organ donors or their guardians. Samples were acquired from consented patients who had chronic advanced heart failure with reduced ejection fraction at the time of heart transplantation. Non-failing donors that were ineligible for heart transplant due to non-cardiac reasons (such as donor–recipient size mismatch or infection) were used as a control. Myocardial tissue was collected in the operating room and immediately frozen in liquid nitrogen and subsequently stored in −80 °C until further use.

### Animal care

All procedures were approved by the Institutional Animal Care and Use Committee (IACUC) of the University of Utah. Mice were housed at 22–23 °C using a 12 h light:12 h dark cycle. Mice were maintained on irradiated chow diet (Teklad Diet 2920x) with ad libitum access to water at all times. Food was only withdrawn during experiments starting at the beginning of the light cycle.

Adult cardiac-specific MPC1-knockout (Mpc1^*fl/fl*^; *αMHC*^*MerCreMer*^) mice were generated as previously described^51^. *Mct1*^*fl/fl*^ mice were generated as previously described^52^. Cardiac-specific Cre recombinase was introduced by crossing mice with *αMHC*^*MerCreMer*^ mice (Jackson Laboratories strain 005657). 8-week-old *Mct1*^*fl/fl*^:αMHC^MerCreMer^^*+/−*^ and *Mct1*^*fl/fl*^*; αMHC*^*MerCreMer−/−*^ littermates were injected intraperitoneally with 40 mg kg^−1^ tamoxifen for 3 consecutive days to yield adult *Mct1*^*iCKO*^ and *Mct1*^*fl/fl*^ (control) littermates.

Adult cardiac-specific MCT4-knockout (Mct4^*fl/fl*^; *αMHC-MerCreMer*) mice were generated as follows. Three embryonic clones carrying *Slc16a3 tm1a* (sencoding MCT4) were obtained from European Conditional Mouse Mutagenesis program/European Mouse Mutant Archive (EUCOMM/EMMA; http://www.infrafrontier.eu/). All clones were from the cell line JM8A3.N1 (background strain C57Bl/6NTac with agouti coat colour distribution). These embryonic stem cell clones were injected into the blastocoel of 3.5-day-old mouse blastocysts (host strain C57Bl/6 *Tyr*^*c-Brd*^). Injected embryos were transferred to the uterine horns of appropriately timed pseudopregnant recipient C57BL/6-*Tyr*^*c-Brd*^ females. The chimeras with highest embryonic stem cell contribution were bred with C57Bl/6J mates to test for germline transmission of the targeted allele. The pups were screened for the presence of germline transmitted tm1a and those positive were further propagated (first allele knockout tm1a, reporter-tagged insertion with conditional potential). Mice carrying the appropriate flox alleles were crossed with *αMHC*^*MerCreMer*^ mice as described above. Experiments on all mice began 4 weeks post-injection (at 12 weeks of age).

### Echocardiographic assessment

Mice were anaesthetized with 1.5% isoflurane (Vet One, NDC 13985-046-60). Echocardiographic images were captured using the Vevo imaging system (VisualSonics) sequentially. Both 2D long-axis and short-axis views were obtained and analysed with Vevo Strain software (v3.1.1). All measurements were conducted using three consecutive cardiac cycles. Limb leads were affixed using conductive cream to facilitate electrocardiogram (ECG) recording.

### Osmotic minipump model

Twelve-week-old C57BL/6 mice were divided into four groups, weighed, and screened before surgery by echocardiography to establish baseline cardiac parameters. Surgical instruments were sterilized using a glass bead sterilizer prior to the surgery and aseptic technique was utilized during the procedure. The osmotic minipumps (Alzet, model 2006) were filled with angiotensin II (1.5 μg g day^−1^) and phenylephrine (50 μg g day^−1^) in 0.9% NaCl at 0.15 μl h^−1^ for 42 days (Model 2006) according to the manufacturer’s instructions. The pumps were surgically implanted subcutaneously on the back through a small incision, under isoflurane (1.5% in O_2_) anaesthesia. After the pump was implanted, and the incision was closed, the mice were placed in a clean, warm cage and monitored for any signs of distress. Next, mice were weighed and screened weekly via echocardiography. After 42 days of treatment with AngII/PE, the mice were euthanized by cervical dislocation and blood and heart tissue were taken for further analysis.

### TAC experiments

Baseline echocardiograms were acquired, and TAC was surgically performed on both male and female *Mct1*^*fl/fl*^ and *Mct1*^*iCKO*^ mice aged 12–14 weeks, as previously described^53,54^. In brief, before surgery, a subcutaneous dose of sustained-release Buprenorphine SR (0.15 mg kg^−1^) was administered for analgesia. Mice were anaesthetized with 2–3% isoflurane in oxygen, delivered via nosecone connected to a VetFlo vaporizer. The aortic arch was visualized and constricted using a titanium clip. The surgical incision was closed using 6-0 sutures in layers. During recovery, regular-release buprenorphine was given as needed, for up to 48 h. Unclipped *Mct1*^*fl/fl*^ and *Mct1*^*iCKO*^ mice (sham) served as controls, undergoing anaesthesia and a small incision without aortic constriction or cardiac damage. Following surgery, mice received weekly echocardiograms for six consecutive weeks to assess cardiac function and structure.

### Ischemia–reperfusion experiments

The experimental protocol was conducted on 20–30 g, 12–14-week-old *Mct1*^*iCKO*^ and *Mct1*^*fl/fl*^ mice, 4 weeks after tamoxifen injection and before the onset of heart failure symptoms. Mice were anaesthetized with 2–3% isoflurane, intubated using a 22 G IV catheter, and ventilated at 100 breaths per min with a small rodent respirator. Mice were placed supine on a warming pad at 38 °C, and chest hair was removed with depilatory cream, which was rinsed off with water or saline to avoid skin irritation. The skin was then prepped with betadine and alcohol. A thoracotomy was performed to access the heart, and a retractor was used to improve visibility. The pericardium was carefully removed, avoiding myocardial damage. Under a dissecting microscope, the left anterior descending coronary artery (LAD) was located, and a 6-0 silk suture was placed under it. A loose double knot was tied with a 2–3 mm diameter loop, through which a 2–3 mm piece of PE-10 tubing was placed. The tubing, pre-soaked in 100% ethanol and rinsed with sterile water, was used to tighten the loop around the artery, achieving coronary occlusion for 30 min. Successful LAD ligation was confirmed by a paler colour on the LV anterior wall. After 30 min, the knot was untied, and the tubing was removed, confirming reperfusion by the return of a red colour to the LV wall, which continued for 2 h.

In acute experiments, the coronary artery was briefly re-occluded after injury, and Evans blue dye was injected into the left atrium to mark the area at risk. Hearts were excised, perfused with saline, weighed and sectioned into 1 mm slices using a vibratome, or separated into ischemic and non-ischemic samples and flash-frozen in liquid nitrogen. Slices were incubated in 1% triphenyltetrazolium chloride at 38 °C for 20 min to stain viable myocardium, then fixed in 10% formalin to distinguish between viable and necrotic tissue. Tissue sections were imaged the next day, and myocardial salvage was quantified using ImageJ by blinded researchers. For chronic experiments, mice underwent 30 min of ischemia, after which the ligature was removed, and the incisions were closed with sutures. Mice were placed in a warm cage, monitored for distress, weighed, and screened weekly via echocardiography.

### Exercise capacity testing

12–14-week-old mice (*Mct1*^*fl/fl*^ and *Mct1*^*iCKO*^) practiced a 5-day treadmill acclimation protocol prior to any maximal exertion. This allowed for familiarity with the procedure, but did not produce any training effect on the mice^55^. In brief, following a day of rest, mice were weighed, and the maximal treadmill test was performed. The test started with a warm-up period of 10 min, a treadmill speed of 10 m min^−1^, and an incline of 10% grade. After this, the speed was increased by 5 m min^−1^ every 2 min until the mouse resisted a physical stimulus (touching the tail or backside of the mouse with a bristle brush) to run for more than 20 s or sat on the back of the non-moving treadmill belt five times in a 30 s period. If either occurred the time was recorded, the test was finished, and the mouse was returned to its cage to recover. Maximal treadmill speed did not exceed 30 m min^−1^ throughout the exercise testing. Total work (in kg m^−1^) and time to exhaustion (in min) were then calculated for each mouse. Blood lactate (in mmol l^−1^) was measured at 10, 20, and 30 m min^−1^, and 10% grade to assess the systemic effects of lactate production in the *Mct1*^*iCKO*^ compared with the *Mct1*^*fl/fl*^ controls.

### Jugular vein catheterization surgery

Sterile mouse jugular vein catheters were purchased from SAI Infusion Technologies and one channel vascular access button from Instech Laboratories. Mice were fasted before surgery, pre-operatively shaved and administered bupivacaine before anaesthesia. A dissection microscope (Leica S9i Stereo) was utilized during the placement of the catheter. Mice were provided standard recovery support and allowed at least 5 days recovery before further use in experiments. Mice with impaired vital signs, activity or reflexes were euthanized and not included in experimental studies.

### Stable isotope infusions

12-week-old surgically catheterized mice were maintained on a normal light cycle (06:00–18:00). On the day of infusion experiment, mice were transferred to new cages without food at 06:00 (beginning of their sleep cycle) and infused for 2–3 h (metabolite-specific) starting at around 12:00 to reach serum steady state based on previously published studies. The infusion setup (Instech Laboratories) included a swivel and tether to allow the mouse to move around the cage freely. Water-soluble isotope-labelled metabolites (Cambridge Isotope Laboratories) were prepared as solutions in sterile normal saline. To make ^13^C-labelled fatty acid solutions, the fatty acids were complexed with bovine serum albumin in a molar ratio 4:1. The infusion rate was set to 0.1 μl min^−1^ g^−1^ for water-soluble metabolites and 0.4 μl min^−1^ g^−1^ for fatty acids. Final infusion solutions and times were 200 mM [U-^13^C]glucose for 3 h, 490 mM [U-^13^C]sodium lactate for 2 h, 50 mM [U-^13^C]3-hydroxybutyrate, 8 mM [U-^13^C]oleic acid or 4 mM [U-^13^C]sodium palmitate all for 3 h. [U-^13^C]glucose infusion with AZD treatment was achieved by dosing mice with 100 mg kg^−1^ AZD or vehicle (0.5% HPMC (hydroxypropyl methylcellulose), 0.1% Polysorbate 80) by oral gavage 6 h prior to infusion start and again at start of infusion. [U-^13^C]glucose was infused using the same parameters as other experiments and mouse blood and tissue harvested as described. Blood was collected by tail snip (~10 μl) and transferred into blood collection tubes (Microvette CB 300 Z). Blood samples were stored on ice and then centrifuged at 5,000*g* for 5 min at 4 °C to collect serum. Tissue harvest was performed at the end of the infusion after euthanasia by cervical dislocation. Tissues were quickly dissected, rinsed in cold PBS, clamped with a pre-chilled Wollenberger clamp, and dropped in liquid nitrogen.

### Tail vein infusion

Mice were anaesthetized with 2% isoflurane inhalation and maintained under anaesthesia for the duration of the infusion. A 30G catheter was inserted into the mouse tail vein, and the stable isotopes were administered as described below. For glucose infusion, 0.2 M [U-^13^C] glucose was administered as a bolus of 1.6 μl g^−1^ min^−1^ for 2 min, followed by a constant infusion rate 0.1 μl g^−1^ min^−1^of for 30 min. For lactate infusion, 1.67% [U-^13^C] lactate was administered as a bolus of 0.96 μl g^−1^ min^−1^ for 2 min, followed by a constant infusion rate 0.3 μl g^−1^ min^−1^of for 30 min. Blood was collected retro-orbitally during the procedure, and tissues were harvested immediately at the end of the infusion as described for jugular vein catheter infusions.

### Tissue and serum metabolite extraction

Serum (4 μl) was diluted with 140 μl of ice-cold 80% methanol:20% water solution and vortexed. Seventy-two microlitres of chloroform was added, the solution vortexed again and centrifuged at 15,060*g* for 10 min at 4 °C to enforce phase separation. Aqueous supernatant was used for LC–MS analysis. For tissue extractions, 30-40 mg sections of snap frozen mouse tissue were transferred to pre-chilled Safe-Lock tubes (Eppendorf, 022363352) containing a cold 5/16-inch-diameter stainless steel ball (Grainger, 4RJL8). The tissue was disrupted by shaking at 25 Hz for 30 s under liquid nitrogen using the Retsch CryoMill (Retsch, 20.749.0001). Fifteen microlitres per milligram of tissue of −80 °C polar metabolite extraction solution containing 40:40:20 acetonitrile:methanol:water and 0.1% formic acid was added to homogenized tissue. Samples were briefly vortexed before neutralizing with 8 μl of 15% ammonium bicarbonate per 100 ml of extraction solvent. The solution was centrifuged at 16,000*g* for 10 min at 4 °C. The supernatant was collected and the pellet extracted a second time with 525 μl of chilled 40:40:20 methanol:acetonitrile:water. Both supernatants were combined and 525 μl of chloroform was added. The mix was vortexed and centrifuged at 16,000*g* for 10 min at 4 °C, and final aqueous layer transferred to LC–MS tubes for analysis.

### LC–MS analysis of polar metabolites

Extracted polar metabolite samples were analysed by LC–MS. Separation was achieved by hydrophilic interaction liquid chromatography (HILIC) using a Vanquish HPLC system (ThermoFisher Scientific). The column was an Xbridge BEH amide column (2.1 mm × 150 mm, 2.5 µm particle size, 130 Å pore size, Waters) run with a gradient of solvent A (20 mM ammonium hydroxide, 20 mM ammonium acetate in 95:5 acetonitrile:water, pH 9.5) and solvent B (100% acetonitrile) at a constant flow rate of 150 μl min^−1^. The gradient function was: 0 min, 90% B; 2 min, 90% B; 3 min, 75% B; 7 min, 75% B; 8 min, 70% B; 9 min, 70% B; 10 min, 50% B; 12 min, 50% B; 13 min, 25% B; 14 min, 25% B; 16 min, 0% B; 20.5 min, 0% B; 21 min; 90% B; 25 min, 90% B. Autosampler temperature was 4 °C, column temperature 30 °C and injection volume 3 µl. Samples were injected using electrospray ionization into a QExactive HF orbitrap mass spectrometer (ThermoFisher Scientific) operating in negative ion mode with a resolving power of 75,000 at *m*/*z* of 200 and a full scan range of 75–1,000. Data were collected using the Xcalibur software suite. Data were analysed using the EL-MAVEN software package and specific peaks assigned based on exact mass and comparison with known standards^56^. Extracted peak intensities were corrected for natural isotopic abundance using the R package AccuCor^57^. Untargeted LC–MS data were analysed using MetaboAnalyst.

### TCA cycle labelling contribution analysis

Total carbon atom fractional contributions were determined by dividing the natural isotope abundance corrected experimental labelling fractions by the number of carbons of each metabolite to determine a single carbon weighted labelling fraction for each metabolite. These were normalized to the serum tracer weighted labelling fraction (presented as 1 in all graphs)^38,58^.

### Langendorff ex vivo heart perfusion

Mice were administered heparin (100 IU, intraperitoneally) 20 min before anaesthesia with 3% isoflurane followed by cervical dislocation. Hearts were rapidly excised and cannulated on a Langendorff perfusion apparatus (flow rate: 2.6 ml min^−1^). The modified Krebs–Henseleit buffer contained 118 mM NaCl, 4.7 mM KCl, 2.5 mM CaCl_2_, 1.2 mM KH_2_PO_4_, 1.2 mM MgSO_4_, 24 mM NaHCO_3_, 0.5 mM EDTA, 0.7 mM oleic acid complexed to 2% albumin, 11 mM glucose, 50 μM L-carnitine, 1.5 mM lactate, 0.2 mM pyruvate, 0.8 nM insulin and 5 nM adrenaline to reproduce the non-fasted substrate and hormone conditions in mice as previously described^1^. The buffer was equilibrated with 95% O_2_/5% CO_2_ and maintained at 37 °C.

Hearts were retrograde perfused in non-recirculating mode for 30 min while beating spontaneously. For isotope tracing, unlabelled substrates were replaced with their labelled counterparts: [U-^13^C]glucose, [U-^13^C]lactate, or [2-^2^H]lactate. Electrocardiogram leads connected to an AD Instruments PowerLab 8/35 were positioned on the cannula (just above the aorta) and the apex of the heart to measure heart rate. Heart rate was averaged over a 30 s interval, 1 min before termination of perfusion. Hearts were clamped with a pre-chilled Wollenberger clamp and dropped in liquid nitrogen or mitochondria were isolated as described above.

### Adult cardiomyocyte isolation

Isolation of adult cardiomyocytes followed previously established protocols^16,38^. In brief, 12-week-old adult mice were anaesthetized with isoflurane before the heart was excised and attached to an aortic cannula. The heart was then perfused with oxygenated solutions maintained at 37 °C and pH 7.3. A 0 mM Ca^2+^ solution was perfused for 5 min, followed by a further 15 min of perfusion with the same solution containing 1 mg ml^−1^ collagenase and 0.1 mg ml^−1^ protease. This was succeeded by 1 min of perfusion with stopping solution (the same solution containing 20% serum and 0.2 mM CaCl_2_), all at a flow rate of 2 ml min^−1^. After removal of the atria, ventricles were teased apart with forceps, gently rocked for 10 min, and filtered through a nylon mesh. Following gravity sedimentation, the resulting cells were stored at 37 °C in normal HEPES buffered solution before plating as described below. The resulting isolated myocytes displayed a rod-shaped morphology with distinct striations and exhibited no spontaneous contractions.

### ACM culture and growth assays

ACMs were incubated with tracers in DMEM or human plasma-like medium (HPLM) as indicated. HPLM was made in-house as previously described^59^. Glucose or lactate was selectively excluded and replaced with heavy isotope tracers as required. Additionally, the plating media for both DMEM or HPLM consisted of 100 μg ml^−1^ Primocin, 100 U ml^−1^ penicillin-streptomycin, 10 mM HEPES, 5% dialysed fetal bovine serum, and 10 mM 2,3-butanedione monoxime. Primary adult cardiomyocytes were isolated (as described above) and enriched through gravity sedimentation in increasing concentrations of Ca^2+^. Following isolation, cells were plated on Laminin-coated Petri dishes or coverslips and allowed to adhere for at least 1 h in a 5% CO_2_ incubator. Subsequently, cells were transitioned to an HPLM or DMEM-based culture medium supplemented with Primocin, penicillin-streptomycin, HEPES, insulin-transferrin-selenium, 0.1 mg ml^−1^ BSA, and a physiological BSA-conjugated fatty acid mix, as previously described^60^.

### Permeabilized ACM assay and imaging

ACMs were isolated and cultured in HPLM without 2,3-butanedione monoxime and allowed to adhere for at least 1 h on a laminin-coated 24-well plate. Cells were washed with warm PBS followed by 5-min incubation with 0, 25 or 100 μg ml^−1^ saponin in mitochondrial assay buffer (MAB) for permeabilization. Cells were then washed 1× with MAB and incubated at 37 °C in MAB. MAB composition was as follows: 2 mM KH_2_PO4, 120 mM KCl, 3 mM HEPES, 1 mM EGTA, 3 g l^−1^ BSA (essentially fatty acid-free), 1 mM lactate, 50 μM pyruvate, and 1 mM glutamine. The pH of the buffer was adjusted to 7.2 using 5 N KOH. Incucyte Cytotox NIR dye (Sartorius 4846) was added at 0.6 μM during final MAB incubation and cells were imaged at 20× on Incucyte Live-Cell Analysis system using the near-infrared optical module (excitation 648–674 nm, emission 685–756 nm). After imaging, cells and supernatant (1:1) were collected in RIPA for downstream analysis of cytochrome *c* by immunoblotting. For confocal imaging, ACMs were plated on laminin-coated 35 mm Quad μ-Dish (ibidi 80416) and permeabilizaed as described above. After incubation in MAB, cells were incubated in 1 μg ml^−1^ DAPI (ThermoFisher D3571) for 5 min followed by incubation in 150 nM MitoTracker Red CMXRos (ThermoFisher M7512) and MitoTracker Green FM (ThermoFisher M7514) for 20 min. Cells were imaged at 40× magnification in Zeiss LSM 880 Airyscan confocal microscope. All cells were acquired at the same power and gain settings. Image analysis was performed using ImageJ.

For stable isotope tracing, ACMs were plated on laminin-coated 10 cm plate and permeated with 25 μg ml^−1^ saponin as described above. After 5 min, saponin was removed and new MAD containing [U-^13^C]lactate added. After 10 min with tracer, MAB was aspirated and metabolites were extracted with ice-cold 80:20 methanol:H_2_O as described above. For AZD (10 μM) treatment, inhibitor was added to the saponin incubation, PBS washes and maintained throughout tracing.

### Mitochondrial isolation

One whole mouse heart was homogenized in ice-cold CP-1 buffer (100 mM KCl, 50 mM Tris HCl, 2 mM EGTA; pH 7.4) using a Teflon pestle and glass tube (30–50 strokes, or until a homogenous mixture was obtained). The sample was transferred to a 1.5 ml Eppendorf tube and centrifuged at 700*g* for 10 min at 4 °C. The supernatant was then transferred to a new 1.5 ml Eppendorf tube and centrifuged at 10,500*g* at 4 °C. The supernatant was discarded and the pellet was washed with 1 ml fresh ice-cold CP-1 buffer, then centrifuged at 10,500*g*. Following 2 consecutive wash and spin steps, the final pellet was resuspended in 300 µl CP-1 buffer and protein concentration was determined using the Pierce Dilution-Free Rapid Gold BCA assay (ThermoFisher Scientific A55860). The same protocol was performed for isolation of mitochondria from ACMs (using ACMs from one mouse heart), frozen human heart samples, and HEK293T cells (40,000 cells, freshly pelleted and washed once with PBS). This preparation was used for metabolomics and respirometry experiments. For the immunoblotting, immunoprecipitation and proteomics experiments, these preparations were further purified by ultracentrifugation on a two-step (1.33 M, 1.55 M) sucrose gradient. Gradients were centrifuged at 4 °C for 1 h at 22,500 rpm. Mitochondria formed a compact, brown-coloured band at the interface. An 18-gauge needle was used to carefully recover the mitochondrial band for further analysis.

### Mitochondrial respiration measurements

Mitochondrial O_2_ utilization was measured using the Oroboros Oxygraph-2K system. Following BCA assay, freshly isolated mitochondria (25 µg) were added to the respirometry chambers containing 2 ml of assay buffer Z (pH 7.1, MES potassium salt 105 mM, KCl 30 mM, KH_2_PO_4_ 10 mM, MgCl_2_ 5 mM, BSA 1 mg ml^−1^). Respiration on lactate or pyruvate was measured with the following additional substrates: 0.5 mM pyruvate or 0.5 mM lactate with 1 mM ADP and 500 µM malate. Inhibitors of pyruvate and lactate metabolism were added to the buffer in the following concentrations: CPI-613 (PDH and αKGDH inhibitor) 240 μM, GSK 2837808 A (pan-LDH inhibitor) 8 μM, and 7ACC2 (MCT1 inhibitor) 10 μM or DMSO control.

### LDH activity assay

Mitochondria were freshly isolated as described above. For mitochondria pretreated with proteinase K, proteinase K was added to freshly isolated mitochondria in CP-1 buffer at a concentration of 5 µg proteinase K per µg isolated mitochondria. Samples were incubated on ice for 5 min, and proteinase K activity was quenched with PMSF (final concentration, 18 mM). Reactions were performed in black wall, black bottom 96-well plates. Freshly isolated mitochondria (15–30 µg) were added to 175 µl buffer D (105 mM MES-potassium, 30 mM KCl, 10 mM KH_2_PO_4_, 5 mM MgCl_2_, 1 mM EGTA, 2.5 g l^−1^ BSA, 5 µM rotenone). Buffer D was supplemented with alamethicin (final concentration 30 µg ml^−1^) and/or oxamate (final concentration 50 mM) where listed. Finally, lactate and NAD^+^ were added at final concentrations of 3 mM and 1 mM, respectively. NADH autofluorescence was measured at 20 s intervals for 20 min immediately following the addition of lactate and NAD^+^ at 360/460 nm (excitation/emission) using a Biotek Synergy Neo2 multimodal plate reader (Agilent Technologies).

### In vitro metabolite tracing

ACMs were plated as described in HPLM-FA medium. For tracing experiments, glucose- or lactate-free HPLM-FA medium was made in-house, and [U-^13^C]glucose or [U-^13^C]lactate was added to the medium. Cells were incubated for 4 h. Isolated mitochondria were treated identically as reactions were carried out at 37 °C in HPLM-FA medium with appropriate ^13^C-tracers and quenched with −80 °C cold methanol. Cell plates were washed with ice-cold PBS 2× and then 80:20 methanol:water was added at 60× the packed cell volume (MidSci, TP87005) count. The resulting mixture was incubated on dry ice, scraped, collected into a microfuge tube, vortexed, rested on dry ice for 5 min and centrifuged at 16,000*g* for 10 min. Supernatant was placed into a fresh tube which was then centrifuged again at 16,000*g* for 10 min. The supernatant was placed in an MS tube (Agilent 5188-2788) for downstream analysis.

### Rapid isolation of cardiomyocyte mitochondria from heart tissue

All procedures for mitochondria isolation were performed on ice. Heart tissue was rapidly excised and rinsed in ice-cold PBS prior to homogenization. Approximately 30 mg of heart tissue was homogenized in 2 ml of KPBS (135 mM KCl, 10 mM KH_2_PO_4_, pH 7.25, in LC–MS water) supplemented with 5 mM sodium oxamate using a Dounce homogenizer. The homogenate was centrifuged at 1,000*g* for 2 min at 4 °C. The resulting supernatant was subjected to immunoprecipitation with prewashed anti-HA beads for 3.5 min, followed by washing in KPBS 3 times. In the final wash, 10% of the bead suspension was reserved for protein extraction. The remaining beads were then extracted with a solution consisting of 80% (v/v) LC–MS grade methanol and 20% (v/v) LC–MS grade water, and the extracts were analysed with LC–MS.

### Immunoblotting

Samples were washed with PBS and lysed in RIPA buffer (50 mM Tris, 150 mM NaCl, 0.1% SDS, 0.5% sodium deoxycholate, 1% NP-40) containing protease and phosphatase inhibitors. Protein concentrations were determined using the Pierce BCA Protein Assay Kit. Samples were combined with 4× sample loading buffer and heated at 95 °C for 5 min. A total of 20 μg of protein lysate was separated on an SDS polyacrylamide gel following standard procedures at 20 mA per gel, then transferred to a 0.45 μm nitrocellulose membrane (GE Healthcare) using a Mini Trans-blot module (Bio-Rad) at a constant voltage of 100 V for 2 h. The membrane was blocked with 5% non-fat milk (Serva) in Tris-buffered saline with 0.05% Tween 20 (TBS-T) overnight at 4 °C and then incubated for 3 h to overnight in 5% non-fat milk or 5% bovine serum albumin (Sigma) in TBS-T with primary antibodies against MPC1 (Cell Signaling 14462, 1:1,000), MPC2 (Cell Signaling 46141, 1:1,000), VDAC (Cell Signaling 4866, 1:5000), MCT1 (Santa Cruz sc-365501, 1:500, Proteintech 20139-1-AP, 1:1,000), MCT2 (Invitrogen PA5-88516, 1:1,000), MCT4 (Proteintech 22787-1-AP, 1:1,000), LDHA (Proteintech 19987-1-AP, 1:1,000), LDHB (Proteintech 14824-1-AP, 1:1,000), COX IV (Cell Signaling 4850, 1:1,000), ATP5A (Cell Signaling 18023, 1:1,000), TOM20 (Cell Signaling 42406, 1:1,000), TOM70 (Santa Cruz sc-390545, 1:1,000), ACO2 (Cell Signaling 6922, 1:1,000), LC3B (Cell Signaling 2775, 1:1,000), OPA1 (Cell Signaling 80471, 1:1,000), Na, K-ATPase (Cell Signaling 3010, 1:1,000), calreticulin (Cell Signaling 2891, 1:1,000), TIM23 (Cell Signaling 34822, 1:1,000), AIF (Cell Signaling 4642, 1:1,000), citrate synthase (Cell Signaling 14309, 1:1,000), HA tag (Cell Signaling 3724, 1:1,000), vinculin (Cell Signaling 13901, 1:1,000) and α-tubulin (Cell Signaling 3873, 1:1,000, Proteintech 11224-1-AP, 1:1,000). The membrane was then washed with TBS-T and incubated with the appropriate fluorophore-conjugated secondary antibody (Rockland Immunochemical, 1:10,000) or HRP-conjugated secondary antibody in 1% non-fat milk/TBS-T for 30 min. After a final wash with TBS-T, fluorescence was detected using the Odyssey CLx imaging system (LI-COR Biosciences) or with incubation with SuperSignal West Pico PLUS Chemiluminescent Substrate (ThermoFisher) or Western Lightening Plus Chemiluminescent reagent (Revvity, NEL105001EA) and visualization using a Bio-Rad luminescent imaging system or exposure to X-ray film.

### MCT1 immunoprecipitation

For proteomic confirmation of mitochondrial MCT1 localization, mitochondria were purified from mouse hearts, subjected to anti-MCT1 immunoprecipitation, and analysed by LC–MS/MS. Mitochondrial preparations were washed twice with ice-cold PBS before being pelleted by centrifugation at 1200 rpm for 3 min. The pellets were lysed in 50 µl of RIPA lysis buffer on ice for 10–20 min. Lysates were then centrifuged at 16,100*g* for 10 min, and the supernatants were transferred to fresh tubes. For each condition, immunoprecipitations were performed as follows: Experimental immunoprecipitation: mouse anti-MCT1 was added to the supernatant at a ratio of 1.5–2 µl antibody per sample. Control immunoprecipitation: normal mouse IgG (Cell Signaling 61656) was diluted to match the concentration of the experimental antibody and added to a separate sample of lysate. Both immunoprecipitation reactions were incubated overnight at 4 °C with rotation. The next day, samples were washed three times with lysis buffer. For each immunoprecipitation, 25–35 µl of prewashed magnetic protein G beads were added to the samples, and incubated with rotation for 2 h at 4 °C. Beads were then collected by a magnet, and washed three times with lysis buffer. After washing, beads were eluted and immunoprecipitated proteins separated by SDS–PAGE and either immunoblotted for MCT1 or the MCT1-sized band was excised and sent directly for proteomic analysis. Gel bands were excised, and their protein content was digested in-gel with trypsin^61,62^. The extracted peptides were desalted via StageTip, dried using vacuum centrifugation, and reconstituted in 5% acetonitrile with 5% formic acid for LC–MS/MS processing^63^.

### Proteomics via tandem mass spectrometry

Mass spectrometric data were collected on Orbitrap Fusion Lumos instruments coupled to a Proxeon NanoLC-1200 UHPLC. The 100 µm capillary column was packed with 35 cm of Accucore 150 resin (2.6 μm, 150 Å; ThermoFisher Scientific) at a flow rate of 360 nl min^−1^. The scan sequence began with an MS1 spectrum (Orbitrap analysis, resolution 60,000, 350–1,350 Th, automatic gain control (AGC) target 100%, maximum injection time of 118 ms). Data were acquired for 60 min per sample. The hrMS2 stage consisted of fragmentation by higher energy collisional dissociation (HCD, normalized collision energy 36%) and analysis using the Orbitrap (AGC 2 × 10^5^, maximum injection time 60 ms, isolation window 1.2 Th, resolution 7.5 K). Data were acquired using the FAIMSpro interface the dispersion voltage set to 5,000 V, the compensation voltages were set at −40V, −60V, and −80V, and the TopSpeed parameter was set at 1 s per compensation voltage. Mass spectra were processed using a Comet-based in-house software pipeline. Mass spectra were converted to mzXML using a modified version of ReAdW.exe. Database searching included all entries from the human UniProt database (downloaded November 2021), which was concatenated with a reverse database composed of all protein sequences in reversed order. The digest was set to semi-tryptic. Searches were performed using a 50 ppm precursor ion tolerance and the product ion tolerance was set to 0.02 Th. Oxidation of methionine residues (+15.9949 Da) was set as a variable modification. PSM filtering was performed using a linear discriminant analysis, as described previously^64^, while considering the following parameters: XCorr (cross-correlation score), ΔCn (delta correction score), missed cleavages, peptide length, charge state, and precursor mass accuracy. Peptide-spectral matches were identified, quantified, and collapsed to a 1% false discovery rate and then further collapsed to a final protein-level false discovery rate of 1%.

### Proteinase K protection assay

Freshly isolated mitochondria (50–100 µg) were resuspended in 50–100 µl of the following assay buffers for a final concentration of 1 µg mitochondrial isolate per µl: isotonic (225 mM mannitol, 75 mM sucrose, 1 mM EGTA, 10 mM HEPES; pH 7.4), hypotonic (10 mM HEPES; pH 7.4), and detergent (isotonic buffer + 1% Triton X-100). For proteinase K-positive samples, buffers were prepared with Proteinase K at a final concentration of 5 µg µl^−1^. After 5 min (isotonic, hypotonic) or 10 min (detergent) of incubation on ice, PMSF was added to quench proteinase K activity (final concentration, 18 mM). Following gentle agitation and 5 additional minutes of incubation on ice, isotonic and hypotonic samples were centrifuged at 10,000*g* for 10 min at 4 °C. Supernatants were removed and pellets were resuspended in 50–100 µl of isotonic or hypotonic buffer containing 1 mM PMSF (for a final concentration of 1 µg mitochondrial isolate per µl, based on the initial protein concentration of mitochondrial isolate). 4× Laemelli buffer and 1 M DTT were added for a final concentration of 1× Laemelli buffer and 50 mM DTT. Samples were then boiled at 95 °C for 5 min. Finally, 15 µl of each sample was loaded onto SDS–PAGE gels for analysis.

### Quantitative PCR analysis

Total RNA from mouse hearts was isolated using RNeasy Mini Kits (Qiagen), according to the manufacturer’s instructions. Next, cDNA was synthesized using a cDNA Reverse Transcriptase Kit (New England Biolabs). TaqMan-based real-time quantitative PCR was then performed using a QuantStudio 7 Pro Real-Time PCR System (ThermoFisher). The housekeeping gene vinculin (*Vcl*) was used as an internal control for cDNA quantification and normalization of gene amplified products.

### In vitro hypoxia–reoxygenation injury

Imaging was performed as described previously^65^. In brief, isolated cardiomyocytes were suspended in HPLM-FA medium and placed in an airtight chamber at 37 °C. To stimulate ischemic conditions, the chamber was flushed with a gas mixture containing 95% N_2_ and 5% CO_2_ to deplete the oxygen in the chamber. The chamber was then sealed, and the cells left for 2 h at 37 °C. After 2 h, the cells were removed from the chamber and placed under normal cell culture conditions (37 °C, room air supplemented with 5% CO_2_) for an additional 2 h to reoxygenate and model reperfusion. Control cells were suspended in supplemented HPLM medium and kept at 37 °C, in room air with 5% CO_2_ for 4 h. For imaging, the cells were loaded with either 20 nM of TMRM (Thermo Fisher), 5 μM MitoSox (Thermo Fisher) or 5 μM X-Rhod1 (Thermo Fisher) to measure mitochondrial membrane potential, mitochondrial ROS levels and mitochondrial Ca^2+^ levels, respectively. The cells were then placed onto glass-bottomed dishes (MatTek) and imaged using a Leica SP8 confocal microscope. All three dyes were measured with an excitation and emission at 548 nm and 574 nm. All cells were imaged at the same power and gain settings and images were collected within 30 min after removal from incubator following simulated reperfusion. Image analysis was performed using ImageJ^66^.

### Blue Native PAGE and immunoblotting

Crude mitochondrial fractions were normalized to total protein content and separated by 3–12% BN-PAGE (Invitrogen, BN2011BX10). In brief, 100 µg of mitochondria from *Mct1*^*fl/fl*^ or *Mct1*^*iCKO*^ mice hearts were lysed on ice in 100 µl of 1× pink NativePAGE sample buffer (Invitrogen, BN2008) containing 1% digitonin for 15 min and spun down at 4 °C for 20 min at 20,000*g*. Cleared mitochondrial lysate was transferred to a new 1.5 ml Eppendorf tube and 5 µl of NativePAGE 5% G-250 sample additive (Invitrogen, BN2008) was added. 10 µg of mitochondrial protein extracts were separated by 3–12% BN-PAGE using the Invitrogen NativePAGE system (Invitrogen, BN2007). Mitochondrial proteins were transferred to a polyvinylidene difluoride membrane (Millipore Sigma, IPVH00010) and blocked with 5% non-fat dry milk. Immunoblotting was performed using an OXPHOS antibody cocktail as primary antibody at 1:1,000 dilution (Abcam, ab110413). The membrane was then incubated with the Immun-Star Goat Anti-Mouse (Bio-Rad Laboratories, 1705047) horseradish peroxidase-conjugated secondary antibody at 1:10,000 dilution and developed with Western Lightning Plus chemiluminescent detection solutions (NEL105001EA; Revvity) and exposed to X-ray film.

### Seahorse assay

The Seahorse Mitochondrial Stress Test was performed following the manufacturer’s instructions on a Seahorse XF Pro Analyzer. Adult cardiomyocytes were plated in a 96-well seahorse plate in HPLM medium without BSA-conjugated fatty acids. The Seahorse assay was performed using 3 μM oligomycin, 1 μM FCCP and 1 μM rotenone/1 μM antimycin A, with a standard protocol of three measurement cycles for each phase (2 min mixing, 3 min waiting and 3 min measuring). After the assay, data were analysed in the Seahorse WAVE software through the XF Mito Stress Test Report.

### Transmission electron microscopy

After euthanasia, the mouse coronary artery was retrogradely perfused through the aorta with 20 ml of ice-cold fixative (formaldehyde/glutaraldehyde 2.5% in 0.1 M sodium cacodylate buffer). All fixatives and chemicals used for transmission electron microscopy are from Electron Microscopy Sciences. After perfusion, left ventricles were sliced into 1 mm^3^ tissue sections, stored at 4 °C in fixative, and sent to the Electron Microscopy Laboratory at University of Utah for further processing. After post-fixation with 2% osmium tetroxide and prestaining with uranyl acetate, the ventricular tissue slices were dehydrated in graded ethanol and pure acetone, imbedded with epoxy resin, and sectioned at 70 nm using an ultramicrotome (Leica). Sections were poststained with acetate and lead citrate before imaging with JEM-1400Plus or JEM1200-EX (JEOL) transmission electron microscope with a CCD Gatan camera.

### Mitochondrial morphology and cristae content analysis

Mitochondrial morphology and cristae content were analysed from transmission electron microscopy images using ImageJ software. The outer boundary of each mitochondrion, along with the contours of individual cristae within each mitochondrion, were manually traced using the freehand selection tool. Morphological parameters including mitochondrial area, circularity, roundness and the ratio of height/width were quantified to characterize mitochondrial shape. The surface area of each crista was measured, and the total cristae surface area per mitochondrion was calculated as the sum of the individual cristae areas. Cristae volume density was estimated by dividing the total cristae surface area by the corresponding mitochondrial area. Matrix volume density was subsequently derived by subtracting the cristae volume density from 100%.

### Statistical analysis

We used GraphPad Prism software (v10.3.1) for all statistical analysis. To determine if our data were normally distributed, we used the Shapiro–Wilk and D’Agostino–Pearson omnibus tests. For in vivo experiments, we utilized Grubb’s method (extreme Studentized deviate (ESD) to detect one outlier and robust outlier detection (ROUT) to detect multiple outliers) to expose any significant outliers (*P* < 0.05) in our data. If outliers were detected, they were removed subsequent statistical analysis. For all our data we present it as the mean ± s.e.m. and applied two-tailed tests for all comparisons. When analysing two discrete groups (such as *Mct1*^*fl/fl*^ versus *Mct1*^*iCKO*^), we used an unpaired Student’s *t*-test for normally distributed data, if the data were non-parametric, we used the Mann–Whitney *U* test. Multiple comparisons correction for unpaired *t*-tests was performed using the Holm–Sídák method for adjusted *P* values. For datasets with more than two variables one and two-way ANOVA was used with Tukey’s multiple comparison test. For evaluating serial echocardiographic data and multiple group comparisons, we used a repeated measures ANOVA (mixed-effects model) with a Geisser–Greenhouse correction, followed by a Tukey’s multiple comparison test for individual comparisons. We set an *α* level of *P* < 0.05 a priori and any value below this setpoint was considered statistically significant.

### Ethics statement

Animal experiments were conducted in accordance with the institutional guidelines for the care and use of laboratory animals. Our protocols were reviewed and approved by the Institutional Animal Care and Use Committee (IACUC) are the University of Utah (21-02003). For experiments involving the use of human myocardial tissue, our ethical approval was obtained from the Institutional Review Board (IRB) at the University of Utah (00030622) and all patients provided written informed consent prior to their inclusion in the study.

### Reporting summary

Further information on research design is available in the Nature Portfolio Reporting Summary linked to this article.

## Online content

Any methods, additional references, Nature Portfolio reporting summaries, source data, extended data, supplementary information, acknowledgements, peer review information; details of author contributions and competing interests; and statements of data and code availability are available at https://doi.org/10.1038/s41586-026-11182-3.

## Supplementary information

Supplementary Data Unprocessed western blots for Figs. 1-4 and Extended Data Figs. 2, 4, 6, 7, 10 and 12

Reporting Summary

Supplementary Table 1 Angiotensin phenylephrine echocardiograhy *P* values

Supplementary Table 2 Trans-aortic constriction echocardiograhy *P* values

Supplementary Table 3 IP–MS proteomics

Supplementary Table 4 ATII/PE metabolomics

Peer Review File

## Source data

Source Data Figs. 1–4 and Source Data Extended Data Figs. 1–5 and 7–12

## Data Availability

All analysed data including ^13^C mass spectrometry labelling data and western blots used in this study are included in the source data. Immunoprecipitation MS/MS protein ID data are in Supplementary Table 3. Metabolomics data from ATII/PE-treated *Mct1*^*iCKO*^ hearts are in Supplementary Table 2. Data from single-nuclei RNA sequencing from human heart were previously published and are available from Gene Expression Omnibus (GEO) under accession GSE226314. Source data are provided with this paper.
