# Supplementary material for: MCT1-dependent mitochondrial lactate import sustains cardiac metabolism
Source: Nature. 2026 Oct 28;659(8140):523–31. doi: 10.1038/s41586-026-11182-3 (PMC13754610; doi:10.1038/s41586-026-11182-3)

nature portfolio

https://doi.org/10.1038/s41586-026-11182-3

Supplementary information

MCT1-dependent mitochondrial lactate import sustains cardiac metabolism

In the format provided by the
authors and unedited

Unprocessed immunoblots related to Figure 1J

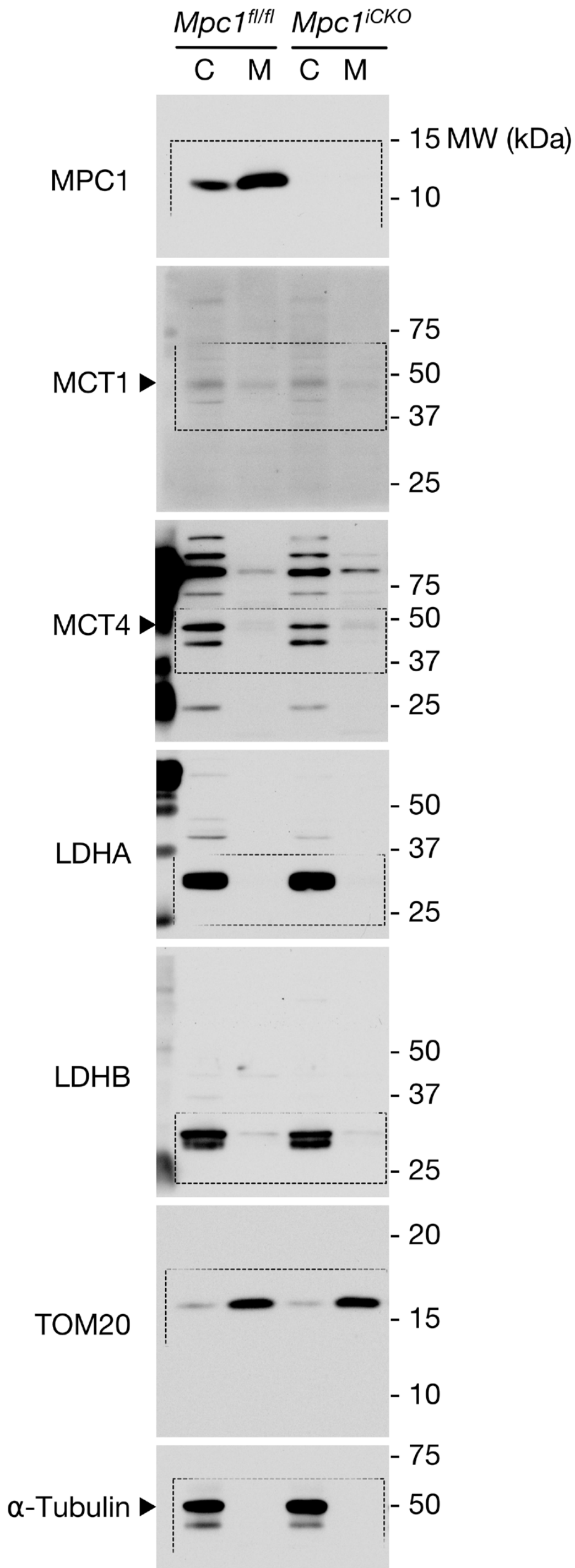

Unprocessed immunoblots
related to Figure 2C

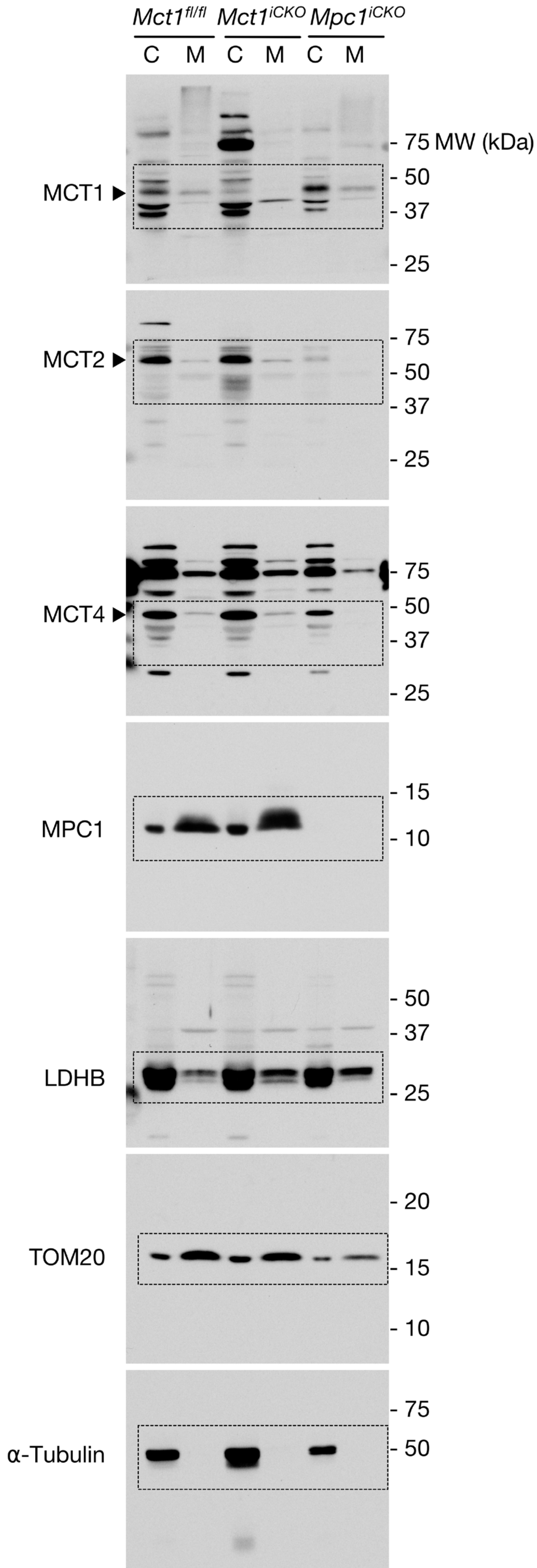

Unprocessed immunoblots
related to fig 3a

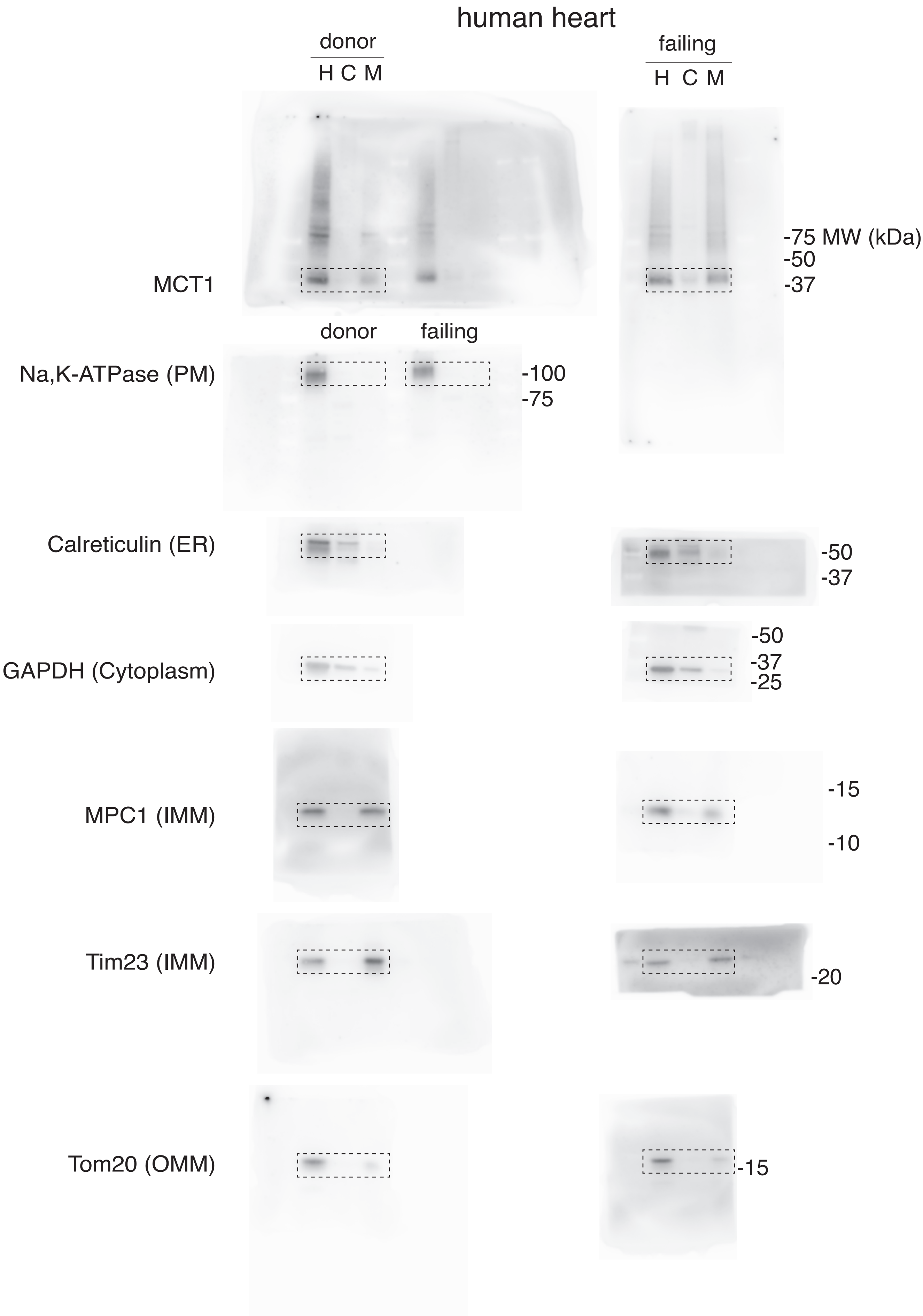

Unprocessed immunoblots
related to fig 3b

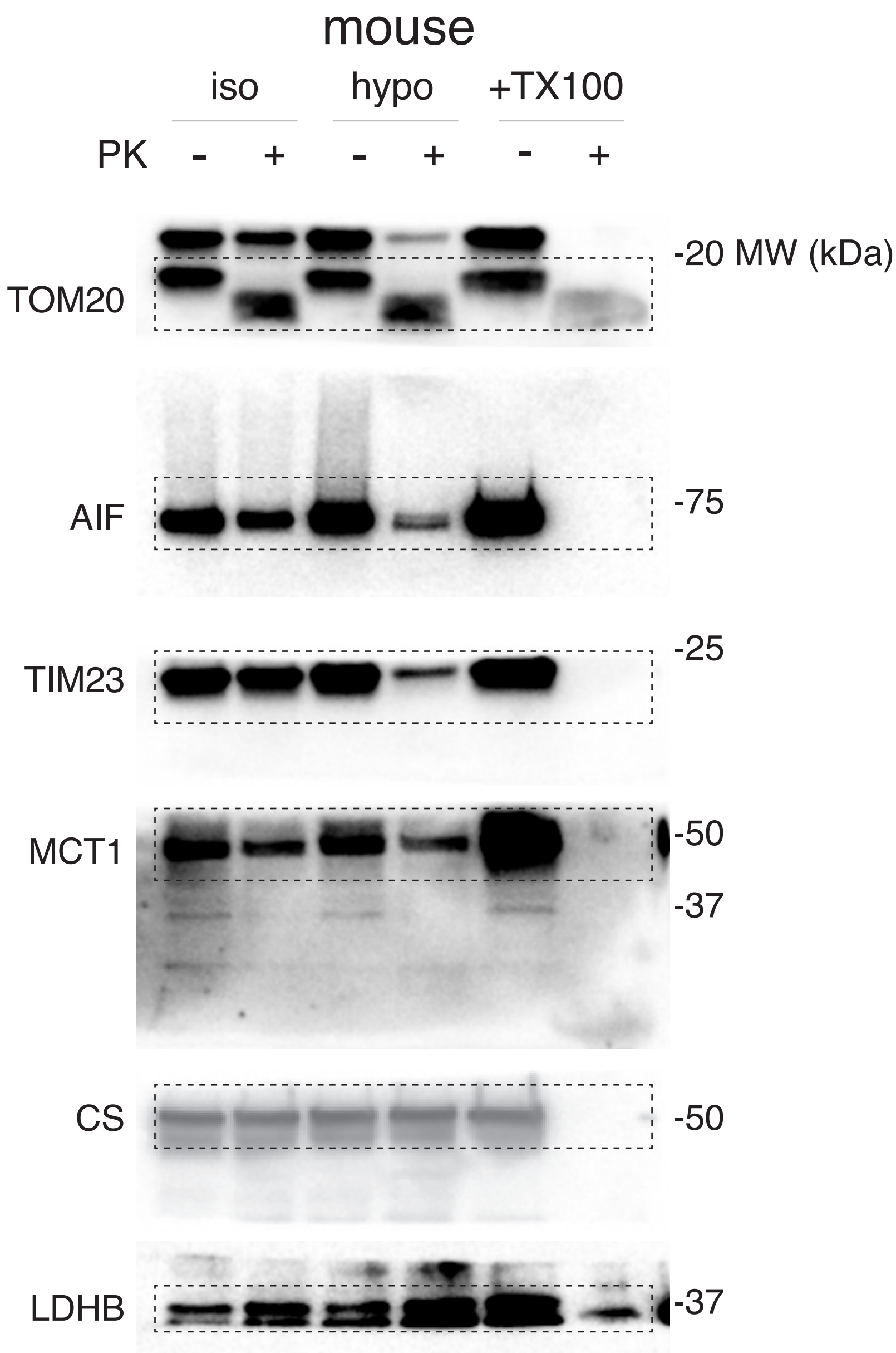

Unprocessed immunoblots related to fig 3c

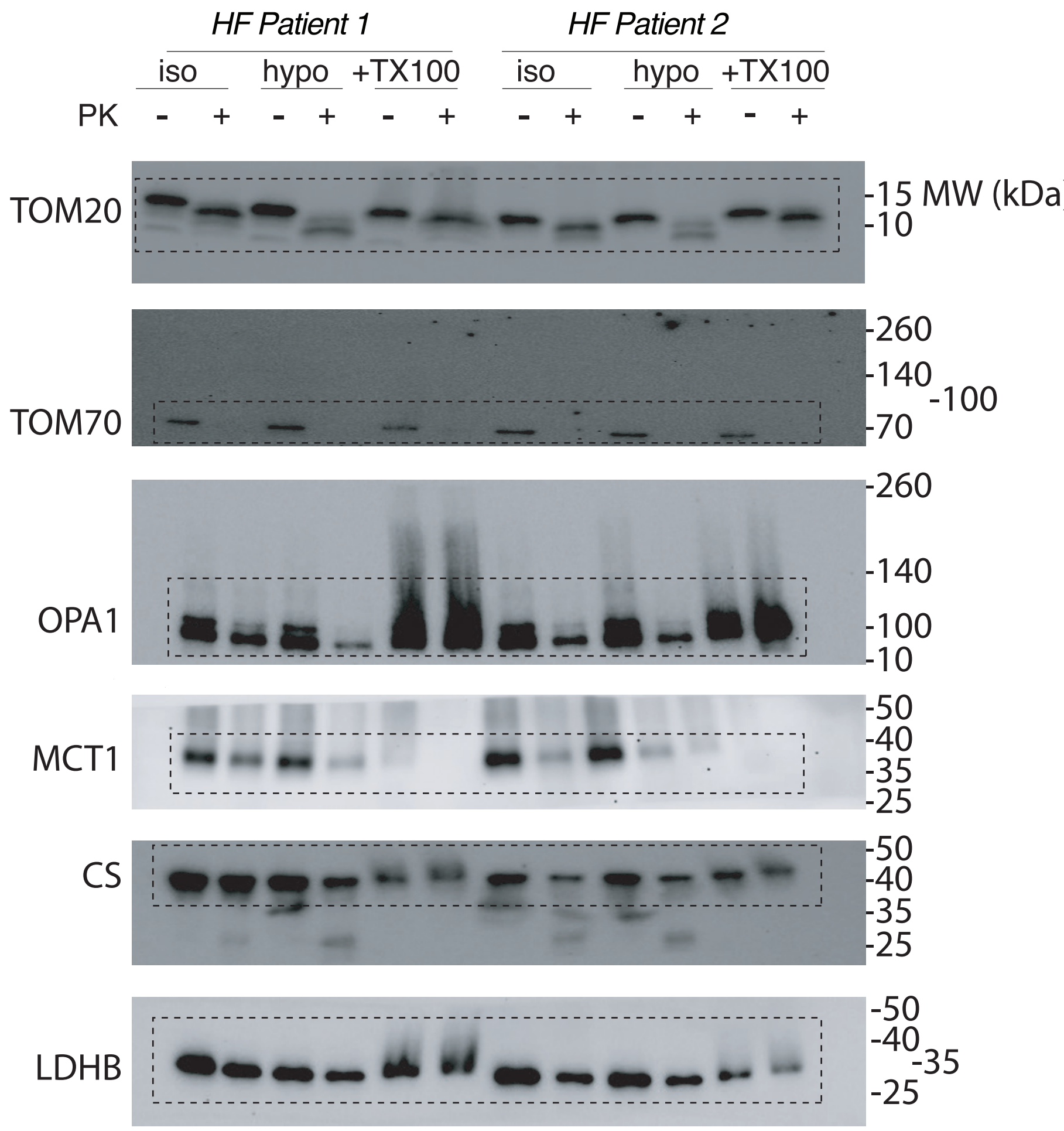

Unprocessed immunoblots related to fig 3e

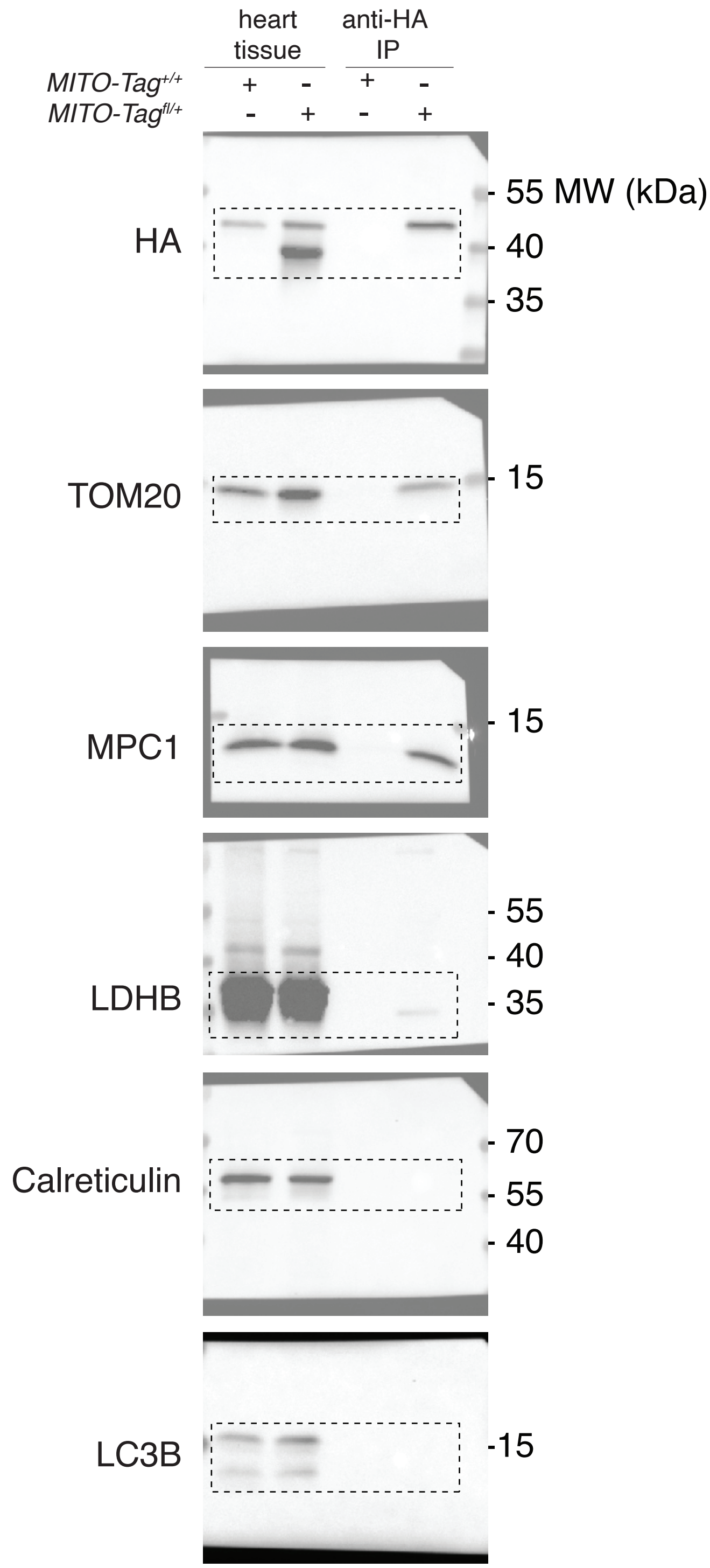

Unprocessed immunoblots
related to Figure 4G

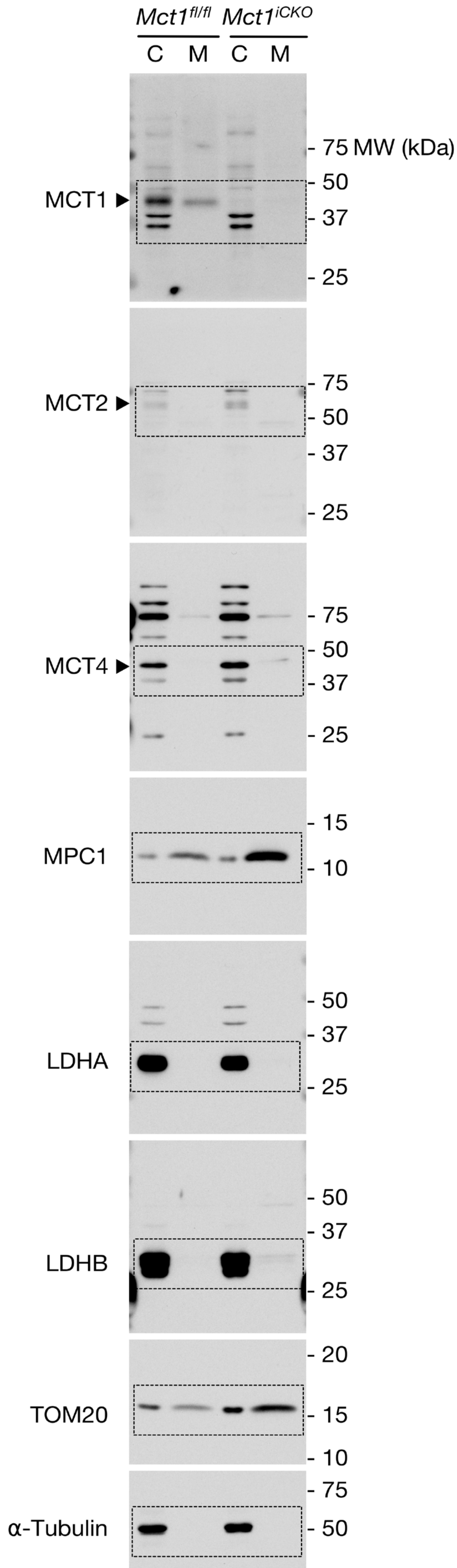

Unprocessed immunoblots
related to fig 4P

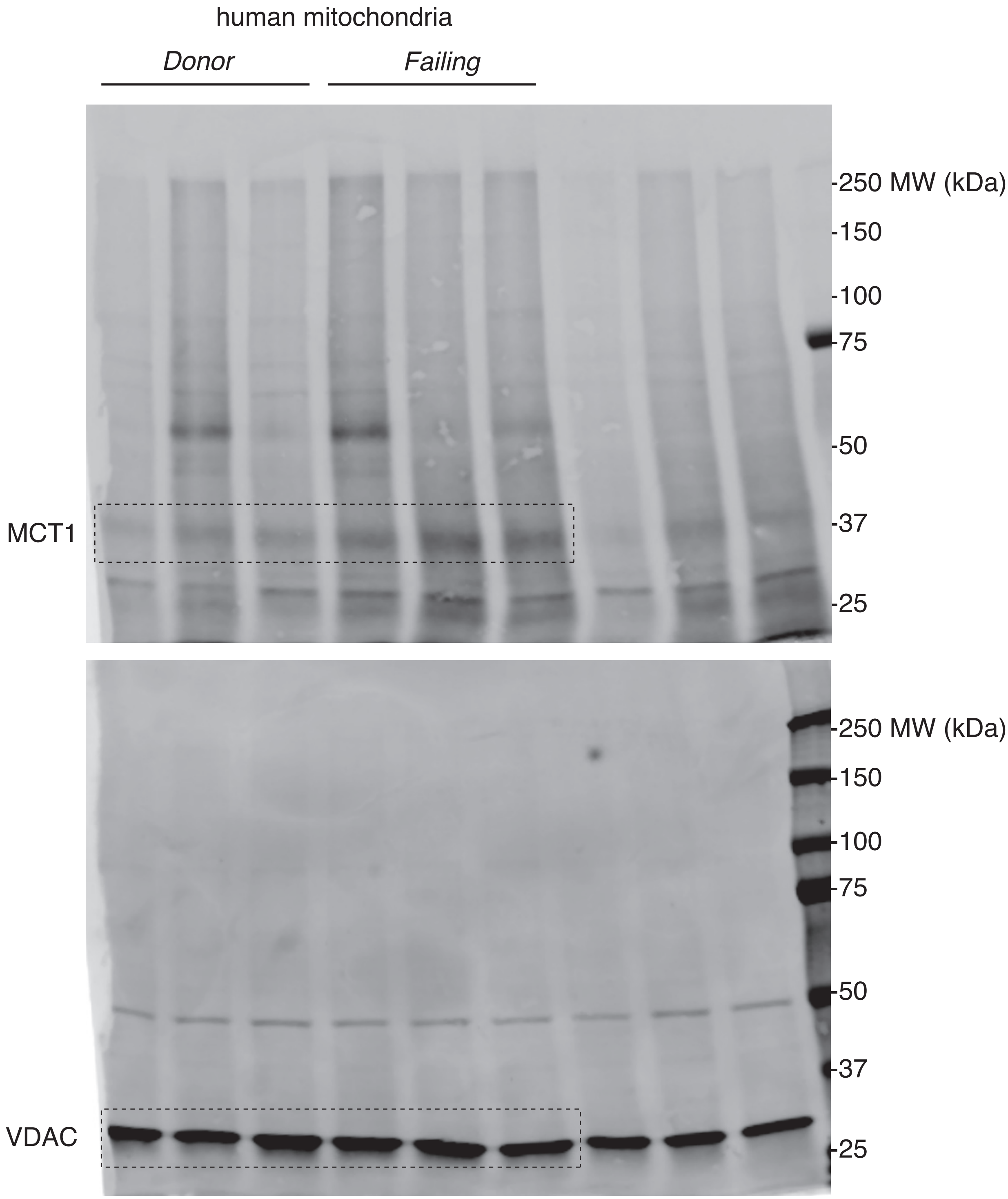

Unprocessed immunoblots
related to ext fig 2h

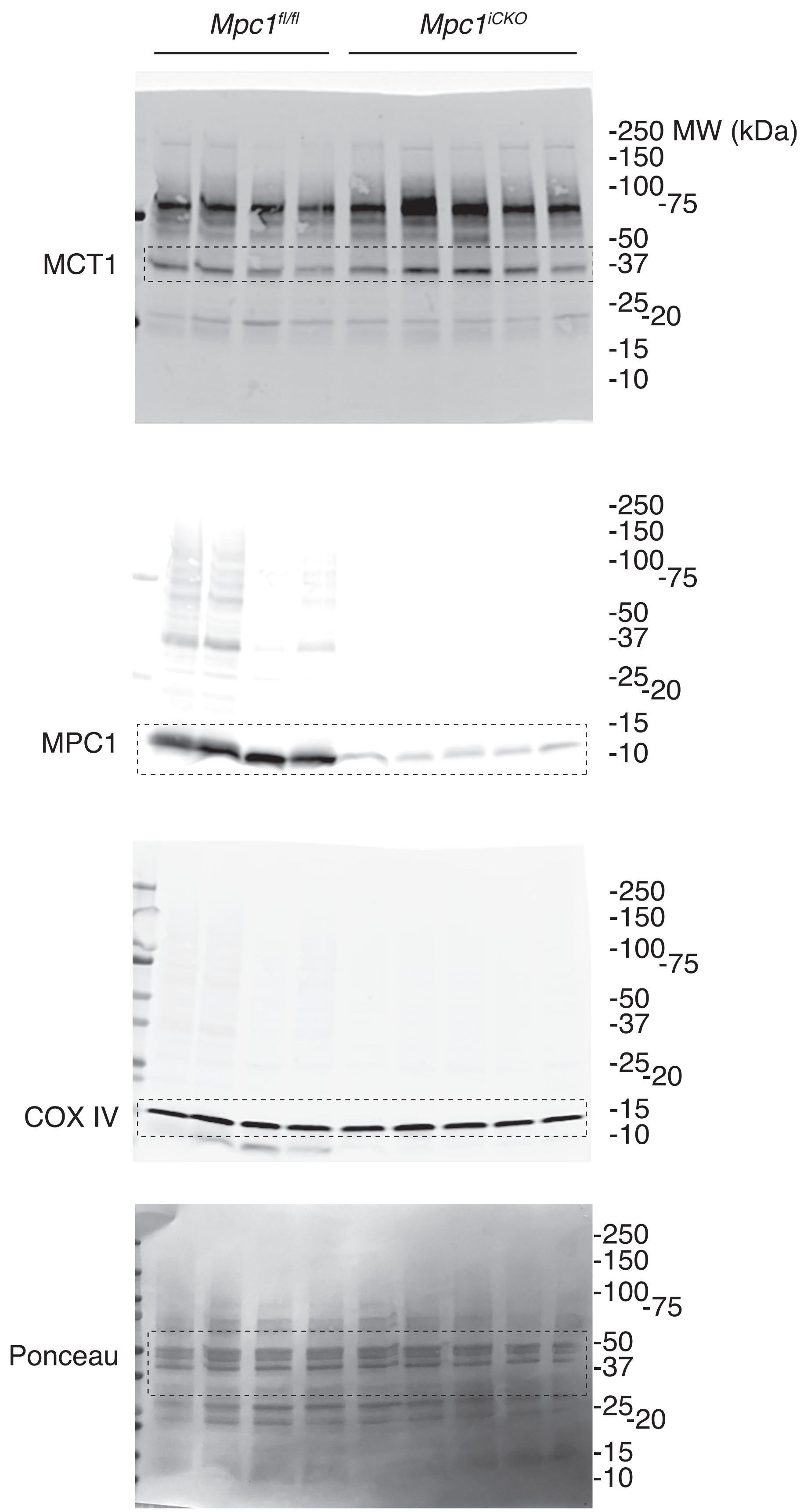

Unprocessed immunoblots
related to ext fig 4g

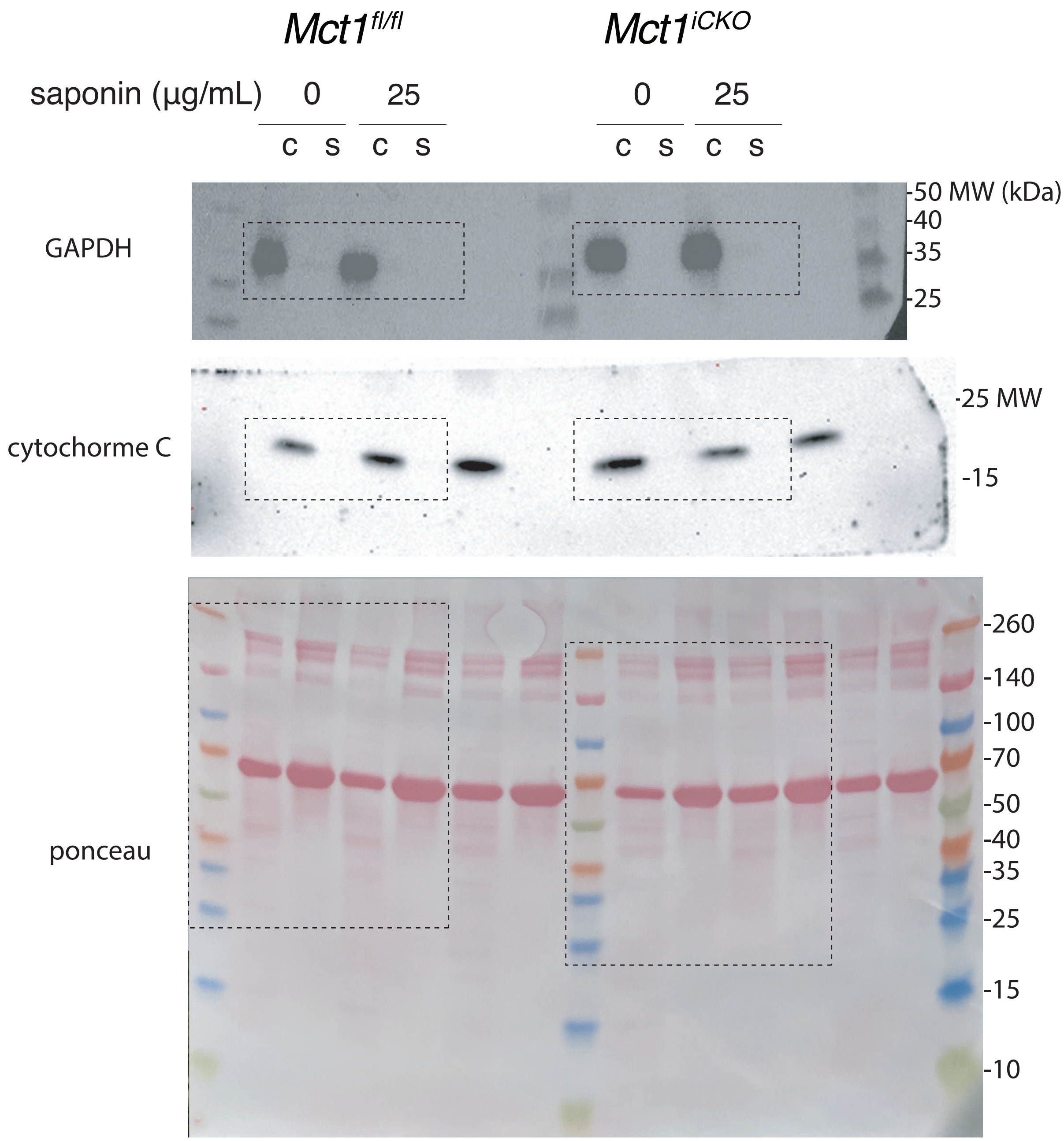

Unprocessed immunoblots
related to ext fig 6b

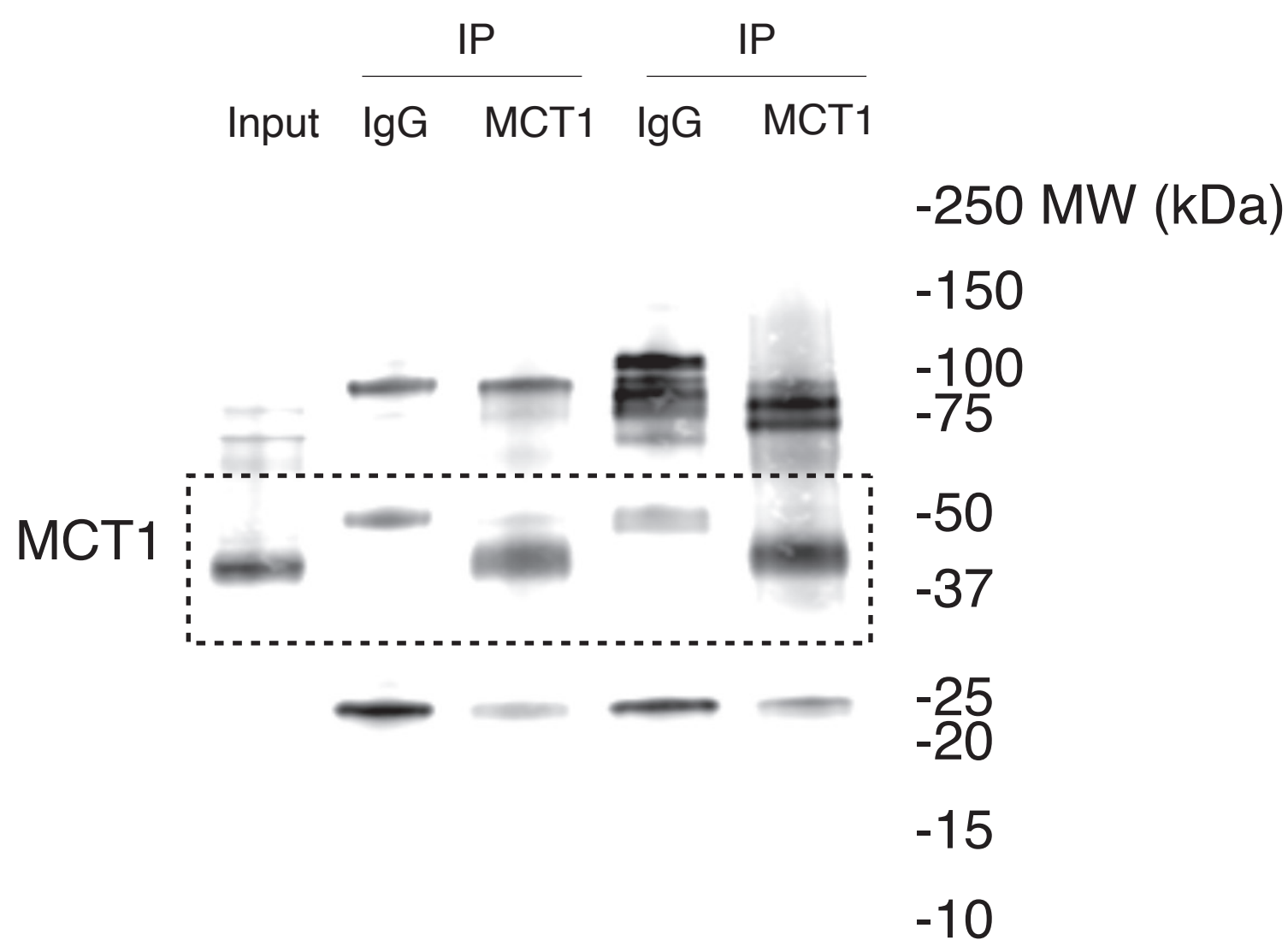

Unprocessed immunoblots related to ext fig 6e

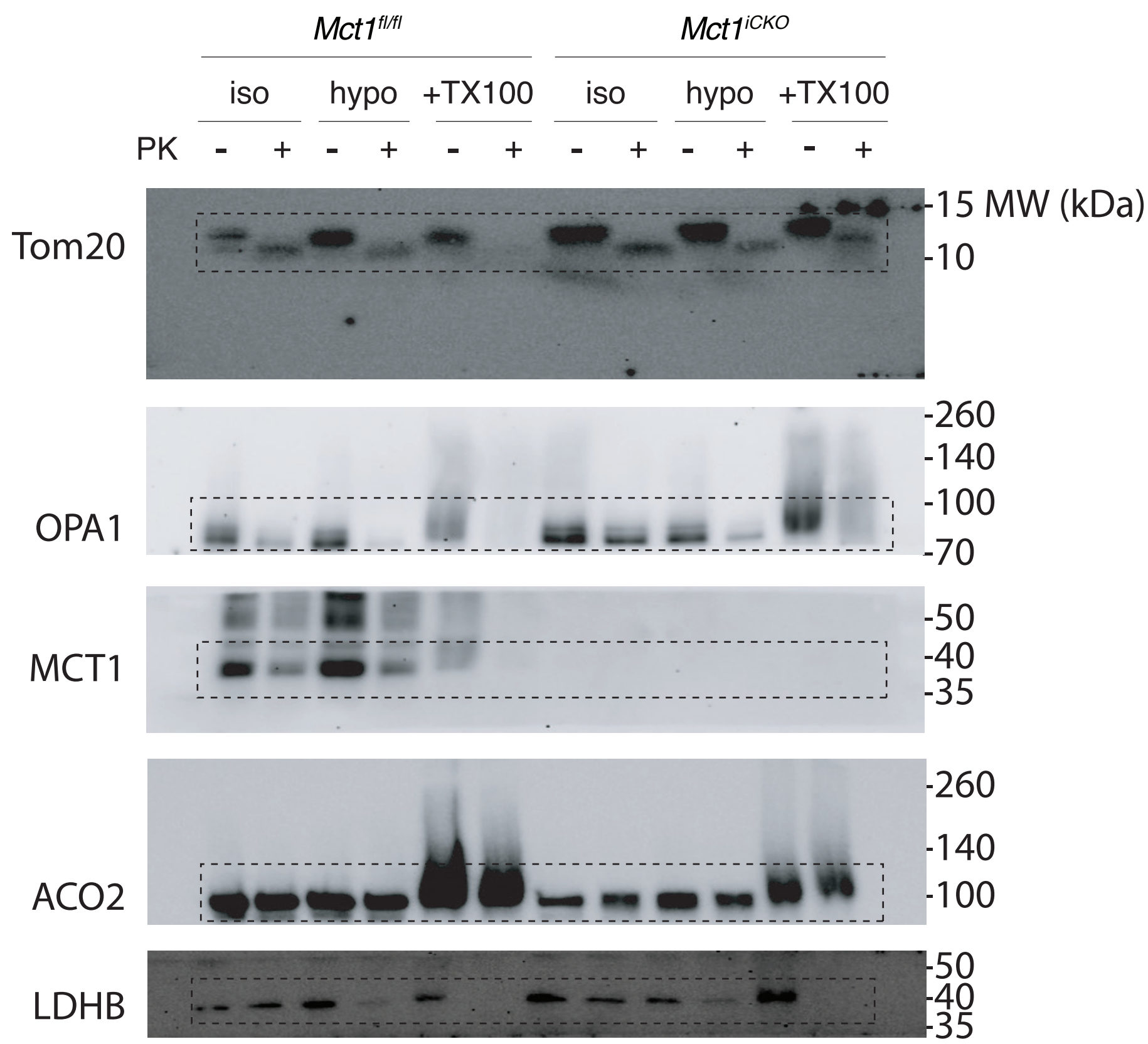

Unprocessed immunoblots related to ext fig 6f

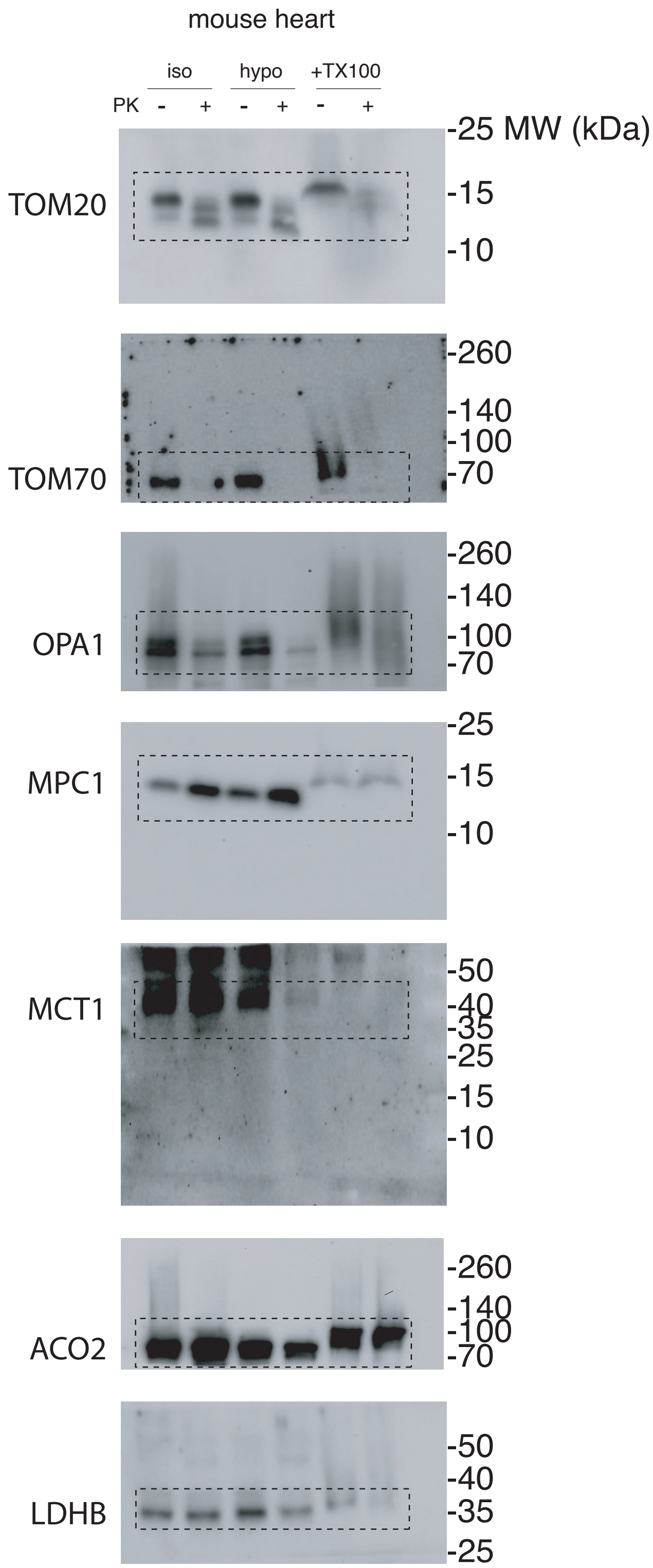

Unprocessed immunoblots
related to ext fig 6g

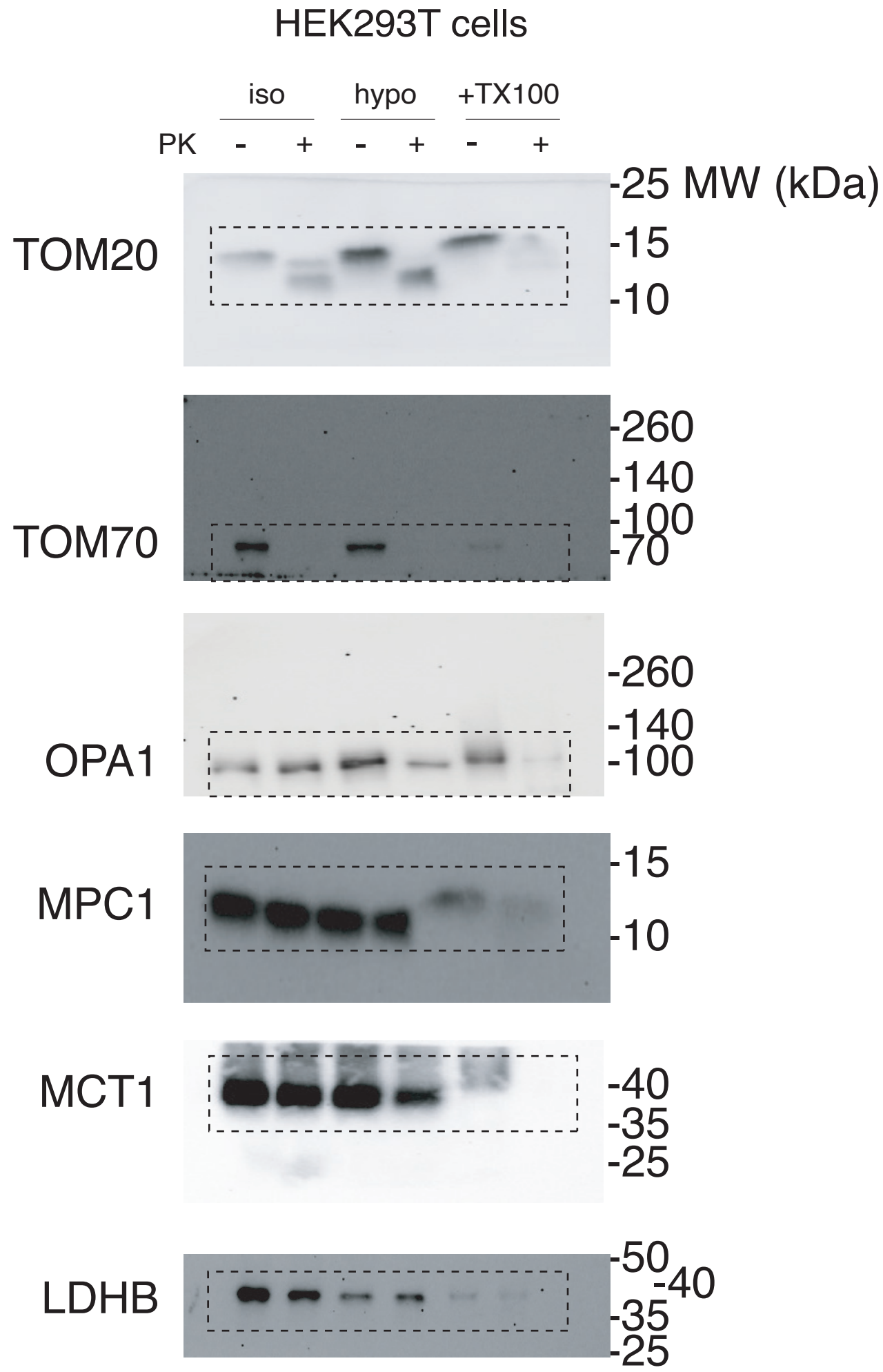

Unprocessed immunoblots related to ext fig 6h

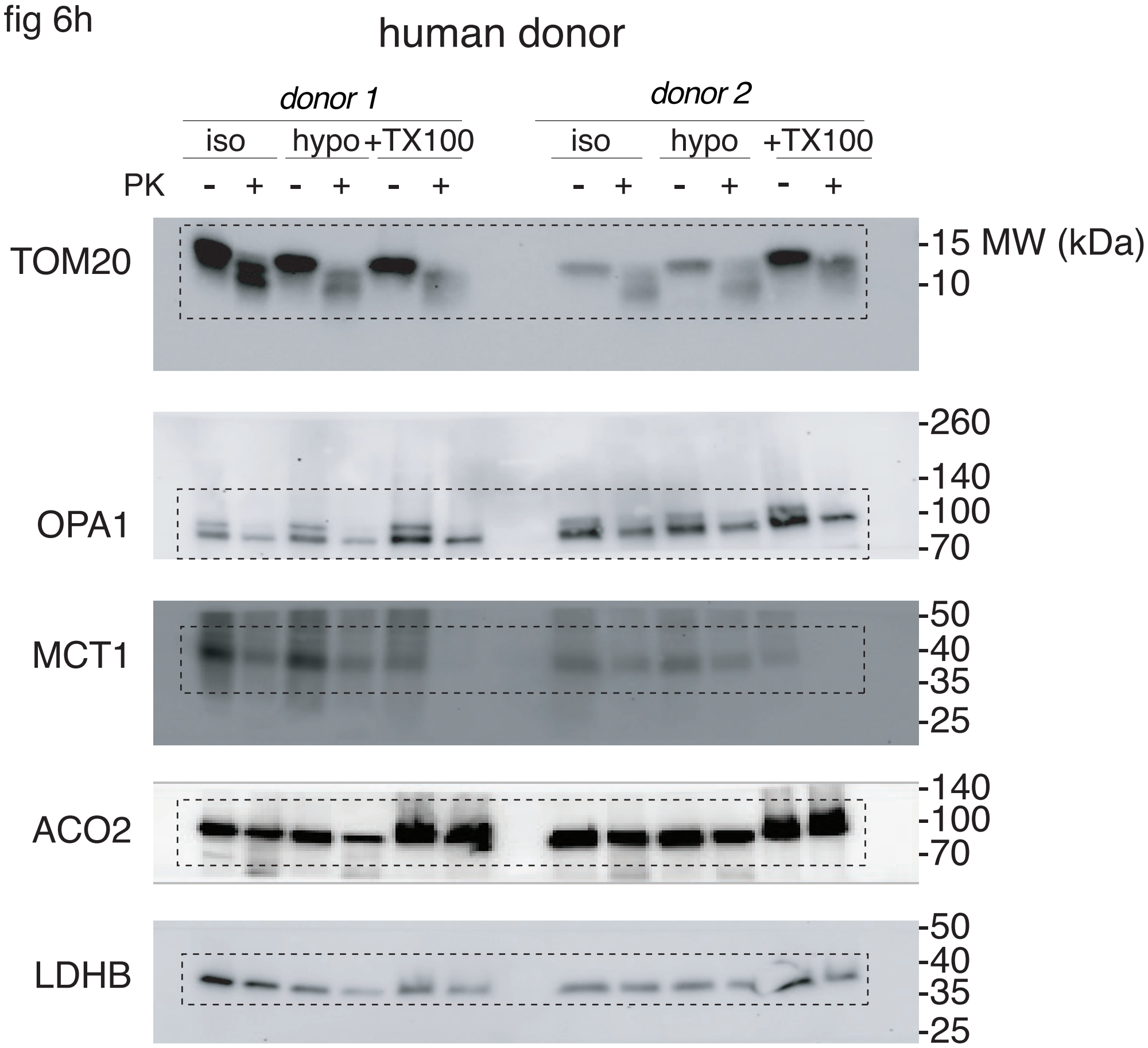

Unprocessed immunoblots related to ext fig 7d

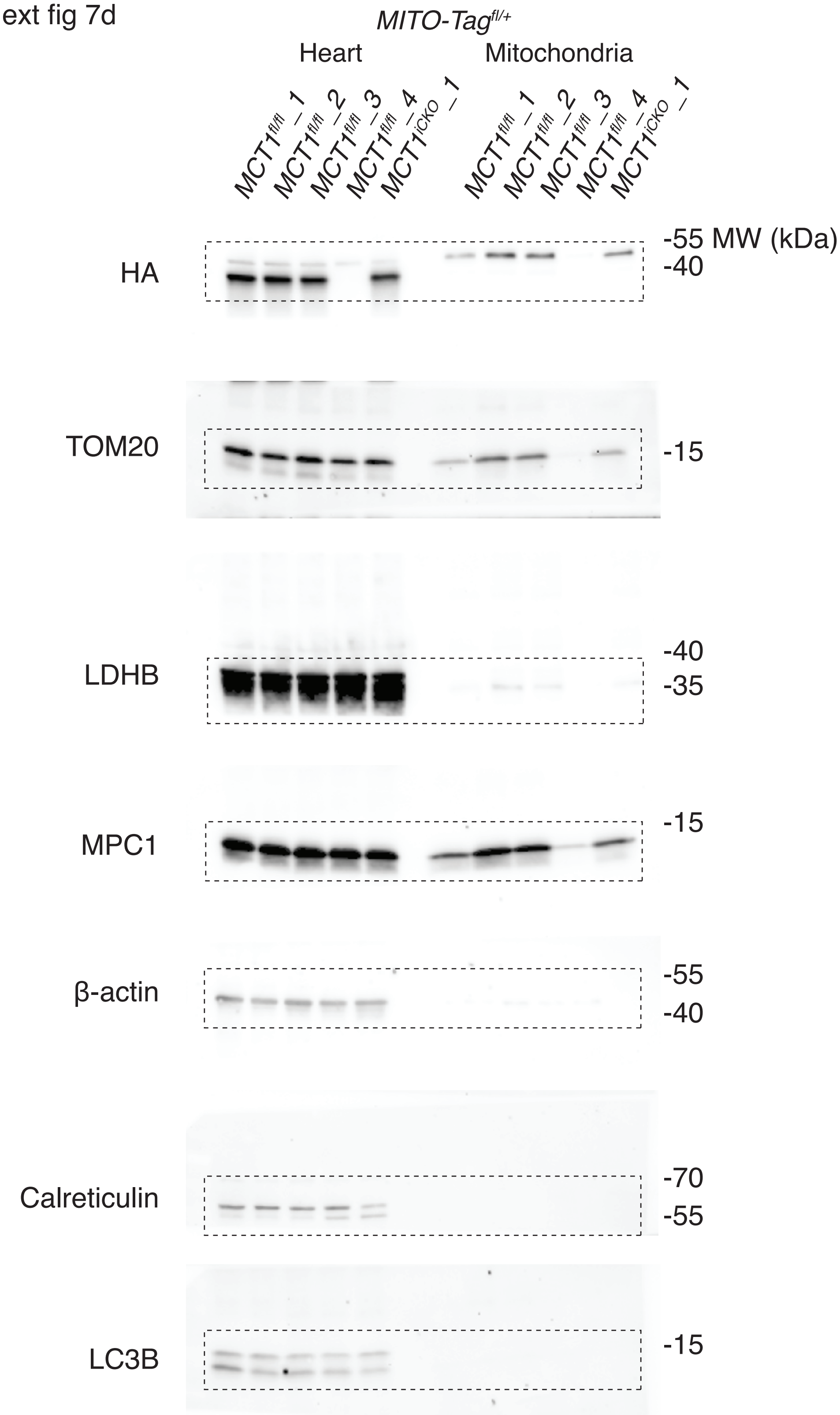

Unprocessed immunoblots related to ext fig 7e

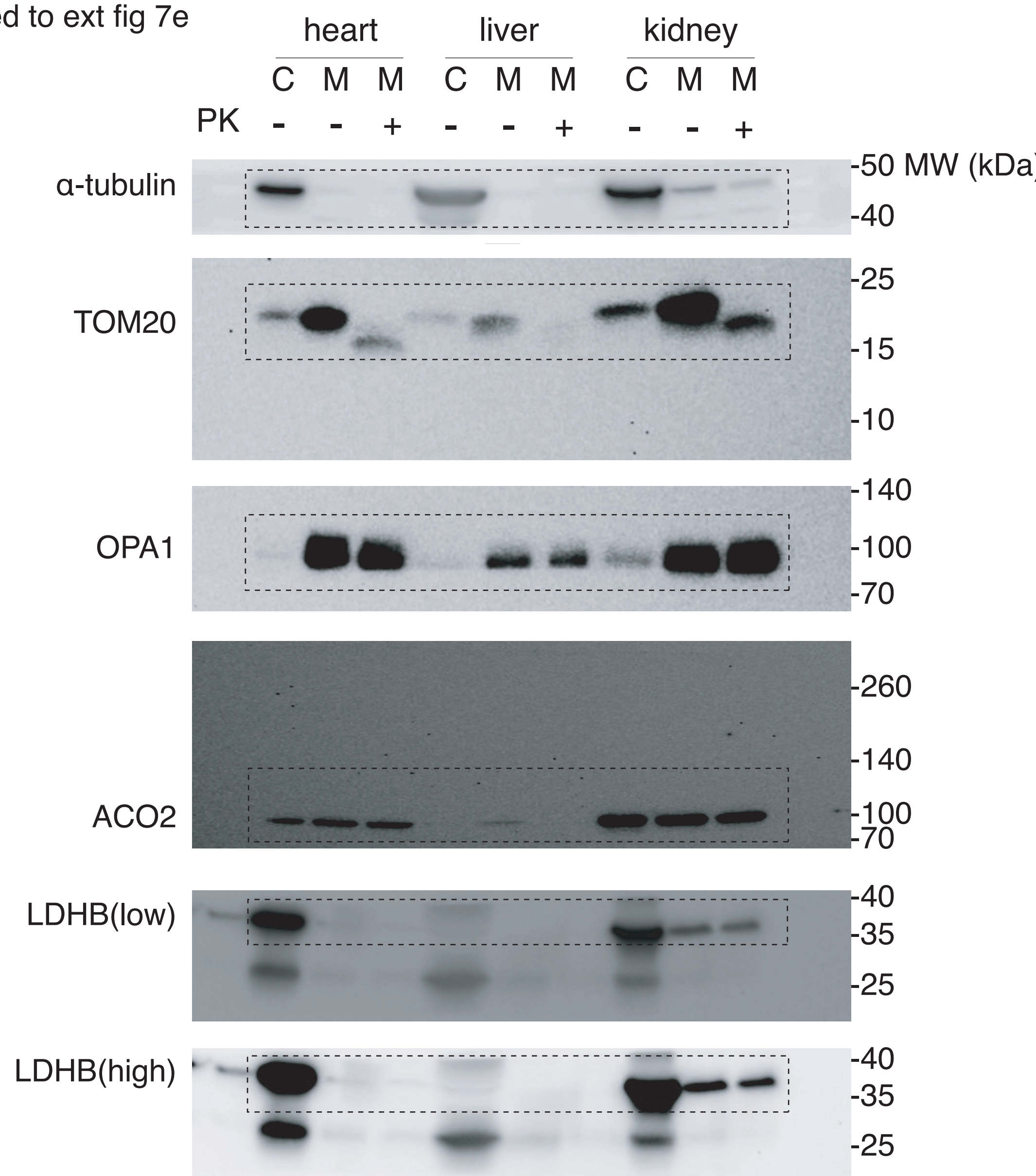

Unprocessed immunoblots
related to ext fig 10o

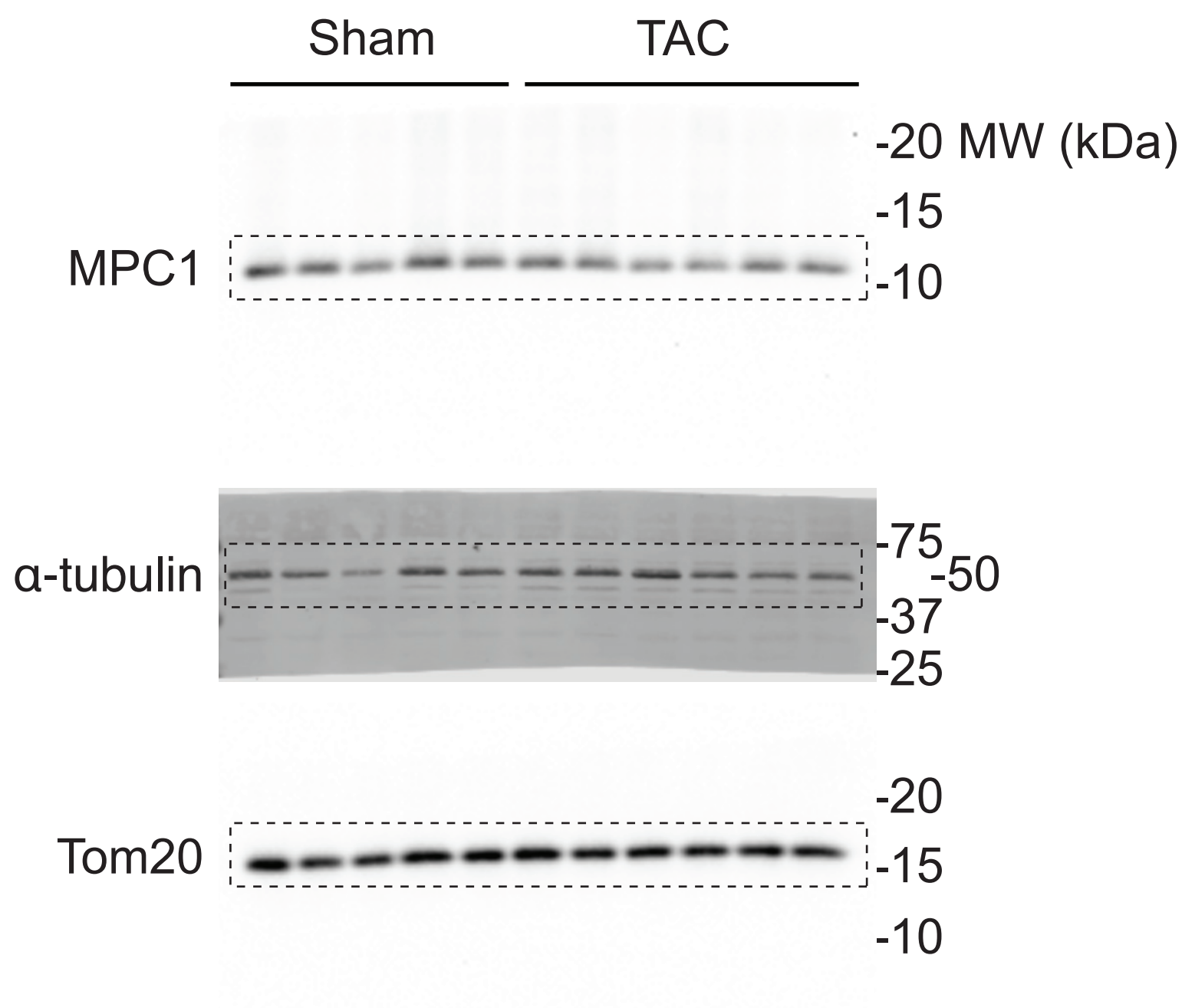

Unprocessed immunoblots
related to ext fig 12o

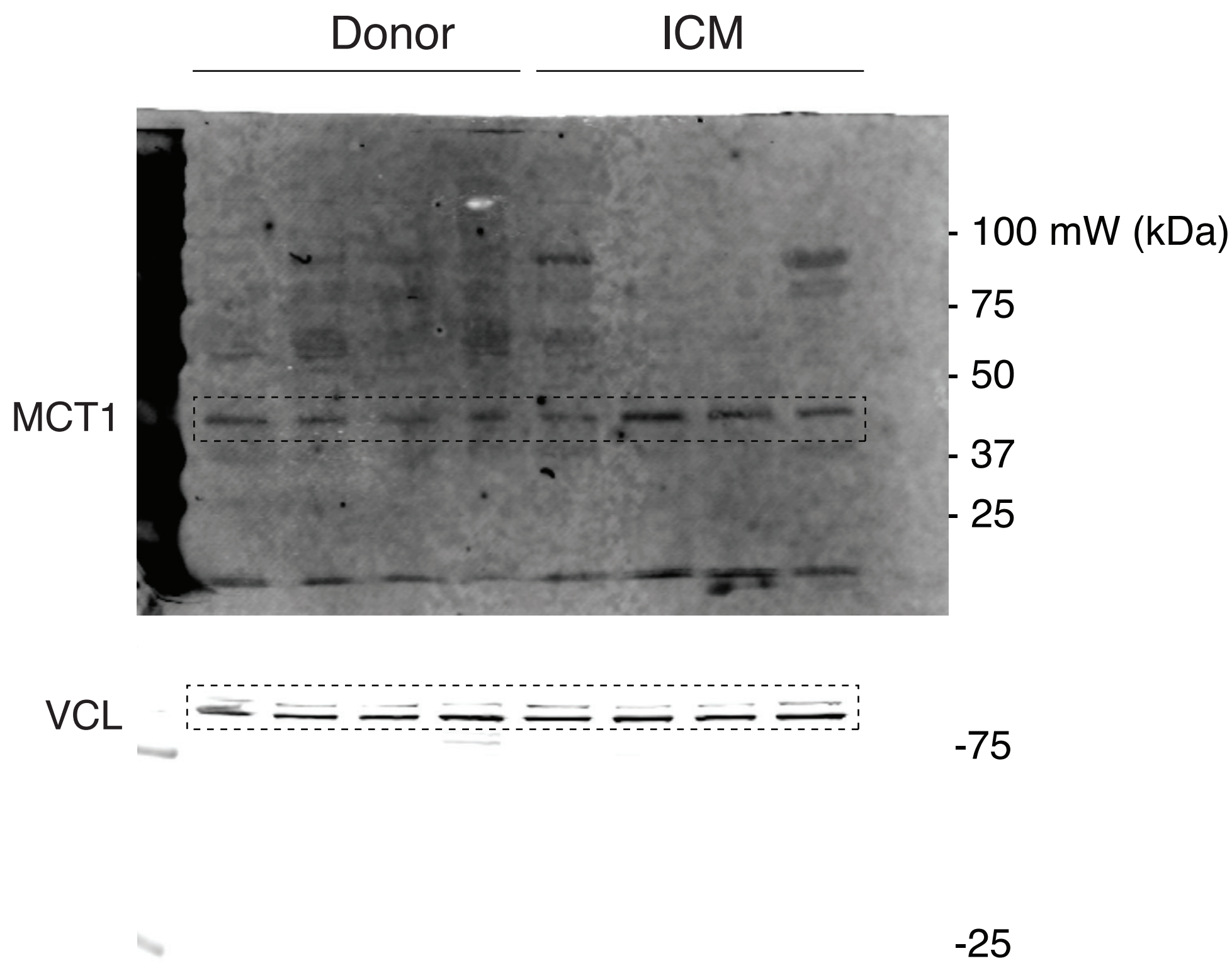

Unprocessed immunoblots
related to ext fig 12s

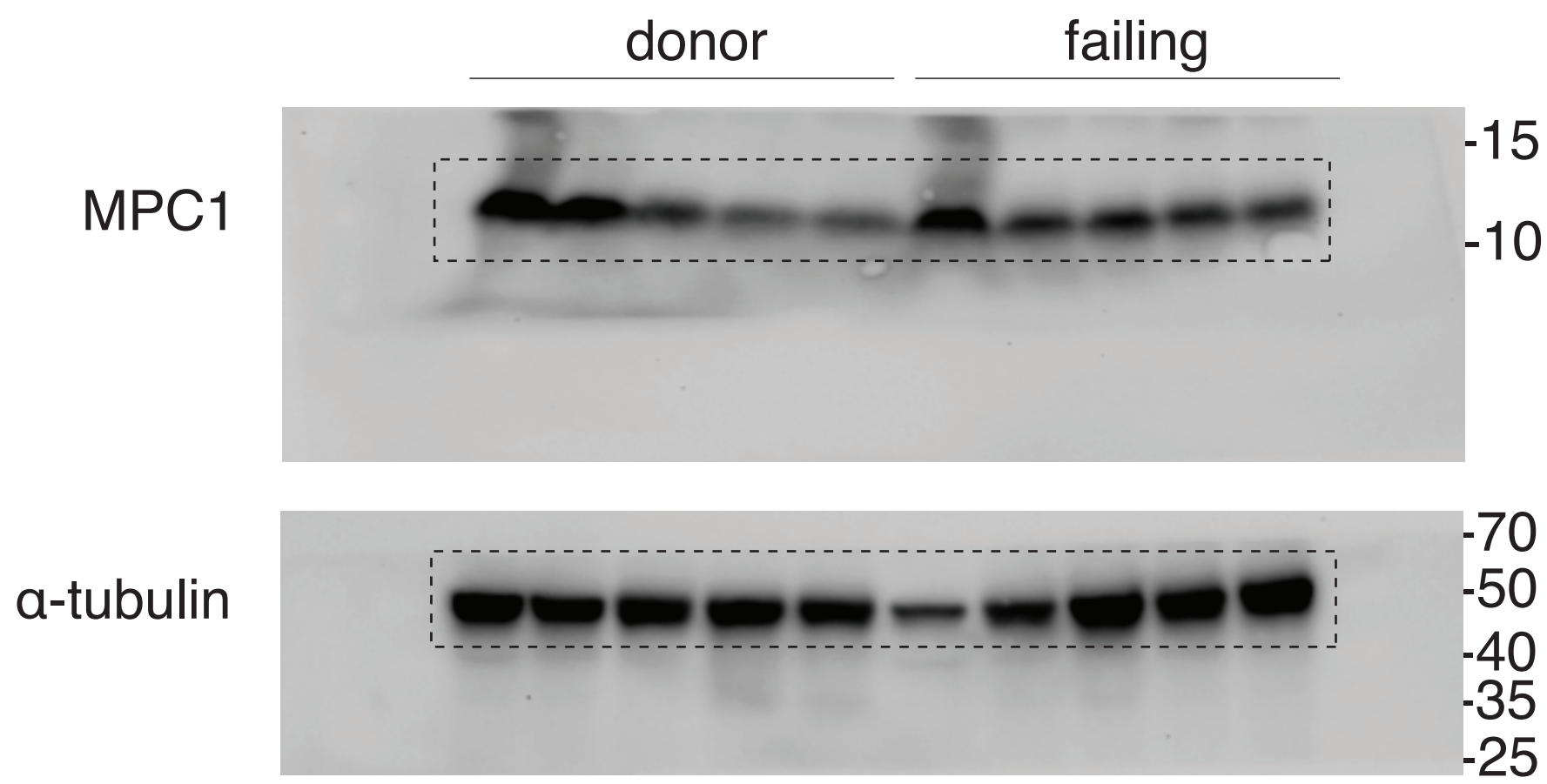

Supplement: Supplementary file 1 [file 41586_2026_11182_MOESM1_ESM.pdf]
