# Supplementary material for: MCT1-dependent mitochondrial lactate import sustains cardiac metabolism
Source: Nature. 2026 Oct 28;659(8140):523–31. doi: 10.1038/s41586-026-11182-3 (PMC13754610; doi:10.1038/s41586-026-11182-3)
Supplement: Supplementary file 2 [file 41586_2026_11182_MOESM2_ESM.pdf]

nature portfolio

Corresponding author(s): Drakos SG
Rutter J
Ducker GS

Last updated by author(s): Aug 7, 2026

Reporting Summary

Nature Portfolio wishes to improve the reproducibility of the work that we publish. This form provides structure for consistency and transparency in reporting. For further information on Nature Portfolio policies, see our Editorial Policies and the Editorial Policy Checklist.

Statistics

For all statistical analyses, confirm that the following items are present in the figure legend, table legend, main text, or Methods section.

n/a	Confirmed	
☐	☒	The exact sample size (*n*) for each experimental group/condition, given as a discrete number and unit of measurement
☐	☒	A statement on whether measurements were taken from distinct samples or whether the same sample was measured repeatedly
☐	☒	The statistical test(s) used AND whether they are one- or two-sided<br>*Only common tests should be described solely by name; describe more complex techniques in the Methods section.*
☒	☐	A description of all covariates tested
☐	☒	A description of any assumptions or corrections, such as tests of normality and adjustment for multiple comparisons
☐	☒	A full description of the statistical parameters including central tendency (e.g. means) or other basic estimates (e.g. regression coefficient) AND variation (e.g. standard deviation) or associated estimates of uncertainty (e.g. confidence intervals)
☐	☒	For null hypothesis testing, the test statistic (e.g. *F*, *t*, *r*) with confidence intervals, effect sizes, degrees of freedom and *P* value noted<br>*Give P values as exact values whenever suitable.*
☒	☐	For Bayesian analysis, information on the choice of priors and Markov chain Monte Carlo settings
☒	☐	For hierarchical and complex designs, identification of the appropriate level for tests and full reporting of outcomes
☒	☐	Estimates of effect sizes (e.g. Cohen's *d*, Pearson's *r*), indicating how they were calculated

Our web collection on statistics for biologists contains articles on many of the points above.

Software and code

Policy information about availability of computer code

Data collection	Mass spectrometry data collected using Thermo Scientific Xcalibur (version 4.2.47). Echocardiography data collected using Vevo Strain software (version 3.1.1).
Data analysis	Statistical analysis performed using GraphPad Prism 10 for macOS (version 10.6.1). Mass spectrometry data extraction and analysis conducted with El-MAVEN (version 0.12.0). Natural isotope abundance correction performed using R package 'Accucor' (version 0.3.1.9000). Untargeted mass spectrometry comparisons performed by MetaboAnalyst (version 6.0). Image quantification conducted with ImageJ (version 2.16.0/1.54p).

For manuscripts utilizing custom algorithms or software that are central to the research but not yet described in published literature, software must be made available to editors and reviewers. We strongly encourage code deposition in a community repository (e.g. GitHub). See the Nature Portfolio guidelines for submitting code & software for further information.

Data

Policy information about availability of data

All manuscripts must include a data availability statement. This statement should provide the following information, where applicable:

- Accession codes, unique identifiers, or web links for publicly available datasets
- A description of any restrictions on data availability
- For clinical datasets or third party data, please ensure that the statement adheres to our policy

All analyzed data including 13C MS labeling data and western blots used in this study are included in the source data. Immunoprecipitation MS/MS protein ID data are in Supplemental Data File A. Metabolomics data from Ang/PE treated Mct1iCKO hearts are in Supplemental Data File B. Data from single nuclei RNAseq from human heart was previously published and is available on the Gene ExpressionOmnibus (GSE226314). Complete source data are provided with this paper.

Research involving human participants, their data, or biological material

Policy information about studies with human participants or human data. See also policy information about sex, gender (identity/presentation), and sexual orientation and race, ethnicity and racism.

Reporting on sex and gender	No patient data was reported by sex/ gender. All patient data used in this study was de-identified.
Reporting on race, ethnicity, or other socially relevant groupings	No patient data was reported by race, ethnicity or other social grouping.
Population characteristics	For human data presented in this research, we did not include any population characteristics
Recruitment	Human myocardial tissue samples were obtained through the Utah Cardiac Recovery Program at the University of Utah under an approved IRB protocol (IRB-00030622). Participants were not recruited through public advertisement or volunteer enrollment. Rather, tissue was collected from patients with advanced heart failure undergoing clinically indicated heart transplantation who had provided informed consent for research use of explanted tissue. Control samples were obtained from non-failing donor hearts that were deemed unsuitable for transplantation for non-cardiac reasons (e.g., donor-recipient size mismatch or infection) and were available through established organ procurement processes with appropriate consent. This study has potential sources of selection bias. First, the HF cohort represents individuals with advanced heart failure who progressed to heart transplantation and may not represent well patients with earlier stages of HF. Second, the non-failing donor cohort may differ from the general healthy population because donor hearts were selected based on organ availability. Third, participation was limited to individuals who provided consent for tissue donation and research use, introducing the possibility of self-selection bias. However, because enrollment was based on tissue availability rather than study-specific recruitment, we believe any self-selection bias is unlikely to systematically affect the molecular and metabolic comparisons that constitute the primary outcomes of this study. Nevertheless, these factors may limit the generalizability of our findings beyond the patient populations studied.
Ethics oversight	University of Utah IRB Protocol #00030622

Note that full information on the approval of the study protocol must also be provided in the manuscript.

Field-specific reporting

Please select the one below that is the best fit for your research. If you are not sure, read the appropriate sections before making your selection.

☒ Life sciences ☐ Behavioural & social sciences ☐ Ecological, evolutionary & environmental sciences

For a reference copy of the document with all sections, see nature.com/documents/nr-reporting-summary-flat.pdf

Life sciences study design

All studies must disclose on these points even when the disclosure is negative.

Sample size	Sample size selection for in vivo mouse studies was based on previously published literature in the field directly applicable to our studies (PMID: 33333007, PMID: 32791100, PMID: 36725930). In vitro biochemical study sample size was set as a minimum of n=3. No specific calculations were performed for in vitro studies. For studies of human tissue, sample size was based on tissue available at the time of experimentation.
Data exclusions	Data exclusions for in vivo echocardiography data were performed using Grubbs' test and ROUT testing to identify and exclude outliers from the analysis. The exclusion criteria, determined by these outlier tests, were pre-established to ensure consistency and objectivity in the analysis. Grubbs' test was used to identify single outliers in normally distributed data, while ROUT testing was employed to detect multiple outliers using a Q coefficient to balance sensitivity and specificity. Outliers identified by these methods ($p<0.05$) were excluded to improve the accuracy and reliability of the findings. A small number of mice were excluded from isotope tracing studies due to technical failure of the infusion process (infused isotope enrichment <25% of the mean for all animals in the experiment).

Replication	Replication was obtained by performing experiments in multiple independent cohorts of animals from different litters analyzed independently. Final data presented from animal isotope tracing studies, isolated ACM studies and echocardiography studies was obtained from multiple cohorts of mice and presented together. PK studies, mitochondrial fractionation studies, and tracing studies in purified adult cardiomyocytes were carried out by independent researchers working in different labs and reproduced. Representative data are presented in the manuscript.
Randomization	Mice were allocated into experimental groups using random number assignment to minimize bias and ensure equal distribution across groups. No randomization was applied for cultured cell or in vitro biochemical experiments.
Blinding	For animal studies, including isotope tracing and echocardiography, researchers were blinded to genotype during data collection. The analysis of MS data was conducted by researchers blinded to the genotype and experimental groups. EM data analysis of heart mitochondria was analyzed in a blinded fashion by independent scientists. For in vitro biochemical assays, the researcher designed and performed the experiment individually and was not blinded.

Reporting for specific materials, systems and methods

We require information from authors about some types of materials, experimental systems and methods used in many studies. Here, indicate whether each material, system or method listed is relevant to your study. If you are not sure if a list item applies to your research, read the appropriate section before selecting a response.

Materials & experimental systems

n/a	Involved in the study
☐	☒ Antibodies
☐	☒ Eukaryotic cell lines
☒	☐ Palaeontology and archaeology
☐	☒ Animals and other organisms
☒	☐ Clinical data
☒	☐ Dual use research of concern
☐	☐ Plants

Methods

n/a	Involved in the study
☒	☐ ChIP-seq
☒	☐ Flow cytometry
☒	☐ MRI-based neuroimaging

Antibodies

Antibodies used	1. MPC1: Cell Signaling Technology, Catalog Number: 14462, dilution of 1:1000.<br>2. MPC2: Cell Signaling Technology, Catalog Number: 46141, dilution of 1:1000.<br>3. VDAC: Cell Signaling Technology, Catalog Number: 4866, dilution of 1:5000.<br>4. MCT1: Two sources were used for the MCT1 antibody: Santa Cruz Biotechnology, Catalog Number: sc-365501 (dilution of 1:500), and Proteintech, Catalog Number: 20139-1-AP (dilution of 1:1000).<br>5. LDHA: Proteintech, Catalog Number: 19987-1-AP, dilution of 1:1000.<br>6. LDHB: Proteintech, Catalog Number: 14824-1-AP, dilution of 1:1000.<br>7. COX IV: Cell Signaling Technology, Catalog Number: 4850, dilution of 1:1000.<br>8. ATP5A: Cell Signaling Technology, Catalog Number: 18023, dilution of 1:1000.<br>9. TOM20: Cell Signaling Technology, Catalog Number: 42406, dilution of 1:1000<br>10. ACO2: Cell Signaling Technology, Catalog Number: 6922, dilution of 1:1000<br>11. LC3B: Cell Signaling Technology, Catalog Number: 2775, dilution of 1:1000<br>12. OPA1: Cell Signaling Technology, Catalog Number: 80471, dilution of 1:1000<br>13. TOM70: Santa Cruz Biotechnology, Catalog Number: sc390545, dilution of 1:1000<br>14. Na, K-ATPase: Cell Signaling Technology, Catalog Number: 3010, dilution of 1:1000<br>15. MCT2: Invitrogen, Catalog Number: PA5-88516, dilution 1:1000<br>16. MCT4: Proteintech, Catalog Number: 22787-1-AP, dilution of 1:1000.<br>17. Calreticulin:Cell Signaling Technology, Catalog Number: 2891, dilution of 1:1000<br>18. TIM23:Cell Signaling Technology, Catalog Number: 34822, dilution of 1:1000<br>19. AIF: Cell Signaling Technology, Catalog Number: 4642, dilution of 1:1000<br>20. Citrate Synthase: Cell Signaling Technology, Catalog Number: 14309, dilution of 1:1000<br>21. Alpha-tubulin Cell Signaling Technology, Catalog Number: 3873, dilution of 1:1000<br>22. Viniculin: Cell Signaling Technology, Catalog Number: 13901, dilution of 1:1000<br>23. HA-Tag: Cell Signaling Technology, Catalog Number: 3724, dilution of 1:1000<br>24. Control mouse IgG : Cell Signaling Technology, Catalog Number: 61656.<br>25. Total OXPHOS rodent WB antibody cocktail: Abcam, Catalog Number: ab110413, dilution of 1:1000<br>26. Alpha-tubulin: Proteintech, Catalog number: 11224-1-AP, dilution 1:1000
Validation	To validate the primary antibodies used in this study, we matched banding patterns and apparent molecular weights with relevant peer-reviewed studies and data available from the manufacturer for mouse. MCT1 and MPC1/2 antibodies were validated by laboratory personnel using knockout animal tissue that was confirmed by genetic testing. All antibodies used on human specimens were validated by the manufacturer against human cell lines. Specific antibody validation for mouse use by either reference to peer review study or publically disclosed manufacturer data is listed below:<br>MPC2(Cell Signaling 46141): PMID: 40680845<br>MCT1(Santa Cruz sc-365501): PMID: 36107481

MCT4 (ProteinTech 22787-1-AP): PMID: 41721736
MCT1 (ProteinTech 20139-1-AP): PMID: 41721736
COXIV (Cell Signaling 4850): PMID: 41199017
TOM20 (Cell Signaling 42406): PMID: 40742280
Calreticulin (Cell Signaling 2891): PMID: 40108405
VDAC (Cell Signaling 4866): validated in mouse cell line C2C12:https://www.cellsignal.com/products/primary-antibodies/vdac-antibody/4866
LDHA (ProteinTech 19987-1-AP): validated in mouse cell line NIH/3T3: https://www.ptglab.com/products/LDHA-Specific-Antibody-19987-1-AP.htm
LHDB (ProteinTech 14824-1-AP): validated in mouse heart: https://www.ptglab.com/products/LDHB-Antibody-14824-1-AP.htm
ATP5A (Cell Signaling 18023): validated in mouse cell line Neuro2A: https://www.cellsignal.com/products/primary-antibodies/atp5a1-antibody/18023
ACO2 (Cell Signaling 6922): validated in mouse cell line C2C12: https://www.cellsignal.com/products/primary-antibodies/aco2-antibody/6922
LC3B (Cell Signaling 2775):validated in mouse cell line A20: https://www.cellsignal.com/products/primary-antibodies/lc3b-antibody/2775
OPA1 (Cell Signaling 80471):validated in mouse cell line C2C12: https://www.cellsignal.com/products/primary-antibodies/opa1-d6u6n-rabbit-monoclonal-antibody/80471
TOM70 (Sant Cruz sc-390545): validated in mouse cell line MTE1D: https://www.scbt.com/p/tom70-antibody-a-8
Na,K-ATPase (Cell Signaling 3010): validated in mouse cell line C2C12: https://www.cellsignal.com/products/primary-antibodies/na-k-atpase-antibody/3010
Alpha-tubulin (Cell Signaling 3873): Validated in mouse cell line C2C12 https://www.cellsignal.com/products/primary-antibodies/alpha-tubulin-dm1a-mouse-monoclonal-antibody/3873
TIM23:(Cell Signaling 34822):validated in mouse cell line C2C12:https://www.cellsignal.com/products/primary-antibodies/tim23-e1q7l-rabbit-monoclonal-antibody/34822
AIF: (Cell Signaling 4642: validated in mouse cell line NIH/3T3: https://www.cellsignal.com/products/primary-antibodies/aif-antibody/4642
Citrate Synthase (Cell Signaling 14309): validated in mouse cell line C2C12:https://www.cellsignal.com/products/primary-antibodies/citrate-synthase-d7v8b-rabbit-monoclonal-antibody/14309
Total OXPHOS rodent WB antibody cocktail (Abcam ab110413): optimized and validated in mouse and rat tissue extracts: https://www.abcam.com/en-us/products/panels/total-oxphos-rodent-wb-antibody-cocktail-ab110413
MCT2 (Invitrogen PA5-88516): validated in human cell line 293T: https://www.thermofisher.com/antibody/product/MCT2-Antibody-Polyclonal/PA5-88516
HA-Tag (Cell Signaling 3724): validated by manufacturer: https://www.cellsignal.com/products/primary-antibodies/ha-tag-c29f4-rabbit-monoclonal-antibody/3724?srsltid=AfmBOoolIXwG6U5aHFc2sFj69qx3HYUNPx4IJG_GxBgdqAbAbX3Ft8KK
Control mouse IgG: Cell Signaling 61656): Validated in human cell lines: https://www.cellsignal.com/products/primary-antibodies/ha-tag-c29f4-rabbit-monoclonal-antibody/3724
Vinculin: Cell Signaling 13901: Validated on human cell lines: https://www.cellsignal.com/products/primary-antibodies/vinculin-e1e9v-rabbit-monoclonal-antibody/13901
Alpha-tubulin (Proteintech 11224-1ap): validated on mouse heart: https://www.ptglab.com/products/TUBA1B-Antibody-11224-1-AP.htm

Eukaryotic cell lines

Policy information about cell lines and Sex and Gender in Research

Cell line source(s)	HEK293T cells. Human female embryonic kidney derived cell
Authentication	Cells were freshly purchased from ATCC (Prod. number CRL-3216)
Mycoplasma contamination	Cells were negative for mycoplasma during routine laboratory testing
Commonly misidentified lines (See ICLAC register)	No commonly misidentified cell lines were used in this study.

Animals and other research organisms

Policy information about studies involving animals; ARRIVE guidelines recommended for reporting animal research, and Sex and Gender in Research

Laboratory animals

Mus Musculus, C57BL/6J mice, 8-12 weeks old for the beginning of most experiments. Specific experiments as noted were carried out in older mice (1 year of age). Mice were housed under a standard 12:12 light:dark cycle and lights were turned on at 6:00 am MST. Mice were given ad libitum access to food and water. All mice were fed a standard rodent chow diet (Teklad 2920). Mice were housed in a facility within a temperature range of 20-26C (average 23C) at an altitude of 1520m. Humidity was not specifically controlled for due to facility engineering constraints and could vary from 30-70% over the course of the year depending upon ambient weather conditions. During awake stable isotope tracing experiments, food was restricted 3 hours prior to and during the experiment. Mouse strains used in this study were constructed on the C57BL/6J background. Specific strains used in this study are: MCT1fl/fl; MPC1fl/fl;MCT4fl/fl; aMHC-MerCreMer. Tamoxifen-inducible CRE knockout animals were generated by crossing floxed

	allele animals with aMHC-MerCreMer. All crosses yielded litters at expected mendelian ratios
Wild animals	No wild animals were used in the study.
Reporting on sex	All studies used male and female animals in roughly equal proportions as resulting from our breeding strategy. For biochemical characterizations, we did not power the sample sizes to detect sex differences. For functional echocardiography studies, we have analyzed our data for sex differences and did not determine anything significant with the sample sizes used. Thus, for the purpose of this research project we have combined sexes for our analysis.
Field-collected samples	No field collected samples were used in the study.
Ethics oversight	All procedures were approved by the Institutional Animal Care and Use Committee (IACUC) of the University of Utah.

Note that full information on the approval of the study protocol must also be provided in the manuscript.

Plants

Seed stocks	n/a
Novel plant genotypes	n/a
Authentication	n/a
