# Supplementary material for: MCT1-dependent mitochondrial lactate import sustains cardiac metabolism
Source: Nature. 2026 Oct 28;659(8140):523–31. doi: 10.1038/s41586-026-11182-3 (PMC13754610; doi:10.1038/s41586-026-11182-3)
Supplement: Supplementary file 3 [file 41586_2026_11182_MOESM7_ESM.pdf]

nature portfolio

Peer Review File

MCT1-dependent mitochondrial lactate import sustains cardiac metabolism

Corresponding Author: Dr Gregory Ducker

Any redactions in this file are there to maintain patient confidentiality, the confidentiality of unpublished data, or to remove third-party material.

This file contains all reviewer reports in order by version, followed by all author rebuttals in order by version.

Version 1:

Reviewer comments:

Referee #1

(Remarks to the Author)
In this manuscript the authors provide evidence that lactate is directly metabolized by cardiac mitochondria and that mitochondrial lactate uptake is mediated by the mitochondrially localized monocarboxylate carrier (MCT1). The authors use extensive tracer labeling experiments in animals perfused in vivo and in isolated mitochondria to support the existence of functional lactate metabolism. A cardiomyocyte-specific MCT1 KO mouse is generated and in addition to impaired contribution of lactate derived carbons to TCA cycle intermediates, these animals reveal impaired resilience when subjected to diverse hemodynamic stressors including pressure overload hypertrophy and ischemia/reperfusion. The experiments presented are generally well conducted. However, there are some gaps in the data presented that limit the strength of some of the authors assertions.

Specific Critiques
1. The authors begin by studying mice with inducible KO of the mitochondrial pyruvate carrier (MpcKO). The authors state that studies were performed in MpcKO mice at a time when these hearts exhibited normal contractile function. It is not transparently indicated how many weeks after Mpc KO were the studies performed. Moreover, it has been previously shown by several groups, including the authors, that Mpc KO mice spontaneously develop heart failure as a function of duration of loss of the pyruvate carrier. As such, it is essential that the authors also analyze Mpc KO animals at a time that they are failing, to determine if the failure of these hearts correlates with loss of an ability to metabolize lactate (or something else).

2. A fundamental question that is not addressed by this study is: Why do unstressed Mpc KO mice spontaneously fail, whereas MCT1 KO mice do not? Preserved lactate oxidation (if true) does not appear to be sufficient to prevent them from failing. Do Mpc KO mice lose MCT1 expression as they fail, or is there some other mechanism that prevents lactate utilization from "rescuing" Mpc KO hearts are they age? Does lactate oxidation also fall in Mpc Ko hearts as they fail? Thus, it will be important for certain key experiments (including substrate tracing) to done in both models in parallel, in stressed and non-stressed hearts.

3. Although the authors show extensive data that MCT1 KO animals fail in response to various hemodynamic stressors, this is also true of Mpc KO mice as well. Therefore, both models demonstrate that substrate switching between lactate and glucose is not sufficient to maintain cardiac function. The story would therefore benefit by substrate tracing in both sets of mutant animals under conditions of hemodynamic stress, to more comprehensively address this important issue.

4. Moreover, it appears that angiotensin/PE treatment leads to reduced expression of MPC, thus is the LV dysfunction that develops following Ang/PE largely a function of MPC loss, which is further exacerbated in MCT1 KO mice? Could these animals be rescued by maintaining MPC expression? Furthermore, seeing that MPC levels decline in Ang/PE controls that do not appear impact LV functon(figure 4C) and impacts LV function to a smaller degree after TAC (Figure 4D), then would MpcKO mice have the same relatively more benign phenotype as seen in Ang/PE , TAC-treated or MI treated WT mice? Based on prior literature this might be unlikely. What happens to Mpc and Mct1 protein levels in hearts after TAC?

5. In the ischemia model, control animals regain function after 6-7 weeks, but MCT1 KO mice do not. In this model, what

happens to MPC expression in KO and control cohorts? Do MPC KO mice, that also maladapt in response to hemodynamic stress behave any differently?

6. Although many experiments are performed I non-beating adult cardiomyocytes to indicate that these cells metabolize both lactate and glucose-derived pyruvate via distinct pathways, this approach does not address what happens to relative substrate utilization in intact beating heart preparations, which would be more physiologically relevant.

7. It is not clear how lactate mediated respirations remain normal in CPI-613 treated cells, in which PDH and -KGDH is inhibited. Is this “normal” oxygen consumption coupled to ATP generation?

8. The authors show that mitochondrial MCT1 is increased in failing human hearts, but it is not clear what this actually means. If their thesis is that lactate utilization may be an adaptation that could potentially preserve cardiac function in failing hearts, then it is not apparently of benefit in this circumstance. In another part of the paper, the authors actually measured mitochondrial respirations in human heart derived mitochondria. The experiments were presumably designed to show that human mitochondria can respire on lactate. It is not clear why experiments were not done on mitochondria from failing hearts to determine if the increase in MCT1 correlated with increased lactate utilization. The authors also have access to cardiac tissue from LVAD samples of individuals whose LV function recovered. What happens to MCT1 content in this context? In the absence of these analyses no conclusions can be drawn from the observation that MCT1 protein is increased in failing human heart mitochondria.

9. Immunohistochemistry of MCT expression in cardiomyocytes is of low quality.

10. Figure 3H shows NAD enrichment from labeled lactate. What is happening to the total NAD pool (labeled and unlabeled in these experiments)? Similarly for figure 3I to be fully interpreted NAD+ and the NADH/NAD+ ratio is necessary to be shown.

11. Because MCT1 is expressed in the plasma membrane and the mitochondria the authors should acknowledge that this imposes some limitation on their interpretation of in vivo studies in the MCT1KO model, because of the inability to distinguish between effects that could be due to cytosolic versus mitochondrial lactate abundance and metabolism.

12. It appears that the major adaptation in MPC1 KO animals is increased lipid oxidation. Why is fractional lactate use not increase in MPC KO hearts despite a clearly and substantially increased expression of MCT1?

Minor Issues
1. Please show the actual concentrations of metabolites in HPLM
2. Extended Figure 5 panels b-e: Figure would be easier to understand if a line was drawn about panels b, c, d,e to indicate that these are being supplied with labeled lactate. Please clarify the differences between data shown in l,m and data shown in n,o.

Referee #2

(Remarks to the Author)
The manuscript presented by Cluntun et al presents a novel finding that lactate can directly enter cardiomyocyte mitochondria in a non MPC-dependent manner. They suggest that lactate entry into mitochondria occurs via MCT1 as mitochondrial lactate entry is significantly reduced when MCT1 is absent or inhibited. Using both in-vivo and ex-vivo isotopic tracing methods, they present compelling evidence to show that lactate can be directly oxidized within mitochondria thus generating an intra-mitochondrial pool of lactate-derived NADH. They go on to demonstrate that mice lacking cardiomyocyte-specific MCT1 are more susceptible to heart failure following neurohormonal and ischemic insults, suggesting that MCT1 plays a biologically meaningful role in cardiac adaptation to physiologic stressors.

The question of whether lactate can enter and undergo oxidation within mitochondria was proposed many years ago and has been highly controversial. The authors present a compelling argument that this is in fact true, at least in cardiac mitochondria. The meat of the matter is figure 3, demonstrating lactate oxidation in intact mitochondria. The work is important; however, several additional experiments are needed to support physiological relevance and explain certain paradoxical findings.

1. Overall, the impact of the paper could be significantly strengthened if the authors were to test the role of mitochondrial MCT1 without impacting plasma membrane MCT1 activity. In the absence of such an experiment, it is impossible to discern whether cardiac MCT1 KO predisposes to heart failure due to loss of plasma membrane or mitochondrial function. For instance, if MCT1 has a mitochondrial localization sequence, such an experiment could involve deletion of the localization sequence and testing lactate oxidation/respiration in isolated cardiomyocytes and, if feasible, response physiologic response to cardiac injury. Alternatively, one could delete MCT1 and then reintroduce PM-targeted vs mito-targeted MCT1.

2. Figure 1 redemonstrates the fate of glucose in the MPC1 cardiac KO as originally reported in Y. Zhang, ED Abel et al Nature Metabolism 2020. The original study is not cited in this section of the manuscript though it should be acknowledged. Here the authors recapitulate most of the original findings, including two perplexing observations that should be explored experimentally.

a. First, there is a striking increase in 13C-glucose derived enrichment of myocardial lactate and pyruvate that is not explained. Could this be due to suppressed cardiac glycogen breakdown in the MPC1 CKO (Figure 1c)?
b. Second, there is significantly increased 13C-glucose contribution to succinate and malate in the MPC1 cKO that is striking though not discussed (Figure 1c). How do the authors explain this observation? This finding is especially intriguing given that the authors find less, not more, lactate contribution to TCA cycle in the MPC1 KO condition (Extended Data 1m), implying that the increased 13C label in the TCA cycle in MPC KO is not coming from lactate.
c. Finally, the authors find here a trend towards less 3-HB contribution to succinate and malate in MPC CKO, which is quite a contrast from the 2-fold increase in ketone-derived contribution to TCA in Zhang et al. This may be due to different ketone concentrations found in plasma (here) vs. perfusion (Zhang 2020). Given that ketogenic diet rescues HF in MPC1 CKO, the contrasting finding between studies merits discussion.

3. Figure 2 has several major weaknesses.
a. First, the colocalization images (2a-d) are of insufficient resolution to support the claim that MCT1 localizes to the IMM as opposed to the OMM. The distance between the inner and outer mitochondrial membranes is ~20 nm, whereas the scale bar is 5-10 microns. Additionally, there is no quantification of images. And it appears graphs are taken selectively from one area per panel.
b. Second, the proteinase K protection assay is poorly described and also lacks quantification, or being done more than once. The authors should describe the swelling condition and what is meant to be concluded from the swelling intervention.
c. Finally, the figure title ("Lactate oxidation requires MCT1") is not justified given that there is substantial residual lactate-derived 13C contribution to succinate and malate in MCT1 iCKO hearts. In fact, it appears there is equivalent if not more 13C-lactate derived enrichment of succinate/malate in Mct1 iCKO than there is 13C-glucose-derived enrichment of succinate/malate in WT animals. As a minor point, the y-axis of Figure 2g should be corrected to "13C enrichment" or "13C labeling contribution" as it appears the data reflect enrichment and not total metabolite abundance.

4. Figure 3 demonstrates technical feasibility for (1) lactate to enter cardiac mitochondria and (2) for lactate oxidation to directly produce NADH outside of the TCA cycle.
a. While striking, the conclusion of the figure would be better supported by additional experiments to support physiologic relevance. The data demonstrate that lactate CAN enter mitochondria directly and be oxidized, but the important physiological question is DOES lactate do this under normal circumstances, or is this an artifact of the in vitro conditions? The authors could consider repeating the 2-2H lactate infusion with NAD2H quantification in an isolated (i.e. langendorff) heart model, and quantify ACM and mitochondrial NAD2H, which would support the physiologic relevance of mitochondrial lactate observation.
b. In Figure 3k, there is a surprising finding that merits discussion: it is not clear here why respiration is not suppressed by CPI-613. The pyruvate produced by LDHB should still be oxidized within the TCA cycle and thus respiration should be suppressed with CPI-613. Why is this not the case?

5. Figure 4 shows that loss of cardiac MCT1 increases susceptibility to HF following AngII/PE treatment or ischemia/reperfusion injury.
a. While the phenotype is striking, it is not clear if the phenotype is due to lack of MCT1 at the plasma membrane, mitochondrial IMM, or both (see comment above). Additionally, given that MCT1 also contributes to mitochondrial ketone uptake, it is conceivable that MCT1 loss increases HF susceptibility due to defective ketone oxidation in addition to or instead of defective MCT1-mediated lactate uptake. 13C-hydroxybutyrate infusion (either isolated heart or in-vivo) would help to clarify this last question.
b. Finally, there is an interesting contrast between MCT1 CKO and MCT1 skeletal muscle KO as reported in L Zhang, Y Chen et al Science Advances 2024. Skeletal muscle MCT1 KO leads to increased PGC1a level, increased mitochondrial density, and increased running distance. Do similar changes (i.e. increased PGC1a and increased mitochondrial density) happen in the heart in cardiac MCT1 KO? If so, it is conceivable that increased mitochondrial density is detrimental in the setting of hypoxic stress such as ischemia/reperfusion injury.

Referee #3

(Remarks to the Author)
Cluntun et al. propose that lactate is directly imported into myocardial mitochondria and subsequently oxidized to generate NADH and pyruvate, thus supporting the TCA cycle/mitochondrial functions. Using isotope tracing and genetic mouse models, this study starts to address ongoing debate in the field regarding the mitochondrial lactate oxidation. In agreement with prior findings from George Brooks et al., the authors also confirm that monocarboxylate transporter 1 (MCT1) is localized in the mitochondria and facilitates the import of lactate. Furthermore, they show that MCT1 loss leads to rapid progression to heart failure, along with compromised myocardial structure and function after injury.
While this study is intriguing, there are some concerns as below.

1) The study aligns with previous reports suggesting MCT1 is located in the mitochondrial membrane. However, one major concern is the purity of the mitochondrial preparations (e.g. Fig. 2G). Since MCT1 is expected to be predominantly in the plasma membrane, it would be useful to show both plasma membrane and cytosolic markers in the mitochondrial fraction. Furthermore, clarifying the proportion of MCT1 present in mitochondria versus the plasma membrane would provide some

further insight for data interpretation. It would also be useful to report whether other MCT family members, such as MCT4, are present in the mitochondrial fractions.
Mitochondrial purity could also be confirmed through proteomics analysis. Additionally, the microscopy images in Fig. 2A-D do not provide clear evidence of mitochondrial localization of MCT1; higher-resolution/magnification images would better support these findings.

2) The evidence for "Direct lactate import assays" is not convincing. While Fig. 3i and Extended Data Fig. 5f-j suggest that lactate oxidation is impaired in purified mitochondria, these results could potentially be explained by cytosolic contamination of LDH in the mitochondrial preparations, which can generate NADH and pyruvate in the cytosol, wherein pyruvate could be imported by MPC. To strengthen these findings, it would be valuable to repeat Fig. 3i assay in a double knockout model of MPC/MCT1.

3) At a minimum, it would be important to conduct mitochondrial uptake assays for lactate and pyruvate in a time-course fashion in both MCT1 and MPC1 knockout cardiomyocytes.

4) Using an LDH inhibitor in purified mitochondria, the authors propose that LDH activity may be present within the mitochondria and required for lactate oxidation. However, the purity of the mitochondrial preparation remains a concern, as LDH is highly abundant and could contaminate mitochondrial fractions. Clarifying the mitochondrial origin of LDH is essential, as well as identifying the specific LDH isoform(s) involved. To address the conflicting data in the field (PMID: 31610469; PMID: 30939431), both functional and genetic validation of mitochondrial LDH localization and activity are warranted.

5) Fig. 2j. It is surprising that the loss of MCT1, a well-known plasma membrane lactate importer, does not alter cellular lactate levels compared to the control. Could other MCT transporters be compensating for lactate import? A time-course assay assessing whole-cell lactate uptake in cardiomyocytes/infusions (Fig. 2j) would be important here to help determine if any differences in lactate uptake appear at earlier time points.

6) The hepatic half of Cori-cycle consumes lactate for gluconeogenesis. Is the mitochondrial localization of MCT1 specific to cardiac tissues, or is it also present in hepatic tissue? Similarly, is mitochondrial lactate oxidation unique to the heart, or does it happen in other tissues or cell types?

7) Extended Fig. 1c shows that lactate infusion leads to the production of M+3 glucose, indicating that gluconeogenesis is taking place. While the authors propose that cardiac lactate oxidation depends on MCT1, it remains unclear which of the two pathways, MPC or MCT, is required for directing pyruvate toward oxaloacetate and, subsequently, gluconeogenesis.

8) In Fig. 2e, what do the authors mean by “swelling”? This is not explained in the methods or figure legends. Additionally, the quality of the blots is suboptimal, and quantification would be helpful.

9) Confirming the expression levels of other MCT, MPC, and LDH isoforms in the presented knockouts, both at whole-cell and mitochondrial fractions, would provide useful insight and controls. Is there potential compensation by MPC2 in the absence of MPC1?

Version 3:

Reviewer comments:

Referee #1

(Remarks to the Author)
Critiques
The authors conducted additional experiments to address concerns raised by review or the original submission. Their data provide evidence in favor of a model in which cardiac mitochondria may directly metabolize lactate. Unfortunately, the data quality, particularly for the mitochondrial fractionation studies are variable and inconsistent and degrade confidence in the localization of MCT1 primarily in the IMM. There are also inconsistency in some of the tracing studies. The mechanisms for increased susceptibility of MCT1 deficient hearts to greater dysfunction in response to hemodynamic stress remain to be fully clarified. Concerns with the revised manuscript that support this evaluation are summarized below. The critiques are structured to provide either the figure number or statement/data followed by the concern.

Figure 1d: Given that there is equivalent serum labeling of glucose and lactate whether U13 Glucose or Lactate is infused, it is difficult to understand why TCA labeling would be reduced. i.e. why are (c) and (d) different.

Figure 1h and 1i: Failing MPC hearts reveal significant attenuation of C13 labeling in glucose infused animals. This is attenuated in lactate perfused animals despite equivalent levels of serum C13 enrichment. What is the basis for this? Decreased MCT1 in failing MPC1 KO hearts?

Figure 1j: A single lane is shown for each genotype. WIthoug quantification of multipke samples it is not possible to conclude if MCT1 is reduced in the ehart of failing MPC1cKO

Figure 2a: C13 lactate in cardiac tissue increased only in WT mice after AZD treatment. Unclear why this is not seen in AZD treated MPC1 KO animals, which cannot oxidize lactate or pyruvate. Relative to what is shown in Figure 1c pyruvate enrichment in MPC1KO hearts seems attenuated, although this could reflect a smaller sample size. It is surprising though that AZD treatment does not increase pyruvate enrichment in MPC1KO hearts.

Figure 2a: AZD infusion would block all CHO oxidation in MPC1 hearts. The duration of AZD treatment is not stated. Moreover, it is important to know what the impact of AZD treatment on LV function is, particularly given the reduction in TCA cycle labeling. If LV function declined significantly, this would confound interpretation of the data.

Figure 2f: In MCT1 KO adult cardiomyocytes incubated with 13C Lactate the authors comment on reduced TCA intermediate labeling but are silent on the reduction in lactate and pyruvate labeling. Why would these be reduced? Could this be due to reduced uptake of lactate?

Figure 2g and 2h: Seeing that glucose and lactate are both labeled when 13C glucose or 13C lactate is infused and given the lack of difference in serum labeling under either condition it is not clear why reduced TCA cycle labeling is only seen when lactate is infused, but not when glucose is infused. These are similar to concerns raised for Figure 1d.

Figure 2k: This figure provides the most convincing data of reduced TCA cycle intermediates in perfused hearts exposed solely to lactate. However, the pattern for lactate and pyruvate labeling are different, when compared to figure 2f in isolated cells, where these are markedly reduced. What is the basis for this discrepancy?

Statement line 174-176: “We noted high and unchanging myocardial expression of the lactate transporters MCT2 and MCT4 in all genotypes without altered subcellular localization (Extended Data Fig. 4a)": CRITIQUE: This statement is not true. MCT4 data is only shown in whole hearts for floxed controls but not in MPC1KO or in MCT1KO, There is no evidence for MCT4 expression in the 2 KO samples. Why is whole heart data or membrane preps not shown for these genotypes?

Statement line 174-176: “When comparing Mpc1iCKO, Mct1iCKO and control hearts from young mice, we observed only modest changes in cytosolic and mitochondrial lactate or pyruvate metabolismproteins, suggesting a high degree of flexibility in cardiac metabolism in unstressed animals". CRITIQUE: This is an overstatement. The authors cannot make any conclusions about substrate flexibility on the basis of protein levels.

Statement line 178-180: “However, we noted increased mitochondrial localization of MCT1 in Mpc1iCKO 179 hearts (Fig. 2c and180 Extended Data Fig. 4a)”. CRITIQUE: Western blot data should be quantified for a valid conclusion to be made about MPC levels in MCT KO hearts.

Statement line 192-194: “Importantly, loss of MCT1 did not alter tissue lactate or pyruvate enrichment, suggesting that the observed TCA cycle defects were not due to a loss of cellular lactate import.” CRITIQUE: The data in ACMS (figure 2f) would argue against this. Moreover, the authors’ conclusion here does not rule out cytosolic conversion, even if lactate import was reduced.

Figure 3a and 3b: MCT1 is reduced both in ISO and Hypo following PK treatment. This suggests the possibility that there is MCT1 contamination from elsewhere in these preparations. This therefore casts some doubt on the validity of the proteomics data shown in extended figure 4.

Figure 3a: Why is MPC1 not shown?
Figure 3b: Why are OPA1 and MPC1 not subject to proteinase K in triton treated samples?
Figure 3b: TX100 (PK -) increases MCT1 immunoreactivity in mouse to a large extent but NOT in humans.
Figure 3d: Why is MCT not shown in the MITO-Tag IP?
Figure 3d: The amount of LDHB that is pulled down appears trace.

Figure 3l: What would happen to lactate driven mitochondrial O2 consumption when MCTi and LDHi are combined?

Statement line 220-223: “In these preparations, we also observed a protected LDHB band that showed similar digestion patterns to citrate synthase, a matrix protein. Similar protection patterns were observed in mitochondria from a human cell line, failing human hearts, and non-failing human donor hearts (Fig. 3b and Extended Data Fig. 5j,k)." CRITIQUE This statement obfuscates many problems with all the mitochondria fractionation data, as summarized below.

Extended data Figure 5f: Why are OPA1 and MCT1 reduced in ISO + PK?
Extended data Figure 5f: Why is LDHB not proteinase K protected under hypotonic conditions? This questions adequacy of the preps.
Extended data Figure 5f: MPC1 is not uniformly subject to PK hydrolysis even after triton.
Extended data Figure 5f, 5i,5k: Why is ACO2 resistant to PK degradation?
Extended data Figure 5g and 5h: MCT1 is markedly reduced following PK treatment under iso and hypo conditions. This suggests contamination with non-mitochondrial MCT1, thereby questioning the validity of the author’s conclusions.

Extended data Figure 5i : In this sample MCT1 is protected from PK under ISO conditions, which contrasts with 5g and 5h. Data are inconsistent. MCT1 is protected under hypo conditions. This undermines authors conclusions that MCT is an IMM protein.

Extended data figure 5j: Why is MCT1 absent in the TX100 samples without PK?
Extended data Figure 5j: Some LDHb is reduced under hypo conditions±PK
Extended data Figure 5K: These blots are largely uninterpretable. MPC1 and LDHb do not appear to be subject to PK degradation even under TX100 conditions.

Statement line 236-237: "However, in the mitochondrial fraction, 2H lactate was absent from Mct1iCKO samples (Fig. 3e and Extended Data Fig. 6d)." CRITIQUE: Extended Data Fig. 6 is an immunoblot.

Extended data Figure 6d: Why have the authors not blotted for MCT1 in the Mito-Tag pull down samples? This omission calls the data into question.

Extended data Figure 8j: Please clarify if these data Showing reduced MPC1 were obtained from mice that were treated with ANG/PE as opposed to cells that were cultured with ANG/PE

Figure 4f and 4g: How are these MPC QPCR reconciled with the data in extended Figure 8j showing reduced MPC1 expression? Moreover, MPC protein seems much higher in MCT1KO mitochondria. How do the authors reconcile these disparate data?

Figure 4i: The increased NADH following ANG/PE in MCT1 KO animals could reflect increased conversion of lactate to pyruvate. Increased NADH could also correlate with increased TCA cycle flux, but this would not correlate with decreased ATP.

Statement Line: 315-317 “Upon Ang/PE treatment, metabolite changes in Mct1iCKO hearts were of greater magnitude and NADH was the most upregulated metabolite,suggestive of impaired oxidation in these animals (Fig. 4i).” CRITIQUE: This statement is simplistic. See comment in Figure 4i above.

Extended data Figure 9i : The authors only show MPC1 QPCR data from sham and TAC WT mice. Why is data from not being shown from MCT1 Sham and TAC mice? Conclusions cannot be made without these data. Why are the authors not showing MPC protein levels by Western Blot?

Extended data Figure 9m/n: Why is QPCR data from sham and TAC KO mice not shown for slc16a1 and slc16a2? Why are protein levels by western blot not shown?

Extended data Figure 10 a/b: Metabolomics data do not really shed any additional mechanistic insight. They could simply represent the differences between varying degrees of heart failure

Extended data Figure 10d: TCA cycle intermediate C13 enrichment in lactate infused mice falls in A/P treated controls as well as A/P treated MCT1 KO mice. Thus the authors cannot conclude that this proves a specific defect attributable to MCT1 loss, given that MPC1 is also repressed in these both groups of animals as well.

Extended data Figure 10h and 10i: Cristae number and area decline to the same extent following ANG/PE in control and PCT1 KO mice. Thus the authors cannot conclude that this phenomena correlates with the worse HF in this model.

Extended data Figure 11n: Protein data is needed to bolster QPCR

Statement line 329-332: “In contrast, lactate’s contribution to the TCA cycle (malate, succinate and glutamate) was attenuated in hearts from Mct1iCKO treated animals with Ang/PE compared to saline treated controls and Mct1fl/fl Ang/PE animals (Fig. 4j and Extended Data Fig. 10d)." CRITIQUE: This conclusion does not hold up when one examines each TCA cycle intermediate independently. The authors also do not acknowledge that ANG/PE in WT mice also reduces labeling of these TCA intermediates.

Statement line 356-358: “The MCT1-dependent changes in ROS are congruent with our mitochondrial ultrastructure data and suggest a role for MCT1 in the core structure and function of mitochondria which become pathogenic upon cellular damage and energetic stress." CRITIQUE: What is the basis for the oxidative stress? To interpret these experiments the authors need to measure oxygen consumption. What is buffer condition under which these experiments were performed? Moreover, MCT1 deficient cells are starting off with higher baseline ROS anyway. And this increase in baseline seems to correlate with marginal changes in mitochondrial structure in untreated mice. These data support the notion that impaired lactate uptake predisposes to mitochondrial dysfunction; however, the mechanisms remain to be determined.

Statement line 366-368: “In the failing hearts of Mct1iCKO mice, expression of Lhda and Ldhb transcripts was significantly increased, and Mpc1 was decreased, indicating glucose andlactate metabolic pathway stress which was independent of Pgc1α (unchanged expression)" CRITIQUE: This statement represents an over-interpretation of the data.

Statement line 369-372: “Finally, analysis of mitochondria from patients with advanced HF with reduced ejection fraction and non-failing donor controls revealed increased mitochondrial MCT1 localization without a statistically significant change in

total MCT1 or MPC protein levels (Fig. 4p, Extended Data Fig. 12a-e)". CRITIQUE: The authors have not shown mitochondrial localization. A more accurate summary of these data is that MCT1 is increased in mitochondrial fractions but not whole heart homogenates in human HF samples.

MINOR COMMENTS
Figure 1a - The lactate to pyruvate interconversion should have an arrow on each end.
Figure 1b - Normalize vs. Normalized?
Figure 2c, Extended Figure 4a: Is MPC1 KO partial?
Line 1031: Change “inhibit” to “inhibitor”
Figure 3a, Extended data figure 5 OPA Blots are low quality

Referee #2

(Remarks to the Author)
The authors have extensively addressed my comments. I have a few comments/responses back, all relatively minor:

The authors’ response to comment 2a is unlikely to be the right answer. Glycolysis is super fast (seconds) and almost certainly at labeling equilibrium. Changes in relative enrichment are thus unlikely to reflect flux, but rather relative contributions to the pool. Higher labeling from external glucose likely reflects less “non-labeling” from internal sources (most likely glycogen). If glycogen pools are unchanged, as stated by the authors, it is possible that glycogen turnover is accelerated, and thus carrying more label, which can be tested by quantifying labeling of the glycogen pool itself. Another possible explanation would involve compartmentalization of fluxes.

The authors’ response to comment 4b is a reasonable explanation that is easily tested: does inhibition of MPC increase lactate-to-TCA oxidation and thus respiration, and now CPI-613 would decrease respiration?

the authors’ response to comment 5a is appreciated, and yes, I would include these data in the manuscript, it is an important caveat. The effect is likely not statistically significant because n= only 3-4 / group. i.e., statistically speaking, absence of significant difference does not prove equivalence.

Referee #3

(Remarks to the Author)
The newly added data substantially strengthen the manuscript, and the authors now provide compelling evidence that mitochondrial lactate metabolism is dependent on MCT1. However, the central claim, direct mitochondrial lactate import, remains insufficiently supported. While the functional, genetic, and localization data strongly suggest a role for MCT1 in enabling lactate oxidation in mitochondria, the manuscript does not provide direct evidence of lactate transport across the mitochondrial membrane. In the absence of a transport assay or an equivalent direct measurement of mitochondrial lactate uptake, the title’s claim “Direct mitochondrial lactate import enables cardiac metabolic flexibility under stress” overstates what the data demonstrate. Additional direct transport evidence is needed to fully support this claim.

Version 5:

Reviewer comments:

Referee #1

(Remarks to the Author)
The authors present a large volume of data to make the case that mitochondria can import lactate directly and that this mediated (in part) by MCT1 that is localized to mitochondria. That mitochondria import lactate is not in question, nor that it could be mediated in part by Mct1. Given the large volume of data presented, it is important that the manuscript and all the data presented are evaluated rigorously. The data generally support the author’s hypothesis and contribute an important perspective to the field. That said, there are a number of inconsistencies in the data, many of which were pointed out in the prior review and to which the authors have chosen not to respond. Two concerning examples (among others) are summarized below.

• Within the manuscript the authors do not replicate their own data. If you closely examine extended data figure 11 (d) you will observe that no difference in 13C labeling between saline (sham) treated Mct1fl/fl controls and similarly treated Mct1cko mice is seen. This contrasts with the data in Figure 2H, where they show reduced labeling of succinate and malate.
• Figure 4J shows a strong trend towards increased 13C labeling in lactate infused saline (sham) treated Mct1cko hearts relative to Mct1fl/fl controls. This contrasts with data shown in Figure 2(h) and extended data figure 5 (b).

As pointed out in the prior review the authors make statements that partially represent the data. For example, they note in the

text that Mpc mRNA is reduced in stressed Mct1cko hearts but fail to systematically acknowledge the protein data, which per the data shown in Figure 4g, appears to be increased at least in one model, and not shown for others. Of course, the authors do not actually quantify this or many other western blots (unclear how many replicates), so readers cannot be certain what the actual changes in protein levels might be. Similarly, relatively small changes in Mpc1 expression are alluded to in their descriptions of models of stressed Mct1cko hearts, which are meaningless in the absence of protein data.

This reviewer will not recite again the multiple inconsistencies previously noted in the proteinase K protection assays that remain un-addressed in this revision.

There are relatively straightforward things that the authors could do to increase confidence in their findings such as showing MCT protein in their mito-tag studies and addressing seeming discrepancies in relative abundance of MCT in mitochondria vs non-mitochondrial fractions (compare figure 1j, 1k, 2c and 4g). Curious why plasma membrane Mct1 is not shown in their KO models, where it would be more abundant.

Data interpretation - The authors state: “Upon Ang/PE treatment, metabolite changes inMct1iCKO hearts were of greater magnitude and NADH was the most upregulated metabolite, suggestive of increased lactate and impaired oxidation in these animals (Fig. 4i).” A more likely explanation for the increase in NADH in stressed Mct1cko hearts is likely increased glycolysis coupled with impaired oxidative phosphorylation.

Other data are curious and not interpreted or addressed: e.g. potential reasons for lactate /pyruvate labeling not being increased in AZD treated Mpc1Ko animals.

Referee #2

(Remarks to the Author)
I find that the authors have provided overwhelming evidence in support of their thesis. Could more studies be carried out? Of course, always. Maybe, for example, studies with reconstituted liposomes of mitochondrial membranes (perhaps what Rev3 is suggesting). However, such studies are not trivial and may not work technically, and as outlined in the authors response to Rev3, at this point there is little alternative interpretation to the overwhelming data provided than that cardiac lactate oxidation requires MCT1-mediated direct mitochondrial lactate import. I would argue that it is time to let the field interpret these compelling and important data.

Referee #3

(Remarks to the Author)
I thank the authors for the additional work performed during this revision, which I recognize is technically demanding in cardiomyocytes. I also appreciate that direct demonstration of mitochondrial lactate import is genuinely difficult.
The new genetic, pharmacological, localization, and permeabilization data support the conclusion that cardiac lactate oxidation requires MCT1, and that the MCT1-dependent step occurs downstream of plasma membrane transport. The saponin experiments, in particular, address my prior concern about distinguishing plasma membrane from mitochondrial contributions.
However, without a direct transport assay, such as radiolabeled lactate uptake into purified mitochondria, or reconstitution of MCT1 into proteoliposomes, the data do not formally demonstrate that MCT1 itself transports lactate across the inner mitochondrial membrane.
Thus, while direct mitochondrial lactate import may be the most parsimonious interpretation, the current title, "Direct mitochondrial lactate import enables cardiac metabolic flexibility under stress," overstates what the data directly demonstrate. I suggest revising the title and limiting the phrase "direct mitochondrial lactate import" throughout the manuscript. More evidence-aligned alternatives include: "MCT1-dependent mitochondrial lactate oxidation enables cardiac metabolic flexibility under stress" or "Mitochondrial MCT1 enables cardiac metabolic flexibility under stress."
The authors can still present direct import as their preferred model in the Discussion. With these changes, the manuscript's claims would better match the data.

Open Access This Peer Review File is licensed under a Creative Commons Attribution 4.0 International License, which permits use, sharing, adaptation, distribution and reproduction in any medium or format, as long as you give appropriate credit to the original author(s) and the source, provide a link to the Creative Commons license, and indicate if changes were made.
In cases where reviewers are anonymous, credit should be given to 'Anonymous Referee' and the source.
The images or other third party material in this Peer Review File are included in the article's Creative Commons license, unless indicated otherwise in a credit line to the material. If material is not included in the article's Creative Commons license and your intended use is not permitted by statutory regulation or exceeds the permitted use, you will need to obtain permission directly from the copyright holder.
To view a copy of this license, visit https://creativecommons.org/licenses/by/4.0/

Thank you for taking the time to review our original manuscript and for considering our resubmission for publication in *Nature*. We greatly appreciate the referee's thoughtful feedback and the opportunity to address their concerns. We were encouraged by the referee's positive assessment and acknowledgement that our '*work is important*', is based on '*experiments … [that] are generally well conducted'* and presents '*a compelling argument*' for our hypothesis. Their insightful critiques have helped us to dramatically improve the manuscript, including the addition of several pieces of new data that convincingly address points of the referees and increases the physiological relevance of the work. Below are our point-by-point responses in red to their specific critiques.

Referee #1 (Remarks to the Author):

In this manuscript the authors provide evidence that lactate is directly metabolized by cardiac mitochondria and that mitochondrial lactate uptake is mediated by the mitochondrially localized monocarboxylate carrier (MCT1). The authors use extensive tracer labeling experiments in animals perfused in vivo and in isolated mitochondria to support the existence of functional lactate metabolism. A cardiomyocyte-specific MCT1 KO mouse is generated and in addition to impaired contribution of lactate derived carbons to TCA cycle intermediates, these animals reveal impaired resilience when subjected to diverse hemodynamic stressors including pressure overload hypertrophy and ischemia/reperfusion. The experiments presented are generally well conducted. However, there are some gaps in the data presented that limit the strength of some of the author's assertions.

Thank you for taking the time to review our work and providing such detailed and constructive feedback. We appreciate your thoughtful assessment of the manuscript and your recognition of the strengths of our experimental approach. Your comments have helped us to improve our study by addressing noted weaknesses through significant new experimental work and further refining our model of cardiac metabolism.

Specific Critiques

1. The authors begin by studying mice with inducible KO of the mitochondrial pyruvate carrier (MpcKO). The authors state that studies were performed in MpcKO mice at a time when these hearts exhibited normal contractile function. It is not transparently indicated how many weeks after Mpc KO were the studies performed. Moreover, it has been previously shown by several groups, including the authors, that Mpc KO mice spontaneously develop heart failure as a function of duration of loss of the pyruvate carrier. As such, it is essential that the authors also analyze Mpc KO animals at a time that they are failing, to determine if the failure of these hearts correlates with loss of an ability to metabolize lactate (or something else).

We appreciate that referee #1 asks how our study identifying an MCT1-dependent mechanism of glucose carbon oxidation can shed light on the phenotypes of *Mpc1^{iCKO}* mice and vice versa. We agree that our study can be expanded to provide additional insights on these animals and provide a mechanistic hypothesis for metabolic-induced heart failure more broadly. We specifically address critique 1 here, but our responses to specific critiques 2, 3, 4 and 5 all share a common framework, and we worked to address them collectively using a substantial set of new experiments.

To address the metabolism of MPC1 mice that had gone on to develop spontaneous heart failure after MPC1 loss, we performed several additional experiments in *Mpc1^{iCKO}* mice at a time point (>14 weeks post injection with tamoxifen) when these animals exhibited clinical signs of heart failure (LVEF < 30% and significant cardiac hypertrophy). To be able to quantitatively assess their cardiac metabolic function, we developed a new bolus-initiated rapid steady-state isotope tracing protocol administered by tail vein infusion in anesthetized animals who were too weak to survive jugular vein catheterization. Using both [U-^{13}C]lactate and [U-^{13}C]glucose tracers, *we show that failing Mpc1^{iCKO} hearts exhibit a profound reduction in carbohydrate oxidation* compared to earlier time points when contractile function is preserved (Fig 1f-j, Extended Data Fig 2b-f). This loss of oxidation is accompanied by a change in the abundance of mitochondrial MCT1 which was increased in *Mpc1^{iCKO}* mice before HF (Extended Data Fig. 2i) but now decreased compared in controls in failing *Mpc1^{iCKO}* mice (Fig 1j). While we don't have the data resolution to show direct causation, these *new data show a compelling correlation between loss of*

lactate oxidation and heart failure onset, an observation we repeat with detailed cardiometabolic analyses of *Mct1*^{iCKO} hearts upon Ang/PE treatment (Fig 4c, 4j). The new data has been incorporated into the revised manuscript and discussed in the Results and Discussion sections.

2. A fundamental question that is not addressed by this study is: Why do unstressed Mpc KO mice spontaneously fail, whereas MCT1 KO mice do not? Preserved lactate oxidation (if true) does not appear to be sufficient to prevent them from failing. Do Mpc KO mice lose MCT1 expression as they fail, or is there some other mechanism that prevents lactate utilization from "rescuing" Mpc KO hearts are they age? Does lactate oxidation also fall in Mpc Ko hearts as they fail? Thus, it will be important for certain key experiments (including substrate tracing) to be done in both models in parallel, in stressed and non-stressed hearts.

Our new studies conducted in spontaneously failing of *Mpc1*^{iCKO} hearts demonstrate a loss of glucose and lactate oxidation consistent with acquired loss of MCT1 function, phenocopying our dual MPC1/ MCT1 KO experiments done with inhibitors (Fig. 2a). We measured expression of MCT1 in mitochondria from *Mpc1*^{iCKO} hearts and observed a trend towards reduced expression that was not present at earlier timepoints pre-heart failure (Fig 1j and Extended Data figure 2j). *Our most simple model is that MCT1 and MPC can compensate for each other and support mitochondrial carbohydrate oxidation and thus cardiac function*. We saw clear loss of MPC1 expression coupled with a reciprocal increase in MCT1 expression in hearts subjected to TAC (Extended data Fig 9l-m). Upon hypertrophic stimuli, MPC expression and activity is suppressed, diverting more glucose towards anabolic pathways and MCT1 becomes essential for mitochondrial oxidation of carbohydrates. In this scenario, chronic diversion of glucose to anabolism leads to hypertrophic growth and HF which does not occur in MCT1 ko hearts that do not alter glucose uptake and oxidation. In models of chronically developing HF, MCT1 expression/ activity at some point becomes insufficient to support mitochondrial energetics precipitating a crisis. However, engineered loss is well tolerated but lack of MCT1 can be acutely lethal in contexts when the MPC is impaired or additional carbohydrate oxidation flux is required leading to mitochondrial dysfunction and enhanced ROS production. We have incorporated this model into our final figure (Fig. 4p) and discuss its strengths and limitations within a revised discussion.

3. Although the authors show extensive data that MCT1 KO animals fail in response to various hemodynamic stressors, this is also true of Mpc KO mice as well. Therefore, both models demonstrate that substrate switching between lactate and glucose is not sufficient to maintain cardiac function. The story would therefore benefit by substrate tracing in both sets of mutant animals under conditions of hemodynamic stress, to more comprehensively address this important issue.

We are happy to report that we were able to successfully implement cardiometabolic substrate tracing in *Mct1*^{iCKO} subjected to Ang/PE that show signs of acute HF (reduced LVEF) (Fig. 4j, Extended Data Fig. 10d). We did this using the same bolus infusion technique we implemented for aged *Mpc1*^{iCKO} mice (see critique 1). In these difficult experiments, lactate utilization by the heart was impaired selectively in the *Mct1*^{iCKO} mice, correlating with loss of function. These data show convincingly that upon Ang/PE treatment, lactate carbon oxidation is impaired only in *Mct1*^{iCKO} mice who are showing overt signs of HF and not in control animals. We complemented these tracing studies with additional mitochondrial characterization (TEM) in our revamped figure 4. These structural studies provide some additional mechanistic insight to explain the apparent inability of MCT1 deficient hearts to sustain bioenergetics. We find that there are subtle but significant mitochondrial and cristae morphological differences present at baseline in *Mct1*^{iCKO} hearts (Extended Data Fig. 10 f-j). However, upon Ang/PE treatment, these differences are much more profound and include large changes in cristae volume density (Fig. 4l). We further showed that these structural changes connect to function be performing measurement of mitochondrial ROS production from ACMs isolated from *Mct1*^{iCKO} and control hearts (Fig. 4n). When subjected to oxidation stress (I/R). small baseline changes in ROS production between genotypes become very large and may provide an additional mechanistic explanation for the severity of the phenotype we observed *in vivo*.

4. Moreover, it appears that angiotensin/PE treatment leads to reduced expression of MPC, thus is the LV dysfunction that develops following Ang/PE largely a function of MPC loss, which is further exacerbated in MCT1 KO mice? Could these animals be rescued by maintaining MPC expression? Furthermore, seeing that MPC levels

decline in Ang/PE controls that do not appear impact LV function (figure 4C) and impacts LV function to a smaller degree after TAC (Figure 4D), then would MpcKO mice have the same relatively more benign phenotype as seen in Ang/PE , TAC-treated or MI treated WT mice? Based on prior literature this might be unlikely. What happens to Mpc and Mct1 protein levels in hearts after TAC?

We agree with the referee's interpretation of our study results. In response to specific critique 2 above, we now report expression of the mitochondrial pyruvate carrier (MPC1 and MPC2) and the major cardiac monocarboxylate transporters (MCT1/2/4, Slc16a1/ Slc16a7/Slc16a3) in all our models of heart failure included in the study (MPC aged, Angiotensin/ Phenylephrine, trans-aortic constriction, and ischemia reperfusion). Overall, we see MPC loss across most models in failure and never an increase. Due to the technical challenges inherent in quantifying cardiac metabolism in animals with heart failure, we were only able to perform detailed tracing studies to measure *functional* glucose and lactate oxidation outcomes in 2 models. In both models (Ang/PE and MPC aged), active heart failure was associated with reduced glucose and lactate oxidation. Based on the report of Zhang et al. (pmid: 33106689), which reported accelerated HF in *Mpc1*^{iCKO} mice subjected to TAC, we anticipate that *Mpc1*^{iCKO} mice would not have a more benign phenotype than MCT1 when subjected to the stressors in our study.

5. In the ischemia model, control animals regain function after 6-7 weeks, but MCT1 KO mice do not. In this model, what happens to MPC expression in KO and control cohorts? Do MPC KO mice, that also maladapt in response to hemodynamic stress behave any differently?

Thank you for raising this important point. To address this question, we performed qRT-PCR analysis in the *Mct1*^{iCKO} hearts following 9 weeks of ischemia/reperfusion (I/R) injury (same hearts). These data show a significant reduction in Mpc1 with increased LDHA, and LDHB expression in *Mct1*^{iCKO} hearts compared to *Mct1*$^{fl/fl}$ controls. These findings suggest that impaired recovery in *Mct1*^{iCKO} mice after I/R injury is associated with a failing compensatory metabolic response where mitochondrial oxidation is impaired through reduced Mct1 and Mpc1 expression, but the heart is attempting to boost glycolytic flux through lactate-pyruvate cycling in the cytosol. We have included these results in the revised manuscript (Extended Data Fig. 9 and 11) and expanded the Discussion to address the potential mechanistic implications.

6. Although many experiments are performed I non-beating adult cardiomyocytes to indicate that these cells metabolize both lactate and glucose-derived pyruvate via distinct pathways, this approach does not address what happens to relative substrate utilization in intact beating heart preparations, which would be more physiologically relevant.

We thank the referee for suggesting this impactful experiment to further support our central hypothesis. Our new data from the Langendorff *ex vivo* perfusion system avoids the confounding effects of substrate interconversion inherent to *in vivo* studies and *provides physiologically relevant evidence supporting our conclusions*. Moreover, this was done in an intact, working heart under conditions that are as close to metabolically physiological as we could engineer *ex vivo*. In our new experiments conducted with a mix of complete physiological substrates (fatty acids/ glucose/ lactate/ pyruvate) supplemented with insulin, epinephrine and carnitine, ex vivo perfused *Mct1*^{iCKO} hearts were unable to metabolize and oxidize lactate but still maintained normal heart rates (Fig 2i-l). This data shows that the observed lactate oxidation *in vivo* is a product of both MCT1 import into mitochondria and lactate labeling of glucose substrates in the circulation, but these can be fully distinguished using the Langendorff system. Consistent with our central hypothesis of substrate redundancy between MPC and MCT1 mitochondrial pathways, glucose carbon incorporation into TCA cycle intermediates was significantly increased in *Mct1*^{iCKO} hearts, confirming that substrate switching occurs in response to impaired lactate utilization.

7. It is not clear how lactate mediated respirations remain normal in CPI-613 treated cells, in which PDH and a-KGDH is inhibited. Is this "normal" oxygen consumption coupled to ATP generation?

We appreciate the validity of the referee's questions about the interpretation of these oxygen consumption assays and acknowledge our results were not sufficiently well-described. The key to interpreting the CPI-613 assay rests on understanding that purified mitochondria respire poorly on lactate (and ketone bodies in data not shown).

These findings with ketone bodies are not unique to us (PMCID: PMC12171989, https://www.biorxiv.org/content/10.1101/2024.06.27.600966v2.full) . In short, the field simply does not have a clear explanation for the large discrepancy in substrate usage between tracing studies conducted in either hanging hearts or live animals and those conducted in purified mitochondrial. We inherently put more trust in studies performed in intact hearts, and we use the results from purified mitochondrial studies more sparingly to ask specific mechanistic questions. With respect to lactate, we propose that the absence of pyruvate, which is necessary to assess lactate respiration, plays a large role in repressing apparent respiration. This could be either due to pyruvate now failing to block lactate outflow via the MPC (and thereby failing to fully oxidize lactate) or because pyruvate is required for activation of transport processes and/ or oxidative fluxes. In support of this interpretation, our data shows that lactate respiration in intact cells is improved when the MPC is inhibited pharmacologically (Extended Data Fig. 6q). In summary, our CPI-613 data show respiration that is best explained by NADH generation from the LDH reaction where there is minimal NADH generation from the TCA cycle. Due to the limitations of these studies that you have kindly emphasized to us; we have moved these results to the extended data.

8. The authors show that mitochondrial MCT1 is increased in failing human hearts, but it is not clear what this actually means. If their thesis is that lactate utilization may be an adaptation that could potentially preserve cardiac function in failing hearts, then it is not apparently of benefit in this circumstance. In another part of the paper, the authors actually measured mitochondrial respirations in human heart derived mitochondria. The experiments were presumably designed to show that human mitochondria can respire on lactate. It is not clear why experiments were not done on mitochondria from failing hearts to determine if the increase in MCT1 correlated with increased lactate utilization. The authors also have access to cardiac tissue from LVAD samples of individuals whose LV function recovered. What happens to MCT1 content in this context? In the absence of these analyses no conclusions can be drawn from the observation that MCT1 protein is increased in failing human heart mitochondria.

As the referee notes, our group does collect specimens from HF patients including explanted failing hearts and apical cores from LVAD placements, and we were able to successfully leverage these resources in our initital submission and in our revised manuscript. We bank these samples and have used them for extensive transcriptomics, proteomics and metabolomics data analysis. This represents over 15 years of work. From these human data, we showed that MCT1 is localized to the mitochondria of human myocardium in a manner indistinguishable from mice (Fig. 2b, Extended Data Fig 5a-e, k). We further showed that human mitochondria are competent to oxidize lactate, and this depends on MCT1 (Fig. 4m). Finally, as the reviewer noted, we observed an increase in mitochondrial MCT1 localization in human HF patients. Together with our extensive animal data showing a critical role for MCT1 in supporting cardiac function upon stress, these data strongly support a similar role for MCT1 in human heart function. We acknowledge that we were unable to show functional data on lactate oxidation from failing human hearts, but these experiments require fresh tissue and that is very difficult to obtain within the compressed timeframe of a revision. The data we showed in the original submission came from fresh hearts and represent patients with heart failure of diverse etiologies as represented by the clinical cases that occurred during the research period prior to the submission. We concur with the reviewer that it would be compelling to show MCT1 data from paired LVAD and transplant samples from the same patient, but due to changes in transplant priority lists, and better management of LVAD patients, our volumes of explanted 'recovered' hearts from LVAD patients have significantly dropped. Within the timeframes of this revision, we could not collect fresh heart tissues from patients pre LVAD and post-transplant (often representing an 18 month to 2 year gap) to be able to compare MCT1 expression in the mitochondria. To attempt to overcome this, we returned to frozen tissue from *the same patients* from whom we freshly isolated mitochondria and assayed for MPC1 expression in the whole heart. We observed no difference, and the tissue quality was not sufficient to perform reliable fractionation studies. To truly test our hypotheses in human hearts, a sequence or proteomics-based assays to quantify MCT1 localization is needed, but despite our best efforts over 2+ years, we have been unable to develop such a method. We interpret our heart data showing that MCT1 increases within the mitochondria fraction as useful and indicating a role in pathology, but we acknowledge the ambiguity in how to interpret these data in our results and discussion.

9. Immunohistochemistry of MCT expression in cardiomyocytes is of low quality.

We comprehensively revisited our immunoblotting techniques for MCT1 expression and present almost entirely new data in our revision. Only a small fraction of total MCT1 lies on the mitochondrial membrane making immunoblotting and immunofluorescence challenging. To address this, we dramatically improved our blotting and fractionation techniques and present significantly improved immunoblots of MCT1 from whole lysates and purified mitochondrial fractions and they show consistent MCT1 localization to mitochondrial fractions. Furthermore, we enhanced our complementary approaches such as extensive use of proteinase K assays on purified mitochondria that clearly demonstrate localization of MCT1 to the inner mitochondrial membrane (Fig. 3a,b, Extended Data Fig. 5f-k). The proteinase K assays (performed on whole fresh mouse hearts, mouse primary cardiomyocytes, human cell lines and human heart tissue) give strong biochemical support to the localization of MCT1 in the inner mitochondrial membrane, something that was never possible with immunofluorescence techniques that rely on correlation. These results gave us the confidence that the supportive but inelegant immunofluorescence data should be removed in the revised submission.

10. Figure 3H shows cpi enrichment from labeled lactate. What is happening to the total NAD pool (labeled and unlabeled in these experiments)? Similarly for figure 3I to be fully interpreted NAD+ and the NADH/NAD+ ratio is necessary to be shown.

We revisited this data to present the total pool sizes as requested. In doing so, we discovered that our team had made an error in the original analysis of the mass spectrometry data. The new reanalysis in the revised manuscript was directly performed by Dr. Ducker, an expert in stable isotope tracing. Using newly collected multiple independent standards and dilution curves for isotopic enrichment, we now realized that the data collected could be interpreted in ways other than convincingly demonstrating ^{2}H tracing into NADH. We performed extensive troubleshooting and theoretical calculations to try to make this experiment work with our instrumentation, but we were unable to overcome biochemical (NADH half-life and labeling abundance) and technical limitations (instrumentation sensitivity and resolution). Out of an abundance of caution, we have decided to remove these data from the manuscript. Dr. Ducker has personally re-reviewed all other raw data and analyses of isotope tracing data presented in the manuscript and confirmed its validity. We apologize for this error.

11. Because MCT1 is expressed in the plasma membrane and the mitochondria the authors should acknowledge that this imposes some limitation on their interpretation of in vivo studies in the MCT1KO model, because of the inability to distinguish between effects that could be due to cytosolic versus mitochondrial lactate abundance and metabolism.

We also share this concern and present additional experimentation to address this. However, we want to highlight that the heart is unique among major adult tissues in that it also expresses a second very high affinity lactate transporter, MCT2 (PMID 22131303). Our data strongly suggest that MCT2 can account for normal plasma membrane lactate transport in MCT1 deficient cardiomyocytes. In our new fractionation and immunoblotting studies, we show high expression of MCT2 in whole heart but never present in mitochondria (Fig. 1k, 2c). Experimentally, we performed new kinetic [U-^{13}C]lactate uptake assays in adult cardiomyocytes isolated from *Mct1^{iCKO}* and *Mct1$^{fl/fl}$* animals (Fig. 2d-e). These experiments revealed no differences in the rate of M+3 lactate uptake between groups, suggesting that plasma membrane lactate transport is unaffected. We also note that in all *in vivo* and hanging heart samples, total labeled lactate enrichment was not different between *Mct1^{iCKO}* and *Mct1$^{fl/fl}$* groups again suggesting that MCT2 was able to compensate for lactate plasma membrane transport. That loss of MCT1 would have a unique effect on mitochondria transport is further supported by our new quantification of *highly elevated intracellular levels of lactate in Mct1^{iCKO} cells* suggesting an inability to oxidize lactate.

12. It appears that the major adaptation in MPC1 KO animals is increased lipid oxidation. Why is fractional lactate use not increase in MPC KO hearts despite a clearly and substantially increased expression of MCT1?

Our data presentation in the first submission suggested a trend towards increased FAO in *Mpc1^{iCKO}* animals, but this was not significant. To avoid future confusion, we have presented this data with less processing in our revised

manuscript ((Extended Data Fig 1k,l). There is no statistical difference in either palmitate or oleate oxidation contributions to TCA labeling between non-failing *Mpc1*^{iCKO} and *Mpc1*$^{fl/fl}$ animals.

The use of lactate by MPC KO hearts paradoxically decreases. This can be explained by understanding that the measured nutrient contribution to the TCA cycle reflects nutrient uptake from outside the heart, not the mechanism by which it enters the mitochondria. If one further makes the reasonable assumption that cytosolic LDH carries directional reaction flux (and is not at equilibrium), then it is expected that changing pyruvate entry into the mitochondria could impact lactate fuel usage. When the MPC is lost, there is more pressure on the forward cytosolic LDH reaction (lacate-producing) and consequently a greater fraction of cellular lactate comes from glycolysis and not lactate uptake. This glucose-derived lactate is now a substrate for MCT1 for oxidation within the mitochondria. This flexibility demonstrates the limitations of carbon tracers to establish mitochondrial transport, and highlights the mechanistic impact of our innovative ^{2}H tracer approach coupled with Langendorff perfusions to establish direct lactate uptake into mitochondria dependent upon MCT1 (Fig. 3d,e)

Minor Issues

1. Please show the actual concentrations of metabolites in HPLM
In streamlining our manuscript, we have clarified that our use of HPLM relies on the commercial formulation (GIBCO A4899101) supplemented with cardiomyocyte specific factors (See methods).

2. Extended Figure 5 panels b-e: Figure would be easier to understand if a line was drawn about panels b, c, d,e to indicate that these are being supplied with labeled lactate. Please clarify the differences between data shown in l,m and data shown in n,o.

We have improved the labeling of our figures (now Extended Data Fig. 6g-k) to more easily show which substrate is being used to label with either lactate or glucose.

<u>Referee #2 (Remarks to the Author):</u>

The manuscript presented by Cluntun et al presents a novel finding that lactate can directly enter cardiomyocyte mitochondria in a non-MPC-dependent manner. They suggest that lactate entry into mitochondria occurs via MCT1 as mitochondrial lactate entry is significantly reduced when MCT1 is absent or inhibited. Using both in-vivo and ex-vivo isotopic tracing methods, they present compelling evidence to show that lactate can be directly oxidized within mitochondria thus generating an intra-mitochondrial pool of lactate-derived NADH. They go on to demonstrate that mice lacking cardiomyocyte-specific MCT1 are more susceptible to heart failure following neurohormonal and ischemic insults, suggesting that MCT1 plays a biologically meaningful role in cardiac adaptation to physiological stressors.

The question of whether lactate can enter and undergo oxidation within mitochondria was proposed many years ago and has been highly controversial. The authors present a compelling argument that this is in fact true, at least in cardiac mitochondria. The meat of the matter is figure 3, demonstrating lactate oxidation in intact mitochondria. The work is important; however, several additional experiments are needed to support physiological relevance and explain certain paradoxical findings.

Thank you for taking the time to review our work and provide detailed and constructive feedback. We greatly appreciate your thoughtful evaluation of our manuscript and your recognition of its strengths. Your comments have been instrumental in helping us refine the study, and we have carefully addressed each point to strengthen the clarity and rigor of our conclusions.

1. Overall, the impact of the paper could be significantly strengthened if the authors were to test the role of mitochondrial MCT1 without impacting plasma membrane MCT1 activity. In the absence of such an experiment, it is impossible to discern whether cardiac MCT1 KO predisposes to heart failure due to loss of plasma membrane or mitochondrial function. For instance, if MCT1 has a mitochondrial localization sequence, such an experiment

could involve deletion of the localization sequence and testing lactate oxidation/respiration in isolated cardiomyocytes and, if feasible, response physiologic response to cardiac injury. Alternatively, one could delete MCT1 and then reintroduce PM-targeted vs mito-targeted MCT1.

The referee raises an important point regarding the plasma membrane and mitochondrial roles of MCT1. We concur that an experiment that could distinguish these forms from one another would strengthen our claims about mitochondrial specific functions of MCT1. Despite considerable effort, we have not identified a natural targeting mechanism or localization sequence that specifically directs MCT1 to mitochondria versus the plasma membrane. Despite this limitation, *we believe that the totality of our data, including new data for our resubmission highlighted below can only support one conclusion: that MCT1 has a non-redundant and essential role in mediating lactate oxidation in the mitochondria*. Our data strongly suggest that plasma membrane MCT1 activity is dispensable for the phenotypes observed. This is supported by the presence of additional MCT isoforms (MCT2 and MCT4) in the heart, which likely mediate lactate transport across the plasma membrane. In *new immunoblots*, we highlight the presence of MCT2 and 4 in cytosolic and whole heart fractions and their absence from the mitochondria (Fig 1j, 2c, 4g). We also performed a kinetic lactate uptake assay in adult cardiomyocytes that lacked MCT1 and observed no defect in lactate uptake (Fig 2d,e). Finally, we note that in extractions from [U-^{13}C]lactate *Mct1*^{iCKO} infused hearts, we never observe a deficit in lactate labeling (Fig. 2h, 2k). Given our experiment showing that glucose carbon oxidation in *Mpc1*^{iCKO} animals depends upon MCT1 (Fig. 2a), and the data showing mitochondrial metabolic dysfunction, we conclude that the relevant observed phenotypes are due to loss of MCT1 function in the mitochondrial membrane.

2. Figure 1 redemonstrates the fate of glucose in the MPC1 cardiac KO as originally reported in Y. Zhang, ED Abel et al Nature Metabolism 2020. The original study is not cited in this section of the manuscript though it should be acknowledged. Here the authors recapitulate most of the original findings, including two perplexing observations that should be explored experimentally.

a. First, there is a striking increase in 13C-glucose derived enrichment of myocardial lactate and pyruvate that is not explained. Could this be due to suppressed cardiac glycogen breakdown in the MPC1 CKO (Figure 1c)?

b. Second, there is significantly increased 13C-glucose contribution to succinate and malate in the MPC1 cKO that is striking though not discussed (Figure 1c). How do the authors explain this observation? This finding is especially intriguing given that the authors find less, not more, lactate contribution to TCA cycle in the MPC1 KO condition (Extended Data 1m), implying that the increased 13C label in the TCA cycle in MPC KO is not coming from lactate.

c. Finally, the authors find here a trend towards less 3-HB contribution to succinate and malate in MPC CKO, which is quite a contrast from the 2-fold increase in ketone-derived contribution to TCA in Zhang et al. This may be due to different ketone concentrations found in plasma (here) vs. perfusion (Zhang 2020). Given that ketogenic diet rescues HF in MPC1 CKO, the contrasting finding between studies merits discussion.

It was an oversight on our part to not cite the findings of Zhang et al. in our original results sections which we now have corrected. We additionally note that the group of Kyle McCommis more recently reported a similar finding on BioRxiv (PMID: 38895296) and we also include that.

a) We observe a relative shift in source of cardiac lactate and pyruvate in *Mpc1*^{iCKO} away from serum lactate and towards glucose. We interpret that as an increase in pyruvate oxidation and lactate production driven in part of loss of the MPC (a well-documented metabolic response; pmid: 25458841) and increase in glucose uptake (see our detailed explanation in response to critique 12 from referee #1). This interpretation is supported by our new report of total glycogen levels in *Mpc1*^{iCKO} and control hearts in which we observed no difference (Extended Data Fig. 2g).
b) The TCA cycle labeling pattern closely follows the pyruvate and lactate labeling, and we see this in nearly all our infusion experiments (Fig 1c) . So, we interpret increased succinate from glucose labeling because of the relative shift of pyruvate and lactate coming from glucose and not serum lactate in the *Mpc1*^{iCKO} hearts. When we summarize the contributions of serum lactate and glucose to TCA labeling based on the individual TCA labeling fractions (Extended Data Fig 1f-i) we do in fact observe a decrease in lactate

contribution to TCA oxidation (Extended Data Fig. 1j). But importantly this is reporting on the carbon source of the cardiomyocyte itself, not the form in which that carbon entered the mitochondria for oxidation. Our subsequent data shows that lactate transport by MCT1 into mitochondria is the most likely source of MPC alternative transport.

c) While there does appear to be a modest trend towards reduced ketone oxidation in our *Mpc1*^{iCKO} hearts, it was not statistically significant. We have changed our presentation of the data to more readily show statistically significant comparisons between fuel usage of control and *Mpc1*^{iCKO} hearts (Extended Data Fig 1k-m). We share the referee's opinion that the best explanation for the discrepancy lies in the very different concentrations of ketones used in a perfusion experiment versus a *in vivo* tracing experiment in a briefly fasted mouse where natural ketone bodies are very low.

3. Figure 2 has several major weaknesses.

a. First, the colocalization images (2a-d) are of insufficient resolution to support the claim that MCT1 localizes to the IMM as opposed to the OMM. The distance between the inner and outer mitochondrial membranes is ~20 nm, whereas the scale bar is 5-10 microns. Additionally, there is no quantification of images. And it appears graphs are taken selectively from one area per panel.

We thank the referee for asking us to revisit and improve our immunofluorescence images. After extensive troubleshooting and testing of multiple different primary antibodies against MCT1, including multiple lots from the same manufacturer, we regrettably conclude that commercially available MCT1 antibodies are not of sufficient quality to achieve the level of specificity and resolution required for confident interpretation of subcellular MCT1 localization. We tried multiple confocal imaging platforms and repeatedly, poor antibody binding and signal was limiting. To maintain the rigor and clarity of the manuscript, we have removed all immunohistochemistry data from the revised version. As we describe in reply to referee 1 (point 9), we have been able to improve immunoblotting with anti-MCT1 antibodies and present new blots throughout the manuscript. We further rely on improved and replicated proteinase K assays (see b).

b. Second, the proteinase K protection assay is poorly described and lacks quantification or being done more than once. The authors should describe the swelling condition and what is meant to be concluded from the swelling intervention.

As we outlined in response to referee 1, we have completely revamped our Proteinase K protection assays and present several new blots from multiple tissue sources. To show reproducibility, we now present data conducted by independent scientists working in physically different locations. Together we show proteinase K assays from 4 independent freshly isolated mouse hearts (Fig. 3a and Extended Data Fig. 5f-h), 4 new human hearts using previously frozen samples (Fig 3b and Extended Data Fig 5k, purified mouse adult cardiomyocytes (Extended Data Fig 5f and human cells -Extended Data Fig. 5j). We have replaced the 'swelling' notation with the more standard hypotonic buffer and added additional experimental detail in the results and methods to improve clarity for the reader.

c. Finally, the figure title ("Lactate oxidation requires MCT1") is not justified given that there is substantial residual lactate-derived 13C contribution to succinate and malate in MCT1 iCKO hearts. In fact, it appears there is equivalent if not more 13C-lactate derived enrichment of succinate/malate in Mct1 iCKO than there is 13C-glucose-derived enrichment of succinate/malate in WT animals. As a minor point, the y-axis of Figure 2g should be corrected to "13C enrichment" or "13C labeling contribution" as it appears the data reflect enrichment and not total metabolite abundance.

Based on the suggestion in question 4, we have significantly strengthened the evidence behind our claims in Figure 2. Most importantly, we now present data from Langendorff perfusions that demonstrate lactate oxidation is entirely dependent upon MCT1 (Fig. 2i-l). This finding overcomes some of the limitations of *in vivo* tracing data highlighted by the referee. As noted, glucose and lactate interconversion can blunt the effects we observe. Again, we are limited by this conversion because both glucose and lactate are labeled in the serum generating labeled pyruvate and lactate in the cytosol. This prevents us from definitively assigning intracellular lactate and pyruvate

to a specific nutrient source, but this limitation motivated our Langendorff experiment where we can perfuse 100% labeled lactate and 0% labeled glucose into the same heart. We have reworded the title to "MCT1 is required for the oxidation of lactate" which reflects the results from our cleanest experiment, the hanging heart perfusion.

4. Figure 3 demonstrates technical feasibility for (1) lactate to enter cardiac mitochondria and (2) for lactate oxidation to directly produce NADH outside of the TCA cycle.

a. While striking, the conclusion of the figure would be better supported by additional experiments to support physiological relevance. The data demonstrates that lactate CAN enter mitochondria directly and be oxidized, but the important physiological question is DOES lactate do this under normal circumstances, or is this an artifact of the in vitro conditions? The authors could consider repeating the 2-2H lactate infusion with NAD2H quantification in an isolated (i.e. Langendorff) heart model, and quantify ACM and mitochondrial NAD2H, which would support the physiological relevance of mitochondrial lactate observation.

Thanks to the referee's insightful suggestions, we are pleased to report that have further pushed this system by performing ^{2}H lactate tracing coupled with MITO-Tag purification to rapidly enrich mitochondrial metabolites (Fig 3d,e and Extended Data Fig. 6a,b). In doing so, we were able to overcome tracer interconversion limitations inherent in living systems and the slow speed of tissue isolation to *rapidly purify organelles from beating hearts and quantify their metabolism*. In this new experiment we perfused hearts with ^{2}H lactate and rapidly extract mitochondria to quantify metabolite enrichment. *In these mitochondria, we observed ^{2}H lactate in WT but NOT $Mct1^{iCKO}$ hearts.* We performed optimization studies to investigate NADH labeling, but isolation of cardiac mitochondria was fundamentally too slow to recover $NAD^{2}H$ before loss of the hydride to other reactions. When combined with our new Langendorff studies using ^{13}C lactate, *we can directly observe with confidence mitochondrial import and oxidation that of lactate in perfused hearts* that are both dependent upon MCT1.

b. In Figure 3k, there is a surprising finding that merits discussion: it is not clear here why respiration is not suppressed by CPI-613. The pyruvate produced by LDHB should still be oxidized within the TCA cycle and thus respiration should be suppressed with CPI-613. Why is this not the case?

We addressed this same concern in response to critique 7 from referee 1 with the following: We understand the referee's questions about the interpretation of oxygen consumption assays and acknowledge our results were not sufficiently well-described. Our interpretation of the CPI-613 assay is based on understanding that purified mitochondria respire poorly on lactate (and ketone bodies in data not shown). These findings with ketone bodies are not unique to us (PMCID: PMC12171989, https://www.biorxiv.org/content/10.1101/2024.06.27.600966v2.full) . Simply put we do not have clear explanation for the large discrepancy in substrate usage between tracing studies conducted in both hanging hearts and live animals and those conducted in purified mitochondrial studies. We trust studies in intact hearts more and use the results from purified mitochondrial studies more sparingly to ask specific mechanistic questions. With respect to lactate, we believe that the absence of pyruvate (necessary to assess lactate respiration) plays a large role in repressing apparent respiration either through failing to block lactate outflow via the MPC (and thereby failing to fully oxidize lactate) or is required for activation of transport processes and/ or oxidative fluxes. In support of this interpretation, our data shows that lactate respiration in intact cells is improved when the MPC is inhibited pharmacologically (Extended Data Fig. 6q). In summary, our CPI-613 data show respiration that is best explained by NADH generation from LDH but minimal NADH generation from the TCA cycle. Due to the limitations of these studies, we have moved these results to the extended data.

5. Figure 4 shows that loss of cardiac MCT1 increases susceptibility to HF following AngII/PE treatment or ischemia/reperfusion injury.
a. While the phenotype is striking, it is not clear if the phenotype is due to lack of MCT1 at the plasma membrane, mitochondrial IMM, or both (see comment above). Additionally, given that MCT1 also contributes to mitochondrial ketone uptake, it is conceivable that MCT1 loss increases HF susceptibility due to defective ketone oxidation in addition to or instead of defective MCT1-mediated lactate uptake. 13C-hydroxybutyrate infusion (either isolated heart or in-vivo) would help to clarify this last question.

Using additional stable isotope tracing experiments, we can now bolster our claim that loss of MCT1 impacts lactate oxidation upon Ang/PE treatment in our revised manuscript (Fig. 4j). By optimizing a less invasive tail vein infusion protocol and using bolus infusion conditions to reach serum steady state faster, we placed less stress on the mouse enabling quantitative measurements of in vivo cardiac metabolism in mice with heart failure. These data show that lactate oxidation is significantly blunted in vivo and this is unique to *Mct1^{iCKO}* mice (Extended Data Fig. 10d). These data show convincingly that upon Ang/PE treatment, lactate carbon oxidation is impaired only in *Mct1^{iCKO}* mice who are showing overt signs of HF and not in control animals. We complemented these tracing studies with additional mitochondrial characterization (TEM) in our revamped figure 4. These structural studies provide some additional mechanistic insight to explain the apparent inability of MCT1 deficient hearts to sustain bioenergetics. We find that there are subtle but significant mitochondrial and cristae morphological differences present at baseline in *Mct1^{iCKO}* hearts (Extended Data Fig. 10 f-j). However, upon Ang/PE treatment, these differences are much more profound and include large changes in cristae volume density (Fig. 4l). We further showed that these structural changes connect to function be performing measurement of mitochondrial ROS production from ACMs isolated from *Mct1^{iCKO}* and control hearts (Fig. 4n). When subjected to oxidation stress (I/R). small baseline changes in ROS production between genotypes become very large and may provide an additional mechanistic explanation for the severity of the phenotype we observed *in vivo*.

We have also considered whether ketones could play a role in the phenotypes we observe. We performed in vivo ketone oxidation studies using ^{13}C 3-hydroxubutyrate and include them in this rebuttal. While we observe a modest trend for reduced ketone oxidation in *Mct1^{iCKO}* animals, it is not significant, and the overall levels of ketones are very low in chow-fed mice (note the split y-axis) and the differences are much less than we observed with lactate. As the core focus of this paper is on reciprocal lactate and pyruvate transport, we elected not to include these data in the revised manuscript. If the referee feels strongly about the publication of these results, we are happy to reconsider their inclusion.

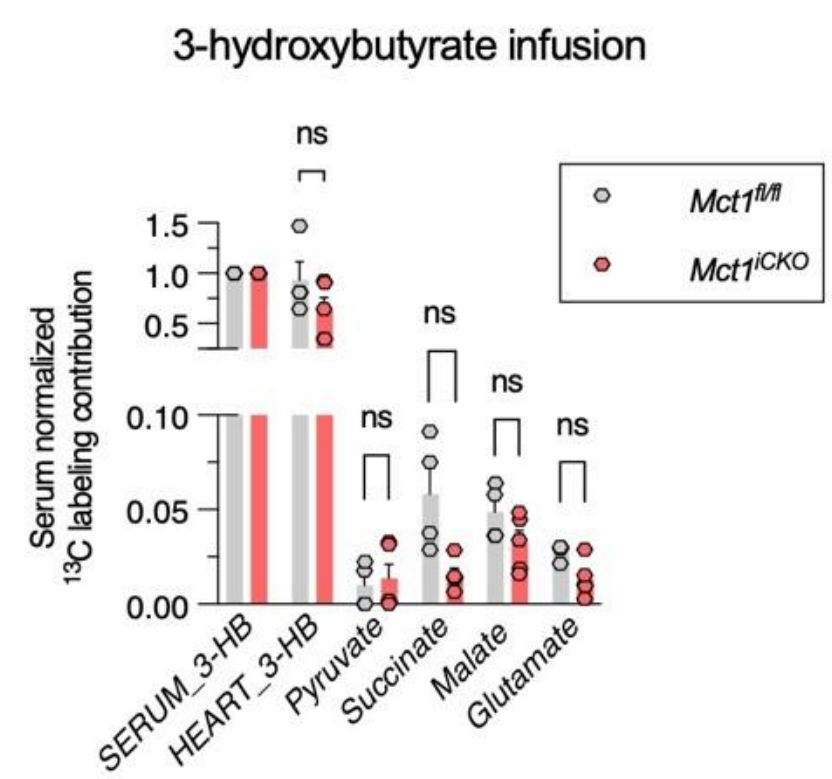

b. Finally, there is an interesting contrast between MCT1 CKO and MCT1 skeletal muscle KO as reported in L Zhang, Y Chen et al Science Advances 2024. Skeletal muscle MCT1 KO leads to increased PGC1a level, increased mitochondrial density, and increased running distance. Do similar changes (i.e. increased PGC1a and increased mitochondrial density) happen in the heart in cardiac MCT1 KO? If so, it is conceivable that increased mitochondrial density is detrimental in the setting of hypoxic stress such as ischemia/reperfusion injury.

We thank the referee for highlighting the contrast between our results and those of *L. Zhang et al*. reported last year in *Sci Adv*. They provide detailed and extensive tracing data in support of a model whereby MCT1 is the dominant lactate exporter in muscle cells and loss of lactate export increase pyruvate and glucose oxidation in addition to preventing lactate oxidation. In our study in heart, these apparent glucose oxidation effects are severely blunted (see Fig. 2g- the trend is in the direction reported for muscle but not significant in heart). When repeated in isolated perfused hearts, the increase in glucose oxidation was significant (Fig. 2l). We hypothesize that the different effects between our studies are due to the high expression of MCT2 in heart providing an alternative plasma membrane lactate transporter. As mentioned above, we have conducted qRT-PCR analysis on *Mct1^{iCKO}* hearts following 9 weeks of I/R injury, using the same cohort of hearts. The results demonstrate a significant downregulation of *Mpc1*, accompanied by increased expression of *LDHA* and *LDHB* in *Mct1^{iCKO}* hearts compared to *Mct1$^{fl/fl}$* controls. Notably, Slc16a3 (MCT4) and Pgc1α levels remained unchanged. We further analyzed the mitochondria of *Mct1^{iCKO}* hearts upon Ang/PE treatment and observed a strong reduction in mitochondria cristae volume density (Fig. 4k-l) that is further reduced upon Ang/PE treatment and correlates with reduced oxidative capabilities and impaired cardiac function.

<u>*Referee #3 (Remarks to the Author):*</u>

Cluntun et al. propose that lactate is directly imported into myocardial mitochondria and subsequently oxidized to generate NADH and pyruvate, thus supporting the TCA cycle/mitochondrial functions. Using isotope tracing and

genetic mouse models, this study starts to address ongoing debate in the field regarding the mitochondrial lactate oxidation. In agreement with prior findings from George Brooks et al., the authors also confirm that monocarboxylate transporter 1 (MCT1) is localized in the mitochondria and facilitates the import of lactate. Furthermore, they show that MCT1 loss leads to rapid progression to heart failure, along with compromised myocardial structure and function after injury. While this study is intriguing, there are some concerns as below.

Thank you for your time and thoughtful review of our manuscript. We appreciate your detailed and constructive feedback, as well as your recognition of the strengths of our work. Your insights have been invaluable in helping us improve the clarity and rigor of the study, and we have carefully addressed each of your comments in the revised version.

1) The study aligns with previous reports suggesting MCT1 is located in the mitochondrial membrane. However, one major concern is the purity of the mitochondrial preparations (e.g. Fig. 2G). Since MCT1 is expected to be predominantly in the plasma membrane, it would be useful to show both plasma membrane and cytosolic markers in the mitochondrial fraction. Furthermore, clarifying the proportion of MCT1 present in mitochondria versus the plasma membrane would provide some further insight for data interpretation. It would also be useful to report whether other MCT family members, such as MCT4, are present in the mitochondrial fractions. Mitochondrial purity could also be confirmed through proteomics analysis. Additionally, the microscopy images in Fig. 2A-D do not provide clear evidence of mitochondrial localization of MCT1; higher-resolution/magnification images would better support these findings.

We appreciate the referee's concern regarding mitochondrial fraction purity and the localization of MCT1. Similar points were raised by the other referees, and we have addressed these by performing extensive additional fractionation experiments and proteinase K digestion assays that *significantly improve upon our original results*. Specific fractionation assays that address these concerns are now included in new figures 1j, 4g, Extended Data Fig 2i, 4a, and 5a. These new experiments include a comprehensive panel of markers for all relevant compartments, as well as MCT isoforms (MCT1, MCT2, MCT4) and LDHA/B. We further validated the purity of our mitochondrial preps containing MCT1 by using untargeted proteomics. We performed MCT1 immunoprecipitation on purified mitochondria followed by LC–MS/MS proteomics and confirmed MCT1 peptide enrichment along with mitochondrial markers. Critically, we observed only minimal plasma membrane contamination (<2% of enriched proteins were plasma membrane–associated) whereas 43% were mitochondrial (Extended Data Fig. 5b–e). Finally, we would like to highlight our new proteinase K assays (*performed on whole fresh mouse hearts, mouse primary cardiomyocytes, human cell lines and human heart tissue*) that give strong biochemical support to the localization of MCT1 in the inner mitochondrial membrane, something that was never possible with immunofluorescence techniques that rely on correlation. *Collectively our new data relying on robust biochemical fractionation and digestion data strongly supports MCT1 mitochondrial localization*. Confident that these new biochemical and immunological assays show inner mitochondrial localization of MCT1, we have elected to not include our supportive but relatively weaker immunofluorescence data.

2) The evidence for "Direct lactate import assays" is not convincing. While Fig. 3i and Extended Data Fig. 5f-j suggest that lactate oxidation is impaired in purified mitochondria, these results could potentially be explained by cytosolic contamination of LDH in the mitochondrial preparations, which can generate NADH and pyruvate in the cytosol, wherein pyruvate could be imported by MPC. To strengthen these findings, it would be valuable to repeat Fig. 3i assay in a double knockout model of MPC/MCT1.

We share the referee's concerns that LDH contamination in our mitochondrial preparations could confound our tracing results. *To directly address this, we performed LDH activity assays on purified mitochondria* from fresh mouse hearts (Fig. 3f,g). In these preparations we observe only extremely minor LDH activity absent membrane permeabilization with alamethicin indicating that the overwhelming majority of mitochondrial LDH activity is membrane protected. To further confirm this, we additionally performed proteinase K treatments on mitochondria before alamethicin permeabilization (Fig. 3f). Proteinase K treatment decreased LDH activity in myocardial mitochondrial preps by only 10% indicating that the vast majority of LDH activity resides within the mitochondrial membrane (Fig. 3g and Extended Data Fig. 6e). While we concur that dual genetic knockout of MPC1 and MCT1

would also lead us to the same conclusion, we respectfully suggest that the LDH activity assays presented are a more direct readout of the contamination issue the referee raised.

3) At a minimum, it would be important to conduct mitochondrial uptake assays for lactate and pyruvate in a time-course fashion in both MCT1 and MPC1 knockout cardiomyocytes.

We agree that direct time-course mitochondrial-uptake assays would provide complementary kinetic information. We attempted to perform these measurements using both radiolabeled and ^{13}C-labeled lactate and pyruvate substrates in isolated cardiomyocytes and mitochondrial preparations from $Mct1^{iCKO}$ and $Mpc1^{iCKO}$ hearts. However, despite repeated optimization, the high fragility of freshly isolated adult cardiomyocytes and the rapid equilibration of monocarboxylates across compartments prevented us from obtaining reproducible quantitative uptake curves sufficient for publication. Absent this data we would like to highlight the additional new data in our revision that supports our conclusion of mitochondrial lactate uptake in cardiomyocytes:

a) LDH activity assays described above that show mitochondrial membrane protected LDH activity.
b) Steady-state [U-^{13}C]lactate and [U-^{13}C]glucose tracing in intact beating hearts (Fig. 2i–l), which provides an integrated readout of mitochondrial substrate utilization.
c) Proteinase K protection and fractionation assays confirming MCT1's mitochondrial localization (Fig. 3a, b; Extended Data Fig. 5f-k).

Together, these complementary approaches demonstrate that lactate oxidation is MCT1-dependent and that pyruvate import is MPC-dependent.

4) Using an LDH inhibitor in purified mitochondria, the authors propose that LDH activity may be present within the mitochondria and required for lactate oxidation. However, the purity of the mitochondrial preparation remains a concern, as LDH is highly abundant and could contaminate mitochondrial fractions. Clarifying the mitochondrial origin of LDH is essential, as well as identifying the specific LDH isoform(s) involved. To address the conflicting data in the field (PMID: 31610469; PMID: 30939431), both functional and genetic validation of mitochondrial LDH localization and activity are warranted.

We share the referees concerns over the ongoing conflicting reports in the literature about the localization of LDH activity. However, with the additional experimentation presented in this revision, including the perfused hanging heart (Langendorff) tracing data (Fig. 2i-l and Fig. 3d,e), *the totality of our data support a hypothesis of mitochondrial lactate oxidation*. To address concerns from the prior literature that lactate dehydrogenase is a potentially contaminating artifact of mitochondrial preparations we performed extensive new mitochondrial preparations and present data reporting on the purity of these mitochondria throughout the manuscript. Perhaps more convincingly, we now add lactate dehydrogenase assays performed on isolated mitochondria (Fig 3f,g). In these assays, we show that mitochondrial cardiac lactate dehydrogenase activity is dependent upon permeabilization in heart, independent of proteinase K treatment before permeabilization, and this is not equal in mitochondrial preparations among different tissues. We also present data from fractionation and digestion assays that a small but clearly detectable fraction of total LDHB is present in mitochondria. This aligns with MitoCARTA and prior proteomics-based approaches. We now acknowledge that our experimental data on LDHB and its activity cannot be solely assigned to the matrix and that LDHB activity could also be present in the inner membrane space, but in conclusion are data are strongly consistent with functional matrix mitochondrial LDH activity in heart.

5) Fig. 2j. It is surprising that the loss of MCT1, a well-known plasma membrane lactate importer, does not alter cellular lactate levels compared to the control. Could other MCT transporters be compensating for lactate import? A time-course assay assessing whole-cell lactate uptake in cardiomyocytes/infusions (Fig. 2j) would be important here to help determine if any differences in lactate uptake appear at earlier time points.

This experiment was suggested by referees 1 and 2 and we have performed the appropriate cellular uptake assays to address this concern. We responded to referee's 1 critique thusly, "However, we want to highlight that the heart is unique among major adult tissues in that it also expresses a second very high affinity lactate transporter, MCT2 (PMID 22131303). We believe that MCT2 can account for normal plasma membrane lactate transport in

MCT1 deficient cardiomyocytes. In our new fractionation and immunoblotting studies, we show high expression of MCT2 in whole heart but never present in mitochondria (Fig. 1k, 2c). Experimentally, we performed new kinetic [U-^{13}C]lactate uptake assays in adult cardiomyocytes isolated from *Mct1^{iCKO}* and *Mct1$^{fl/fl}$* animals (Fig 2d-e). These experiments revealed no differences in the rate of M+3 lactate uptake between groups, suggesting that plasma membrane lactate transport is unaffected. We also note that in all in vivo and hanging heart samples, total labeled lactate enrichment was not different between *Mct1^{iCKO}* and *Mct1$^{fl/fl}$* groups again suggesting that MCT2 was able to compensate for lactate plasma membrane transport. That loss of MCT1 would have a unique effect on mitochondria transport is further supported by our new quantification of *highly elevated intracellular levels of lactate in Mct1^{iCKO} cells* suggesting an inability to oxidize lactate."

6) The hepatic half of Cori-cycle consumes lactate for gluconeogenesis. Is the mitochondrial localization of MCT1 specific to cardiac tissues, or is it also present in hepatic tissue? Similarly, is mitochondrial lactate oxidation unique to the heart, or does it happen in other tissues or cell types?

This is a very interesting question but experimentally far beyond the scope of the present manuscript to systematically address. As a test of the generalizability of our observation, we performed LDH assays on mitochondria from heart/ lactate and kidney under the same conditions (Fig 3g). We find differences in the apparent localization of LDH activity between tissues with all LDH activity confined to mitochondria from heart tissue, but substantial LDH activity associated with the exterior of mitochondria from kidney. If LDH activity is coupled with MCT1 in the mitochondria, this suggests that MCT1 localization in mitochondria might be quite different between tissues as well. Data from stable infusions studies in mammals shows divergent abilities of tissues to oxidize circulating lactate suggesting potentially diverse mechanisms at play (Bartman et al, Nature 2023).

7) Extended Fig. 1c shows that lactate infusion leads to the production of M+3 glucose, indicating that gluconeogenesis is taking place. While the authors propose that cardiac lactate oxidation depends on MCT1, it remains unclear which of the two pathways, MPC or MCT, is required for directing pyruvate toward oxaloacetate and, subsequently, gluconeogenesis.

The observed gluconeogenesis is occurring systemically upon lactate infusion- a well-documented phenomenon (Hui et al., Nature 2017, Bartman et al., Nature 2023). Because of this largely liver-mediated phenomenon, using carbon tracers in vivo, we cannot formally assign mitochondrial carbon entry to the distinct MCT and MPC pathways because of substrate interconversion in the circulation. For all reported infusion experiments, we show circulating lactate and glucose isotope labeling fractions in the associated extended data figures to highlight this point. We instead rely on genetic knockouts but they are also limited by the systemic interconversion issue. We have worked to overcome this inherent limitation by performing ^{13}C infusions in beating ex vivo hearts using a non-recirculating buffer (Langendorff system). Because myocardium does not contain gluconeogenic enzymes, cardiomyocytes only see labeled lactate or glucose respectively without interconversion (Fig 1i-l). In these new experiments conducted with a mix of complete physiological substrates (fatty acids/ glucose/ lactate/ pyruvate) supplemented with insulin, epinephrine and carnitine, *ex vivo perfused Mct1^{iCKO} hearts were unable to metabolize and oxidize lactate but still maintained normal heart rates (Fig 2i-l)*. This data shows that the observed lactate oxidation *in vivo* is a product of both MCT1 import into mitochondria and lactate labeling of glucose substrates in the circulation, but these can be fully distinguished using the Langendorff system. Consistent with our central hypothesis of substrate redundancy between MPC and MCT1 mitochondrial pathways, glucose carbon incorporation into TCA cycle intermediates was significantly increased in *Mct1^{iCKO}* hearts, confirming that substrate switching occurs in response to impaired lactate utilization. We also clarify in the text that our isotope labeling data show only M+1 and M+2 peaks, indicating that pyruvate is only entering the TCA cycle upon conversion to acetyl-CoA.

8) In Fig. 2e, what do the authors mean by "swelling"? This is not explained in the methods or figure legends. Additionally, the quality of the blots is suboptimal, and quantification would be helpful.

This was a concern by another referee as well. We apologize for the confusion. We have added several new proteinase K protection assays to our manuscript under various conditions such as cells, mouse hearts, and human myocardium. We have also described the swelling condition in more detail throughout the manuscript and now use the more standard ‘hypotonic’ buffer nomenclature to improve clarity for the reader.

9) Confirming the expression levels of other MCT, MPC, and LDH isoforms in the presented knockouts, both at whole-cell and mitochondrial fractions, would provide useful insight and controls. Is there potential compensation by MPC2 in the absence of MPC1?

We have repeated all fractionation experiments to address this valid concern, please see our responses above. We note that MPC1 and MPC2 form obligatory heterodimers and cannot functionally substitute for one another (PMID: 22628554, PMID: 22628558).

Response to Reviewers

We thank the reviewers for their careful reading of our revised manuscript. Major changes/ additions to the manuscript text are marked by a line on the right margin. Below is our detailed response to the reviewer critiques and suggestions. In response to these insightful comments, the manuscript more compelling demonstrates the central hypothesis and more clearly presents the data and conclusions.

Reviewer 2

The authors have extensively addressed my comments. I have a few comments/responses back, all relatively minor:

We thank reviewer 2 for their support of our revised manuscript and have addressed their remaining minor points below.

The authors' response to comment 2a is unlikely to be the right answer. Glycolysis is super fast (seconds) and almost certainly at labeling equilibrium. Changes in relative enrichment are thus unlikely to reflect flux, but rather relative contributions to the pool. Higher labeling from external glucose likely reflects less "non-labeling" from internal sources (most likely glycogen). If glycogen pools are unchanged, as stated by the authors, it is possible that glycogen turnover is accelerated, and thus carrying more label, which can be tested by quantifying labeling of the glycogen pool itself. Another possible explanation would involve compartmentalization of fluxes.

We share the reviewer's interest in this result and agree that it was an unexpected finding that the contribution of glucose derived carbons towards the TCA cycle was in fact increased in *Mpc1*^{iCKO} mice. If we left the reviewer with the impression that our opinion was that this likely reflected changes in flux and not equilibrium substrate contributions, we apologize for our prior imprecision and would like to restate our argument. We agree that glycolytic labeling is very fast and at equilibrium in this experiment and therefore we share the belief that changes in glucose labeling into the TCA cycle reflects less 'non-labeling' from other sources that could include glycogen. Since our key data relates to the labeling of pyruvate and lactate (we do not have good data on upper glycolysis to provide more refinement), we believe a reasonable and simple explanation for the changes in labeling of these substrates is *that altered lactate uptake and use accounts for much of the loss of 'non-labeled' substrate* (see extended data figure 1j). As the reviewer suggests, an altered contribution from unlabeled glycogen would also lead to the same result, but we do not have the data to confirm nor rule out that possibility. We speculate that absent a direct

pyruvate sink from the MPC, in *Mpc1*^{iCKO} hearts glycolysis is a larger net contributor to the cardiac pyruvate/ lactate pool and thus suppresses external lactate uptake leading to a larger apparent net contributor of glucose to the cardiac TCA cycle. Overall, we did not include a detailed discussion of this result in the main text as we don't believe that this result materially alters our central hypothesis- namely that there exists an MPC independent mitochondrial carbohydrate carrier in cardiomyocytes. It is beyond the scope of the present manuscript, but we believe that this shift in glycolysis in *Mpc1*^{iCKO} hearts may be causal for some of the hypertrophic phenotypes observed.

The authors' response to comment 4b is a reasonable explanation that is easily tested: does inhibition of MPC increase lactate-to-TCA oxidation and thus respiration, and now CPI-613 would decrease respiration?

Based on our experiments in Extended Data Figure 7q, where we show that MPC inhibition does increase lactate to TCA oxidation in adult cardiomyocytes, we too would expect CPI-613 to counter this rise in respiration observed with UK5099 treatment.

the authors' response to comment 5a is appreciated, and yes, I would include these data in the manuscript, it is an important caveat. The effect is likely not statistically significant because n= only 3-4 / group. i.e., statistically speaking, absence of significant difference does not prove equivalence.

We have included this experiment as a new Extended Data Figure 5i. In our description in the text, we stated clearly that these non-significantly differences do not demonstrate equivalence (lines 203-204).

Reviewer 3

The newly added data substantially strengthen the manuscript, and the authors now provide compelling evidence that mitochondrial lactate metabolism is dependent on MCT1. However, the central claim, direct mitochondrial lactate import, remains insufficiently supported. While the functional, genetic, and localization data strongly suggest a role for MCT1 in enabling lactate oxidation in mitochondria, the manuscript does not provide direct evidence of lactate transport across the mitochondrial membrane. In the absence of a transport assay or an equivalent direct measurement of mitochondrial lactate uptake, the title's claim "Direct mitochondrial lactate import enables cardiac metabolic flexibility under stress" overstates what the data demonstrate. Additional direct transport evidence is needed to fully support this claim.

We appreciate the reviewer's concern that additional evidence for direct mitochondrial transport would further strengthen our conclusions. To address this, we considered which experimental approach would most directly and orthogonally test our central claim that MCT1 mediates direct mitochondrial uptake of lactate in cardiomyocytes. In reviewing both foundational and recent literature and conferring with experts in the field, we concluded that *in situ* plasma membrane permeabilization of adult cardiomyocytes using digitonin or saponin (Kuznetsov et al., 2008, PMID: 18536644) is the most appropriate approach to distinguish plasma membrane transport from mitochondrial transport while preserving cellular architecture and mitochondrial integrity. Saponin permeabilization has been extensively used in primary cardiomyocytes and can be titrated to achieve selective and reversible plasma membrane permeabilization (Johnson et al., 1996, PMID: 8943947). We therefore coupled highly controlled permeabilization with lactate isotope tracing to directly assess mitochondrial lactate metabolism when the plasma membrane barrier is eliminated. The results of these experiments are presented in a new Fig. 2f, Extended Data Fig. 4d–j, and described in lines 189–196. The new methods are described in lines 661-684.

We optimized saponin conditions for selective permeabilization of murine cardiomyocytes using live-cell staining with a membrane-impermeable DNA dye (Cytotox NIR) (Extended Data Fig. 4d). A 5-minute treatment with saponin at 25 µg/mL followed by a 10-minute incubation with dye produced robust nuclear staining, while preserving overall cardiomyocyte morphology in both *Mct1$^{fl/fl}$* and *Mct1^{iCKO}* cells. Higher saponin concentrations resulted in loss of cardiomyocyte structural integrity (Extended Data Fig. 4d). We confirmed selective permeabilization using additional DNA dyes (DAPI) and confocal microscopy (Extended Data Fig. 4e,f), which revealed staining of multinucleated cardiomyocytes only after saponin treatment. Importantly, under these conditions mitochondrial integrity was preserved, as evidenced by maintained mitochondrial membrane potential using MitoTracker dyes (Extended Data Fig. 4e,g) and absence of cytochrome C release by immunoblot (Extended Data Fig. 4h). Together, these data demonstrate plasma membrane permeabilization while preserving mitochondrial integrity throughout the experiment.

Based on prior studies demonstrating that stable isotope tracing can be combined with cellular permeabilization to interrogate mitochondrial metabolism (Nonnenmacher et al., 2017, PMID: 27988388), we performed tracing in permeabilized cardiomyocytes with [U-^{13}C]lactate, as employed previously in intact cells (Extended Data Fig. 4c). Under these conditions, wherein plasma membrane transport is not limiting, ^{13}C-lactate was efficiently converted to pyruvate and subsequently incorporated into TCA cycle intermediates (succinate, fumarate, and malate) at levels comparable to intact cardiomyocytes (Fig. 2f). In contrast, this labeling was markedly attenuated in *Mct1^{iCKO}* cells (Fig. 2f). This effect was

not attributable to genotype-dependent differences in metabolite pool sizes (Extended Data Fig. 4i). Importantly, acute pharmacological inhibition of MCT1 with AZD3965 in *Mct1*$^{fl/fl}$ cells recapitulated this phenotype (Extended Data Fig. 4j). Together, these data demonstrate that mitochondrial lactate metabolism remains dependent on MCT1 even when plasma membrane transport is bypassed.

We contrast our data with recent studies using cultured cell lines that have reported minimal direct contribution of lactate to mitochondrial metabolism (PMIDs: 41881014, 37879334). Conversely, our study leverages *in vivo* measurements and primary cardiomyocytes from highly oxidative tissue. Although similar approaches, including plasma membrane permeabilization, are employed, these systems differ fundamentally in metabolic state. Transformed cell lines typically exhibit a glycolytic, Warburg-like phenotype with limited physiological relevance to non-cancerous tissues, whereas adult cardiomyocytes rely heavily on oxidative metabolism that reflects the dominant metabolic state of most tissues within the mammalian organism. Our data furthermore couples *in vitro* and *in vivo* mechanistic studies with the essential functional relevance of lactate oxidation for cardiac function *in vivo*. Our findings underscore the importance of studying lactate metabolism in the physiologically relevant oxidative living system.

In summary, saponin permeabilization of adult cardiomyocytes selectively disrupts plasma membrane integrity while preserving mitochondrial function. Under these conditions, mitochondrial lactate oxidation remains intact and continues to require MCT1 activity, as demonstrated by both genetic and pharmacological inhibition. These findings provide direct evidence that the MCT1-dependent step in lactate utilization occurs at the mitochondria rather than the plasma membrane. This manuscript now has data from intact animals, perfused hearts, isolated and permeabilized cardiomyocytes, MITO-Tag-purified mitochondria, and purified mitochondrial preparations. Across all these orthogonal approaches, loss or inhibition of MCT1 consistently disrupts mitochondrial lactate oxidation without impairing cytosolic lactate import. There is only one model that explains this assembly of data: *Cardiac lactate oxidation requires MCT1-mediated direct mitochondrial lactate import*.

Reviewer 1:

Reviewer 1 provided a critique of our manuscript that we separately addressed in a response via direct correspondence to the editor. We summarize below the key changes we made to address their core substantive issues that have resulted in a stronger manuscript with a clearer message.

1) In response to concerns about lactate uptake in isolated primary adult cardiomyocytes (reflecting a shared concern with Reviewer 3), we performed a new detailed cellular membrane permeabilization study (Fig. 2f and Extended Data Fig. 4d-j). See our detailed response to reviewer 3 above for our discussion of this new experiment.
2) We fixed a typo in the panel labels for Extended Data Figure 5a (original Extended Data Fig. 4c).
3) We have changed our references in the text to figures that show MCT4 abundance by immunoblot to provide more clarity. See lines 158-9, 267.
4) We have revised the wording of what is now line 177-179 as suggested to: "When comparing *Mpc1*^{iCKO} , *Mct1*^{iCKO} and control hearts from young mice, we observed only modest changes in cytosolic and mitochondrial lactate or pyruvate metabolism proteins, suggesting that metabolic adaptations to gene loss were not primarily at the level of enzyme expression."
5) In response to concerns about Proteinase K assays presented in the revised manuscripts we note that these are complex experiments. Proteinase K–based mitochondrial localization assays are inherently sensitive to mitochondrial integrity, membrane permeabilization, tissue source, and enzyme titration, and therefore must be interpreted based on *relative protection patterns* rather than absolute resistance to proteolysis. Performing these assays on mitochondria isolated from primary tissue or frozen human samples, as in our studies, further compounds these challenges, as complete preservation of membrane integrity is not achievable with these sources. Even in published studies using cultured cells, including recent work in high-impact journals, partial degradation of inner mitochondrial membrane (IMM), intermembrane space (IMS), and even matrix proteins under isotonic conditions is commonly observed, with enhanced degradation upon hypotonic swelling or detergent treatment (e.g., PMID: 36690850). Taken together, none of the observations raised in these critiques undermine our conclusions. To simplify our data presentation, we now present the data in Fig. 3a,b (now Fig. 3 b,c) with a reduced set of control proteins that best reflect the proteinase K conditions we used in this assay and aligns with best published practices for this assay. We further removed certain replicate proteinase K assays that were likely over digested from the Extended Data Fig. 6 leaving our highest quality data from mouse heart, purified cardiomyocytes and human patients (Extended Data Fig. 6e-h)
6) We revised the panel labeling for Extended Data Fig. 9j (previously 8j) to clarify that this treatment was done *in vitro*.
7) We added a note on Extended Data Fig. 11d (was 10d) that all non-marked comparisons were not significant after multiple comparisons corrections.

REVIEWER #1

The authors present a large volume of data to make the case that mitochondria can import lactate directly and that this mediated (in part) by MCT1 that is localized to mitochondria. That mitochondria import lactate is not in question, nor that it could be mediated in part by Mct1. Given the large volume of data presented, it is important that the manuscript and all the data presented are evaluated rigorously. The data generally support the author's hypothesis and contribute an important perspective to the field. That said, there are a number of inconsistencies in the data, many of which were pointed out in the prior review and to which the authors have chosen not to respond. Two concerning examples (among others) are summarized below.

We thank the reviewer for their detailed critiques of the manuscript throughout the review process. Their suggestions have led to better experiments and improved data presentation, both of which have strengthened our manuscript. Due to a miscommunication for which we take complete responsibility, we did not provide a complete point-by-point response to this reviewer's previous critique. We apologize for this error on our part. We are encouraged that the reviewer believes that the collected data in our manuscript "*support the author's hypothesis and contribute an important perspective to the field.*" That said, the reviewer raises several minor points which we address below. These critiques clearly DO NOT affect the conclusions of the study, nor do they compromise the vast amounts of orthogonal data collected in support of each key conclusion. Due to the large scale of this study and the work done by a team of many people from different labs across several years, some small methodological inconsistencies have arisen that fully account for the noted discrepancies. Some of these methodological differences were essential to address different questions or to interrogate different biological materials. Overall, we emphasize that the nature of the minor highlighted 'inconsistencies' arises primary from methodological differences between different experiments. In the below point-by-point response we address these differences and where possible, we include additional data to further support the conclusions. We also acknowledge that we could be clearer in the text, figures and figure legends about the specific methodologies employed and have made the appropriate edits to help the reader to fully understand the experiments presented. We believe that this response and the changes we made to improve the manuscript will address the reviewers concerns and clarify the presentation of this important work.

- ***Within the manuscript the authors do not replicate their own data. If you closely examine extended data figure 11 (d) you will observe that no difference in 13C labeling between saline (sham) treated Mct1fl/fl controls and similarly treated Mct1cko mice is seen. This contrasts with the data in Figure 2H, where they show reduced labeling of succinate and malate.***
- ***Figure 4J shows a strong trend towards increased 13C labeling in lactate infused saline (sham) treated Mct1cko hearts relative to Mct1fl/fl controls. This contrasts with data shown in Figure 2(h) and extended data figure 5 (b).***

We appreciate the reviewers detailed engagement with our data and would like to directly address these discrepancies which arise from essential methodological differences between experiments. We stress that the experimental data in question were from infusion experiments conducted on multiple days from independent cohorts of animals (e.g. from several different litters), included males and females and showed strong intra-experiment consistency. However, when comparing data from Extended Data Fig. 11d (now 11e) and Fig. 2h, we need to emphasize that these were NOT the same experiment conducted on two different days. In contrast, these two groups of mice

were in very different physiological states, one free moving and one anesthetized. It is long known that anesthesia has profound effects on glucose (and presumably lactate) metabolism, and it is generally acknowledged to be best practice to study glucose metabolism in the awake mouse (PMID: 20713647). For our data in healthy animals in Fig. 1 and Fig 2., we present glucose and lactate cardiac metabolism data from awake free moving animals. However, in animals with reduced LVEF and in HF, we were unable to place jugular vein catheters necessary for awake/free moving infusions because procedural mortality was very high in these compromised animals. Therefore, motivated by this reviewers' insightful suggestions after our first submission, we directly addressed the metabolism of the failing heart by employing tail vein catheterization in isoflurane anesthetized animals, a method that has been used to study in vivo metabolism in other situations where jugular vein catheterization was not feasible (e.g. pregnant dams; Nature 2022, PMID 35388219).

To understand the discrepancies noted by the reviewer and better define the relevant physiological differences between the awake and anesthetized animals, we are happy to add additional experimental data to what we already presented in Extended Data Fig. 11. In the methods section, the awake vs anesthetized state of animals is clearly stated. Like prior infusion figures, we edited the paper to include a calculated lactate rate of appearance (Ra) for anesthetized animals which measures systemic lactate turnover. The most significant finding is that upon anesthesia, lactate turnover is suppressed ~3-fold relative to awake animals (compare a new Extended Data Fig. 11d panel (below) with data from Extended Data Fig. 1e and 5d).

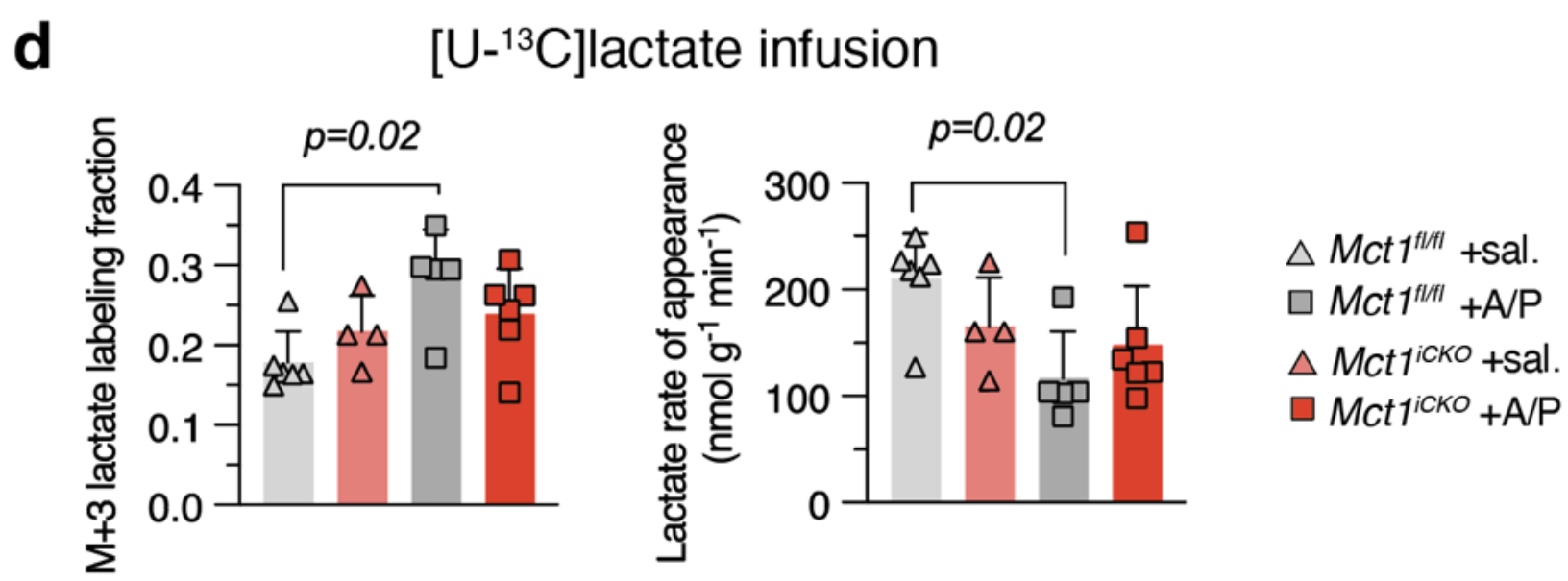

In Ang/PE treated mice, lactate turnover is further depressed in WT but not MCT1 animals. This manifests in part as a measured lower lactate contribution to heart metabolism in the anesthetized compared to free moving animals.

We draw attention to these differences in new text (lines 335-340):

Animals were treated with Ang/PE for 1 week prior to anesthetized tail vein infusions to reduce procedure-induced mortality. Ang/PE treatment led to a rapid increase in the ratio of heart weight to body weight in $Mct1^{iCKO}$ *animals (Extended Data Fig. 11c). Lactate turnover was systemically lower under anesthesia in all conditions compared to awake animals and was further reduced in* $Mct1^{fl/fl}$ *mice subjected to Ang/PE (Extended Data Fig. 11d).*

Given these large differences in systemic lactate metabolism, and the altered contribution of lactate to cardiac metabolism, it is not surprising that loss of MCT1 may have a muted effect in anesthetized animals that have less lactate available and therefore show less lactate assimilation. **The fact that, upon cardiac stress, loss of lactate oxidation is still observed demonstrates that stress elicits a critical non-redundant role for MCT1 in lactate oxidation.**

Finally, the reviewer has more than once critiqued our data presentation in Fig. 4j, suggesting that we should note in the main text an apparent trend towards an increased lactate contribution to cardiac metabolism in saline treated $MCT1^{iCKO}$ hearts relative to $MCT1^{fl/fl}$ controls. The p-value presented is .09 (not significant). The data are also presented as individual TCA metabolite labeling fractions in Extended Data Fig. 11e (same format is in prior figures) and they are also not significant, with no trend apparent (all p-values for the saline $MCT1^{iCKO}$ vs $MCT1^{fl/fl}$ comparison are >.05).

As pointed out in the prior review the authors make statements that partially represent the data. For example, they note in the text that Mpc mRNA is reduced in stressed Mct1cko hearts but fail to systematically acknowledge the protein data, which per the data shown in Figure 4g, appears to be increased at least in one model, and not shown for others. Of course, the authors do not actually quantify this or many other western blots (unclear how many replicates), so readers cannot be certain what the actual changes in protein levels might be. Similarly, relatively small changes in Mpc1 expression are alluded to in their descriptions of models of stressed Mct1cko hearts, which are meaningless in the absence of protein data.

We measured MPC expression by qPCR and or WB in three separate models of HF in $MCT1^{iCKO}$ mice. In 2/3 models, we observed a reduction in MPC1 mRNA transcript in bulk cardiac tissue (TAC and I/R). To acknowledge our contradictory results from different model systems, we have re-worded the relevant results paragraph. Following the reviewer suggestion, we also quantified MPC1 protein expression from our remaining TAC heart lysates and report the results as Ex Data Fig. 10o,p. For the Ang/PE and TAC models, we acknowledge the discrepancy in MPC expression clearly at the start of the relevant results paragraph (lines 310-319):

"To understand the accelerated development of HF in Mct1^{iCKO} *animals, we performed targeted gene expression analysis on Ang/PE and TAC treated hearts. Neither loss of Mct1 nor Ang/PE treatment led to a reduction in Mpc1 gene or protein expression in myocardium extracts (Fig. 4f,g, Extended Data Fig. 9j). Considering that cardiomyocyte nuclei comprise less than 50% of the mouse heart, we then analyzed Mpc1 mRNA levels from purified adult cardiomyocytes and observed a significant reduction upon Ang/PE treatment (Extended Data Fig. 9k). In contrast, TAC treatment led to a reduction in myocardial Mpc1 transcript levels and a trend towards reduced protein as well (Extended Data Fig. 10l,o,p). Slc16a1 and Slc16a3 transcripts were increased in control animals (Extended Data Fig. 10m,n). These data suggest that in some models of HF, MPC1 is suppressed and MCT1 may compensate."*

In the Ang/PE models while MPC1 RNA was not decreased in bulk myocardium, it was from isolated cardiomyocytes treated with the stimuli. It is important to note, however, that this issue of decreased MPC expression is not an important conclusion for the central thesis of our manuscript that MCT1 is an important mediator of mitochondrial lactate import. We've adjusted our wording in the above paragraph to note that.

Overall, the reviewer asks that changes in gene expression are observed in protein expression across several experiments and to increase the quantification of the immunoblot data presented. This is an understandable request. If changes in protein expression because of MCT1 loss supported a central conclusion for the manuscript, this would be necessary for our argument. However, the central conclusions of the paper center on the role of MCT1 for lactate oxidation in

heart and rely on stable isotope tracing data. Nonetheless, we performed an extensive search of our archived samples and were able to obtain new publication quality quantitative western blot data to support the representative immunoblots in Fig. 1j, Fig. 2c and 4g. The new quantifications are presented in Ex Data Fig. 2k,l; Ex Data Fig. 3f-j and Ex Data Fig. 9j. We additionally provided new quantification of TAC heart immunoblots in Ex Data Fig. 10o/p.

This reviewer will not recite again the multiple inconsistencies previously noted in the proteinase K protection assays that remain un-addressed in this revision.

We disagree with the reviewer about the interpretation and quality of our Proteinase K–based mitochondrial localization assays. In this revision, we focused the presentation of these assays on experiments and western blots that are consistent with standards in the field as outlined below. Proteinase K protection assays are inherently sensitive to mitochondrial integrity, membrane permeabilization, tissue source, and enzyme titration, and therefore must be interpreted based on *relative protection patterns* rather than absolute resistance to proteolysis. Performing these assays on mitochondria isolated from primary tissue or frozen human samples, as we are doing in our studies, further compounds these challenges, as complete preservation of membrane integrity is not achievable with such primary biological material. Even in published studies using cultured cells, including recent work in high-impact journals, partial degradation of inner mitochondrial membrane (IMM), intermembrane space (IMS), and even matrix proteins under isotonic conditions is commonly observed, with enhanced degradation upon hypotonic swelling or detergent treatment (e.g., PMID: 36690850 (Nat. Cell Bio. 2023); PMID: 33483504 (Nat. Comm. 2021); PMID: 41408045 (Nat. Comm. 2026). Accordingly, the appropriate comparison for our samples is not whether a protein is fully protected from digestion, but whether its digestion profile most closely resembles that of established compartment-specific controls across experimental conditions. Importantly, all of the Proteinase K datasets in the current manuscript are consistent with our conclusion that MCT1 is localized to the inner mitochondrial membrane. A functional role for mitochondrial MCT1 is independently supported by genetic ablation, mitochondrial immunoprecipitation (MitoTAG), proteomics, and multiple orthogonal functional assays demonstrating an obligate mitochondrial role for MCT1 in lactate oxidation. The proteinase K assays are but one piece of data in support of our hypothesis.

In our experiments, MCT1 consistently displays a digestion pattern that most closely parallels known IMM proteins, showing greater protection under isotonic conditions and increased sensitivity under hypotonic conditions. This pattern is observed across multiple preparations, species, and experimental contexts, including freshly isolated mouse hearts, cultured cell lines, and human heart tissue. Where detergent (Triton X-100) was applied, changes in protein mobility and antibody accessibility—well-documented consequences of membrane solubilization—further influence apparent Proteinase K sensitivity and can vary between species and antibodies (PMID: 37159021).

We will address specific prior comments related to our current Proteinase K blots below:

Across Fig. 3 and Extended Data Fig. 5h, MCT1 consistently tracks with established IMM controls, and these biochemical data align with extensive genetic, proteomic, and functional evidence supporting mitochondrial localization. Partial digestion under isotonic conditions is expected and does not indicate contamination. The samples with the highest isotonic digestion are human samples obtained from frozen tissues (Extended Data Fig 5h) and thus subject to the highest degree of degradation. Human mitochondrial samples were derived from frozen tissue, as fresh human heart tissue was not available to us for obvious practical reasons. Freeze/that cycles are well known to alter mitochondrial membrane integrity and protein accessibility. Prior studies have demonstrated that even detergent-treated IMM and matrix proteins can remain partially resistant to PK digestion depending on protein structure and enzyme titration (PMIDs: 14595396, 41408045). In samples from fresh cultured cells (Extended Data Figure 5g) digestion under isotonic conditions is minimized.

In Fig. 3b, MCT1 exhibits a degree of partial degradation under isotonic conditions that closely parallels that of established IMM/IMS control proteins, including AIF and TIM23, with enhanced degradation under hypotonic conditions. Mitochondrial purity in these preparations was independently validated by immunoblotting (Fig. 2c and 3a; mouse and human respectively) and by proteomic analysis of mitochondrial MCT1 immunoprecipitations (Extended Data Fig. 6a-d), which demonstrate an overwhelmingly mitochondrial origin of recovered proteins.

There are relatively straightforward things that the authors could do to increase confidence in their findings such as showing MCT protein in their mito-tag studies and addressing seeming discrepancies in relative abundance of MCT in mitochondria vs non-mitochondrial fractions (compare figure 1j, 1k, 2c and 4g). Curious why plasma membrane Mct1 is not shown in their KO models, where it would be more abundant.

We thank the reviewer for their careful analysis of these western blots. This discrepancy (between Fig. 1j,1k, 2c and 4g) arose from the use of two different protocols for cellular fractionation by two different members of our team over the several years we worked on this project. In Fig. 1k and 2c, we performed a different ultracentrifugation step to deplete membranes from the cytoplasmic fraction that was not done in for the western blots presented in Fig. 1j and 4g. This added membrane depletion resulted in the loss of plasma membrane MCT1 signal as expected. We did not make this methodological change as clear in the text as we should have done. To remove the possibility of misinterpretation, we have removed Fig. 1k as it conveys redundant information with Fig. 1j and replaced Fig. 2c with the below western blot that was run under the same protocol as

western blots in Fig. 1j and 4g. This will ensure that all mouse-derived fractionation data presented came from the same protocol.

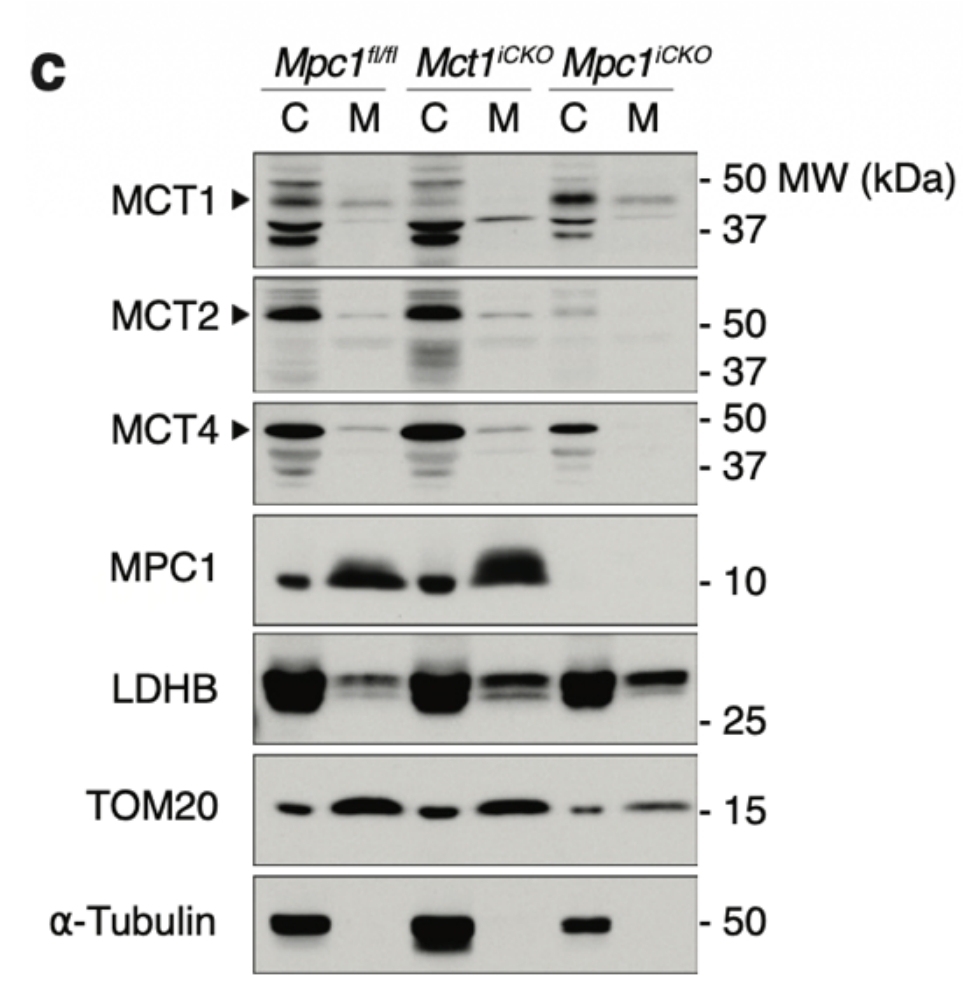

As stated above (lines 129-138), we did an extensive search through our archived samples and were able to obtain publication quality quantitative western blot data to support the representative immunoblots in Fig. 1j, Fig. 2c and 4g. The new quantifications are presented in Ex Data Fig. 2k,l; Ex Data Fig. 3f-j and Ex Data Fig. 9j. We additionally provided new quantification of TAC heart immunoblots in Ex Data Fig. 10o/p. We were unable to find adequate material to re-run the experimental MitoTag samples for MCT1 protein.

Relevant phrasing regarding the new data are:

Line 159: *Interestingly, MCT1 expression was increased in the mitochondria of young $Mpc1^{iCKO}$ hearts but not failing hearts (Extended Data Fig. 2i,k,l).*

Lines 178-184: Across genotypes, a larger fraction of MCT1 signal was associated with mitochondria than MCT2 or MCT4 (Fig. 2c, Extended Data Fig. 3f-h). When comparing $Mpc1^{iCKO}$, $Mct1^{iCKO}$ and control hearts from young mice, we observed an increase in cytosolic and total MCT1, MCT4 and LHDB protein expression in $Mpc1^{iCKO}$ hearts suggesting a lactate metabolism compensatory axis (Extended Data Fig.3f,h,i). No significant changes in the expression of these proteins or MPC1 and MCT2 were observed in $Mct1^{iCKO}$ hearts (Extended Data Fig 3f-j).

Data interpretation - The authors state: "Upon Ang/PE treatment, metabolite changes inMct1iCKO hearts were of greater magnitude and NADH was the most upregulated metabolite, suggestive of increased lactate and impaired oxidation in these animals (Fig. 4i)." A more likely explanation for the increase in NADH in stressed Mct1cko hearts is likely increased glycolysis coupled with impaired oxidative phosphorylation.

There is not a substantive disagreement in the interpretation of the data. We agree with the reviewer that the measured increase in NADH reflects impaired oxidative phosphorylation. To make this eminently clear in the text, we will edit the relevant passage to read:

"Upon Ang/PE treatment, metabolite changes in *$Mct1^{iCKO}$* hearts were of greater magnitude and NADH was the most upregulated metabolite, *suggesting an impairment in oxidative phosphorylation."*

Regarding whether NADH arises from glycolytic flux or impairment of NADH oxidization from other sources (Fatty acid oxidation, beta-hydroxybutyrate dehydrogenase, or lactate dehydrogenase or

others), we don't have independent experimental data to distinguish these possibilities and don't think that speculating on this tangential point is appropriate.

Other data are curious and not interpreted or addressed: e.g. potential reasons for lactate /pyruvate labeling not being increased in AZD treated Mpc1Ko animals.

In this critique and in the prior revision, the referee highlighted some observations from our data that we chose not to explicitly discuss in the text of the manuscript as they don't appear to be highly relevant to the interpretation of the core experiment. In the cited experiment (Fig. 2a and Extended Data Fig. 3), in addition to a sustained decreased labeling of multiple TCA cycle intermediates between WT and KO animals systemically treated with the AZD MCT1 inhibitor, we observed an increase in cardiac lactate labeling upon AZD inhibitor treatment that only occurred in the WT animals. As this difference was not represented in the labeled pyruvate fraction, it likely reflects a minor perturbation in lactate that we don't observe in the knockout animals. Given that our normalization strategy between mice relies on serum glucose labeling and that pyruvate labeling was unchanged (see Extended Data Fig. 3a), we cannot think of a scenario wherein this minor difference would alter the interpretation of the data. We further highlight that in the uncorrected data presented in Extended Data Fig. 3, the same clear trends are present showing a decrease in TCA cycle labeling from glucose only in AZD treated KO animals and in no other group, clearly consistent with the conclusions that we presented.

REVIEWER #2

I find that the authors have provided overwhelming evidence in support of their thesis. Could more studies be carried out? Of course, always. Maybe, for example, studies with reconstituted liposomes of mitochondrial membranes (perhaps what Rev3 is suggesting). However, such studies are not trivial and may not work technically, and as outlined in the authors response to Rev3, at this point there is little alternative interpretation to the overwhelming data provided than that cardiac lactate oxidation requires MCT1-mediated direct mitochondrial lactate import. I would argue that it is time to let the field interpret these compelling and important data.

We thank the reviewer for their support for our revised manuscript.

REVIEWER #3

I thank the authors for the additional work performed during this revision, which I recognize is technically demanding in cardiomyocytes. I also appreciate that direct demonstration of mitochondrial lactate import is genuinely difficult.

The new genetic, pharmacological, localization, and permeabilization data support the conclusion that cardiac lactate oxidation requires MCT1, and that the MCT1-dependent step occurs downstream of plasma membrane transport. The saponin experiments, in particular, address my prior concern about distinguishing plasma membrane from mitochondrial contributions.

However, without a direct transport assay, such as radiolabeled lactate uptake into purified mitochondria, or reconstitution of MCT1 into proteoliposomes, the data do not formally demonstrate that MCT1 itself transports lactate across the inner mitochondrial membrane. Thus, while direct mitochondrial lactate import may be the most parsimonious interpretation, the current title, "Direct mitochondrial lactate import enables cardiac metabolic flexibility under stress," overstates what the data directly demonstrate. I suggest revising the title and

limiting the phrase "direct mitochondrial lactate import" throughout the manuscript. More evidence-aligned alternatives include: "MCT1-dependent mitochondrial lactate oxidation enables cardiac metabolic flexibility under stress" or "Mitochondrial MCT1 enables cardiac metabolic flexibility under stress."

The authors can still present direct import as their preferred model in the Discussion. With these changes, the manuscript's claims would better match the data.

We are happy that reviewer 3 acknowledges the new data in this revision and they agree that our explanation is the most parsimonious to match the data. Given their continued concerns about definitive proof for 'direct import', we are happy to change the title as suggested. We will also change the phrasing throughout the text to de-emphasize 'direct' import. To keep within the 75-character word limit for *Nature*, we propose "***MCT1-dependent mitochondrial lactate import sustains cardiac metabolism***" as the new manuscript title.
